# Supplementary figures and images for: Styxl2 regulates de novo sarcomere assembly by binding to non-muscle myosin IIs and promoting their degradation
Source: eLife. 2024 Jun 3;12:RP87434. doi: 10.7554/eLife.87434 (PMC11147509; doi:10.7554/eLife.87434)

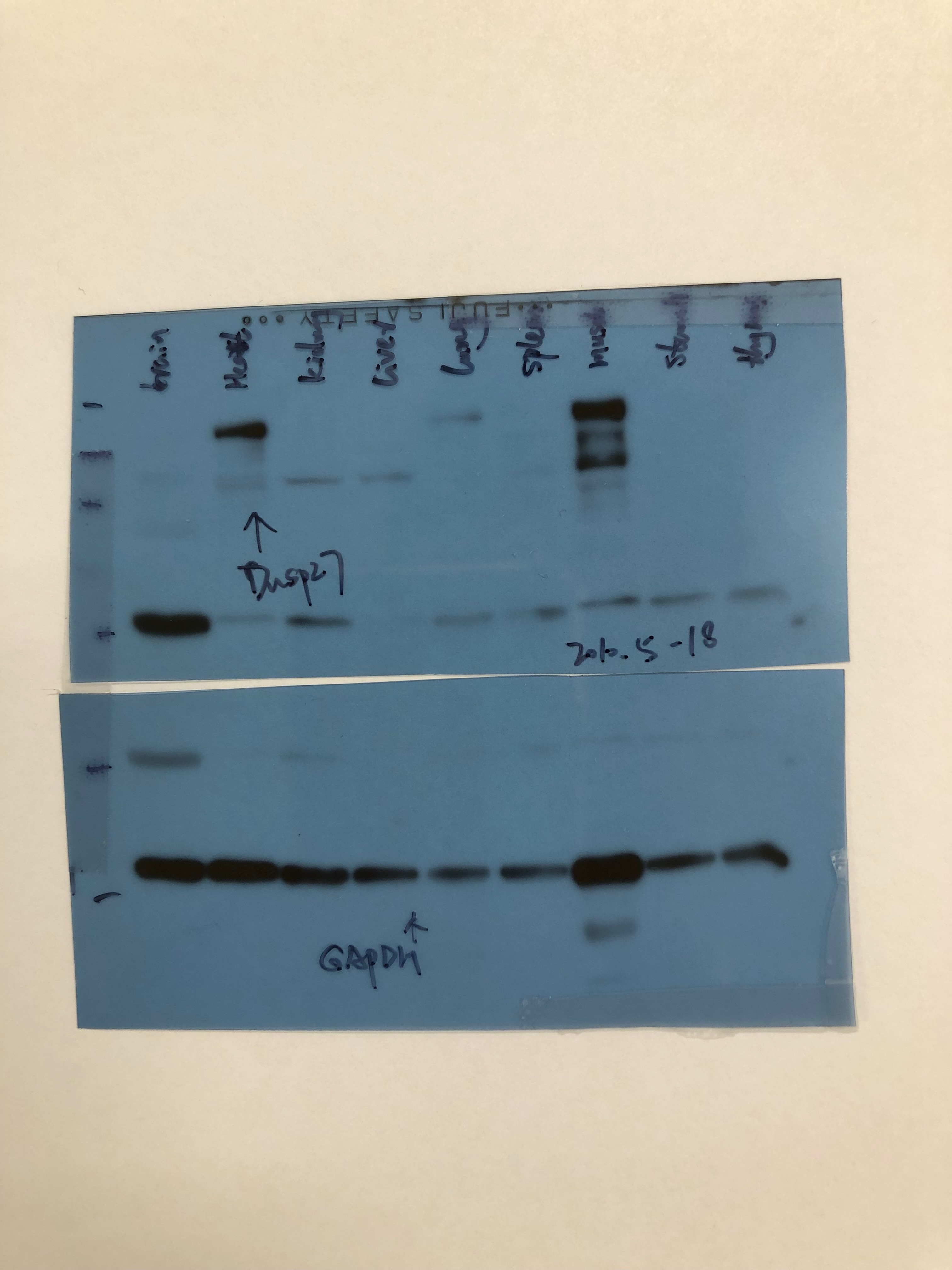

Supplement: Figure 1—source data 1. [file elife-87434-fig1-data1.zip › Figure1B.jpeg]

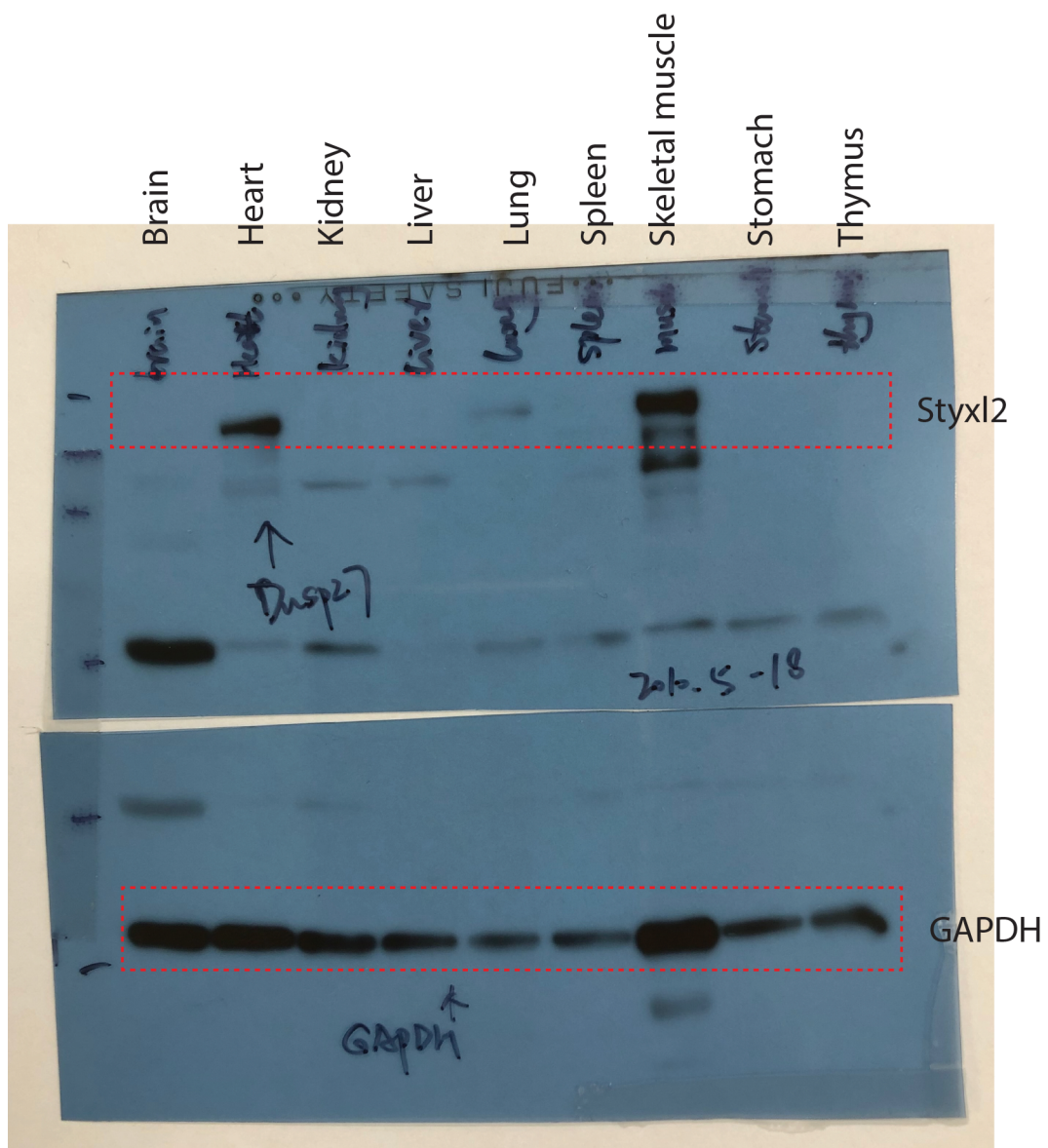

Supplement: Figure 1—source data 2. [file elife-87434-fig1-data2.zip › Figure1B.pdf]

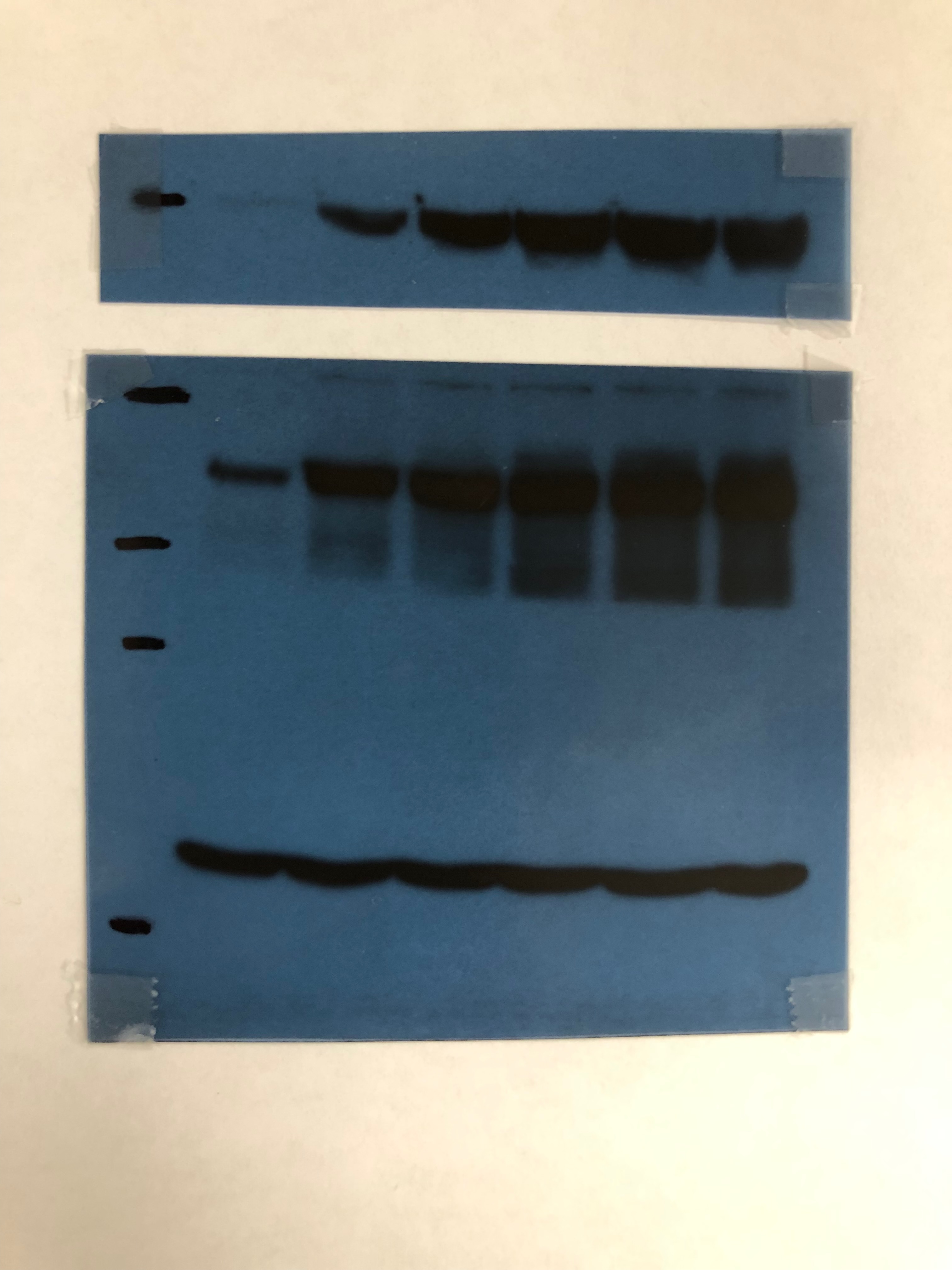

Supplement: Figure 1—source data 3. [file elife-87434-fig1-data3.zip › Figure1C.jpeg]

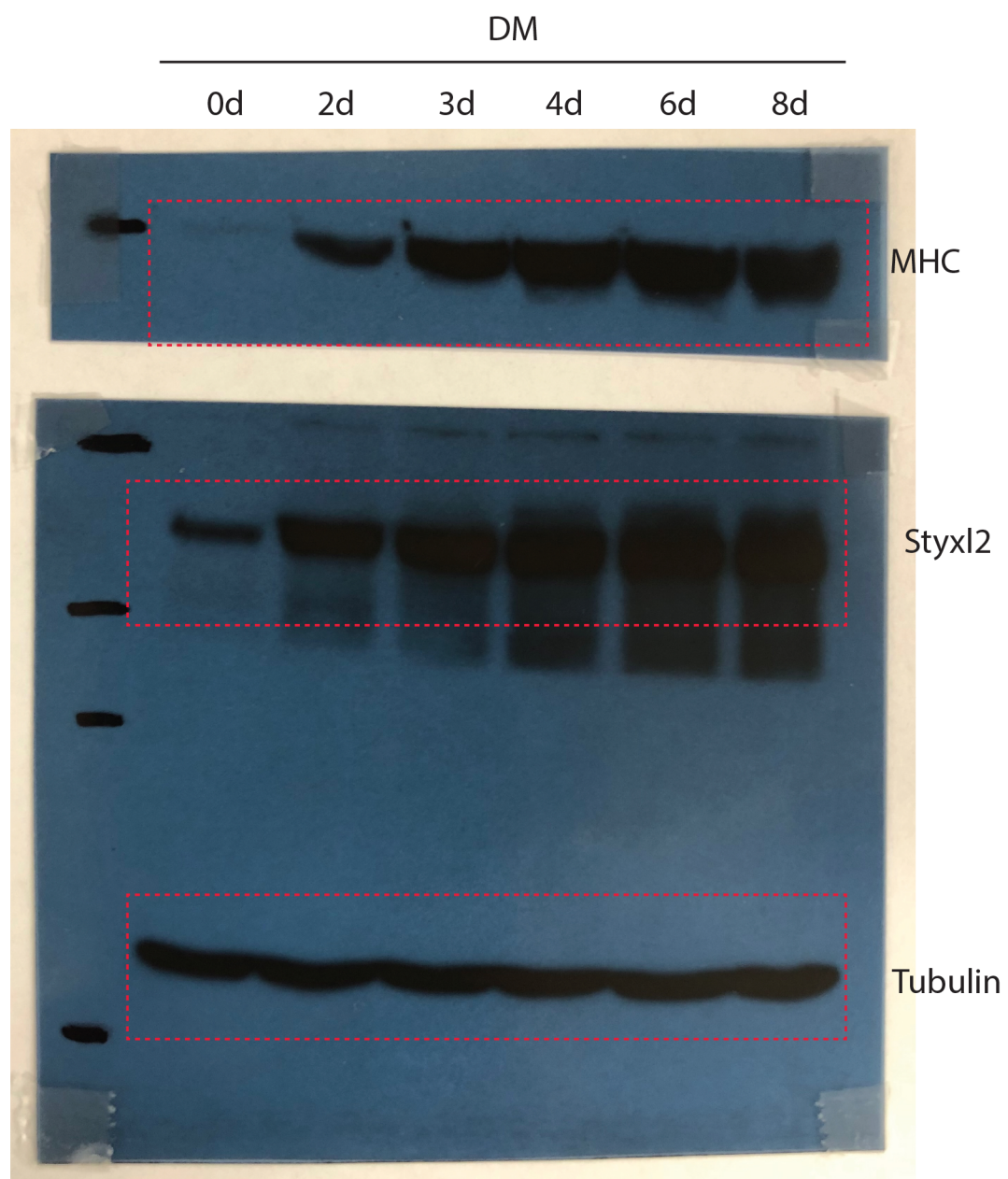

Supplement: Figure 1—source data 4. [file elife-87434-fig1-data4.zip › Figure1C.pdf]

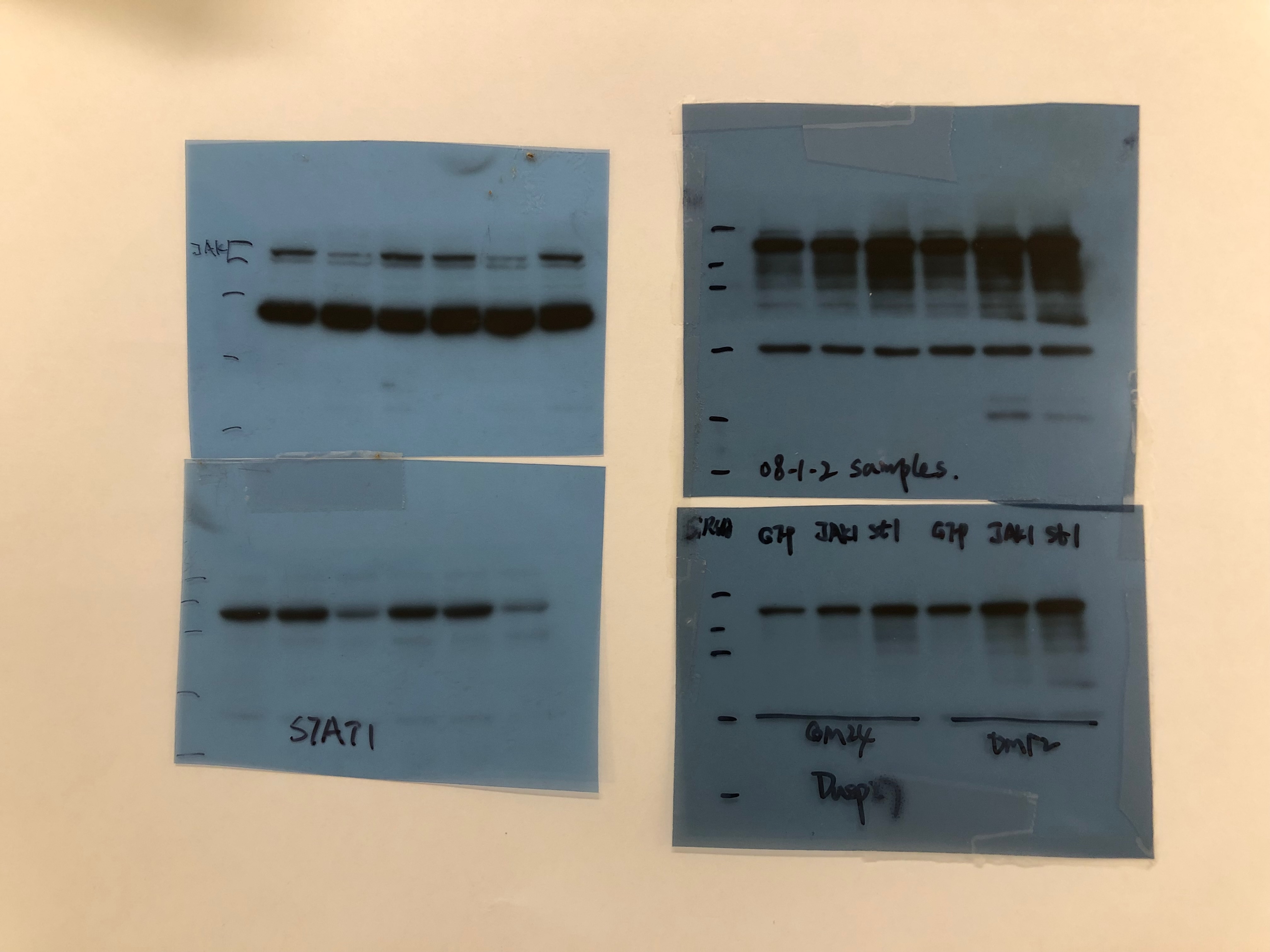

Supplement: Figure 1—figure supplement 1—source data 1. [file elife-87434-fig1-figsupp1-data1.zip › Figure1S1C.jpeg]

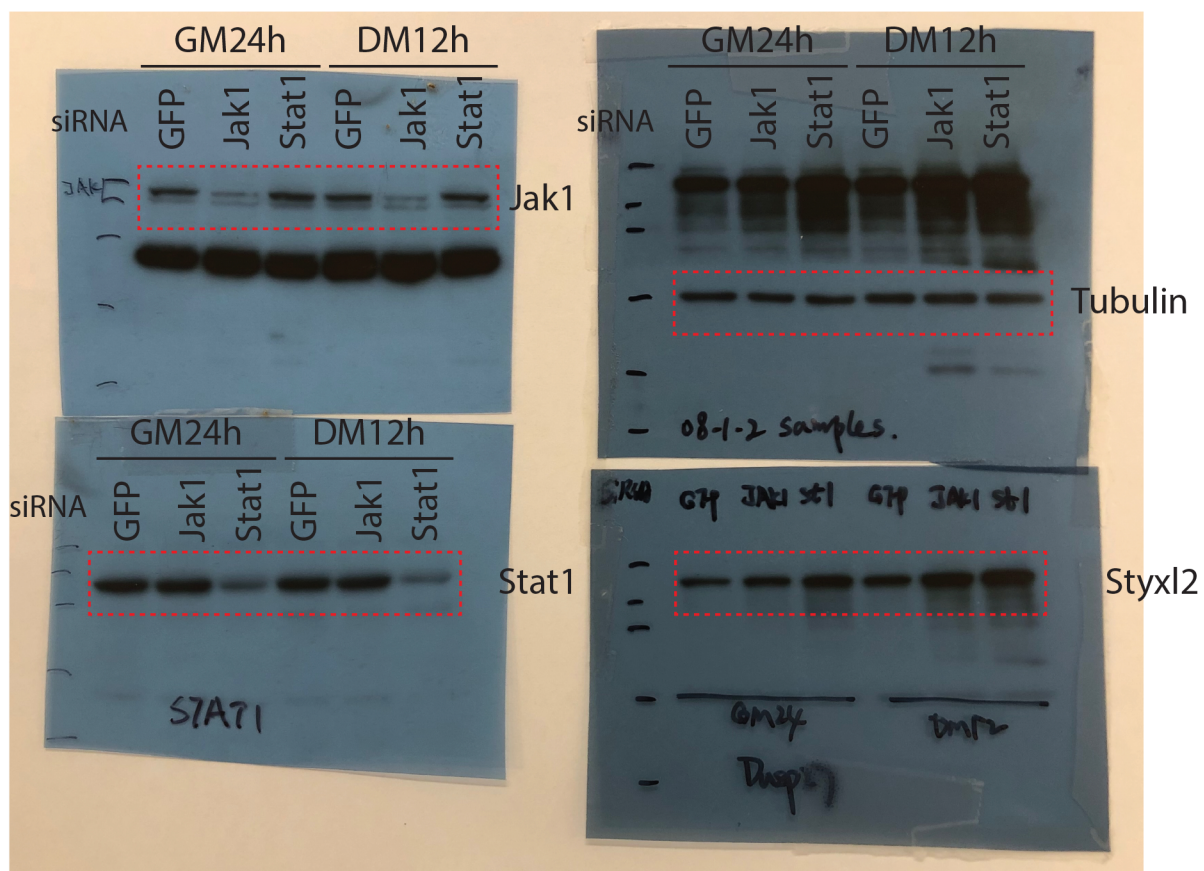

Supplement: Figure 1—figure supplement 1—source data 2. [file elife-87434-fig1-figsupp1-data2.zip › Figure1S1C.pdf]

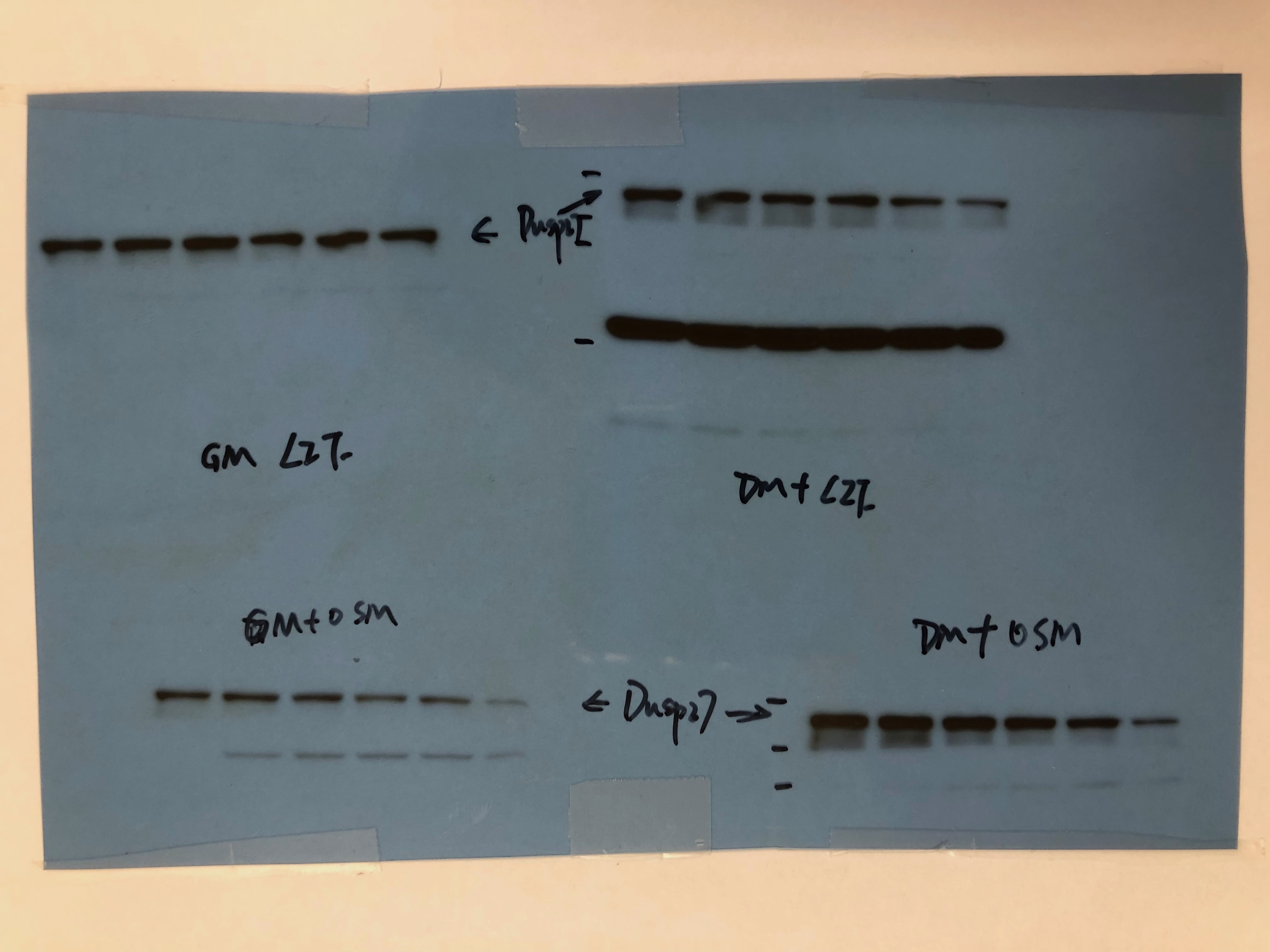

Supplement: Figure 1—figure supplement 1—source data 3. [file elife-87434-fig1-figsupp1-data3.zip › Figure1S1D.jpeg]

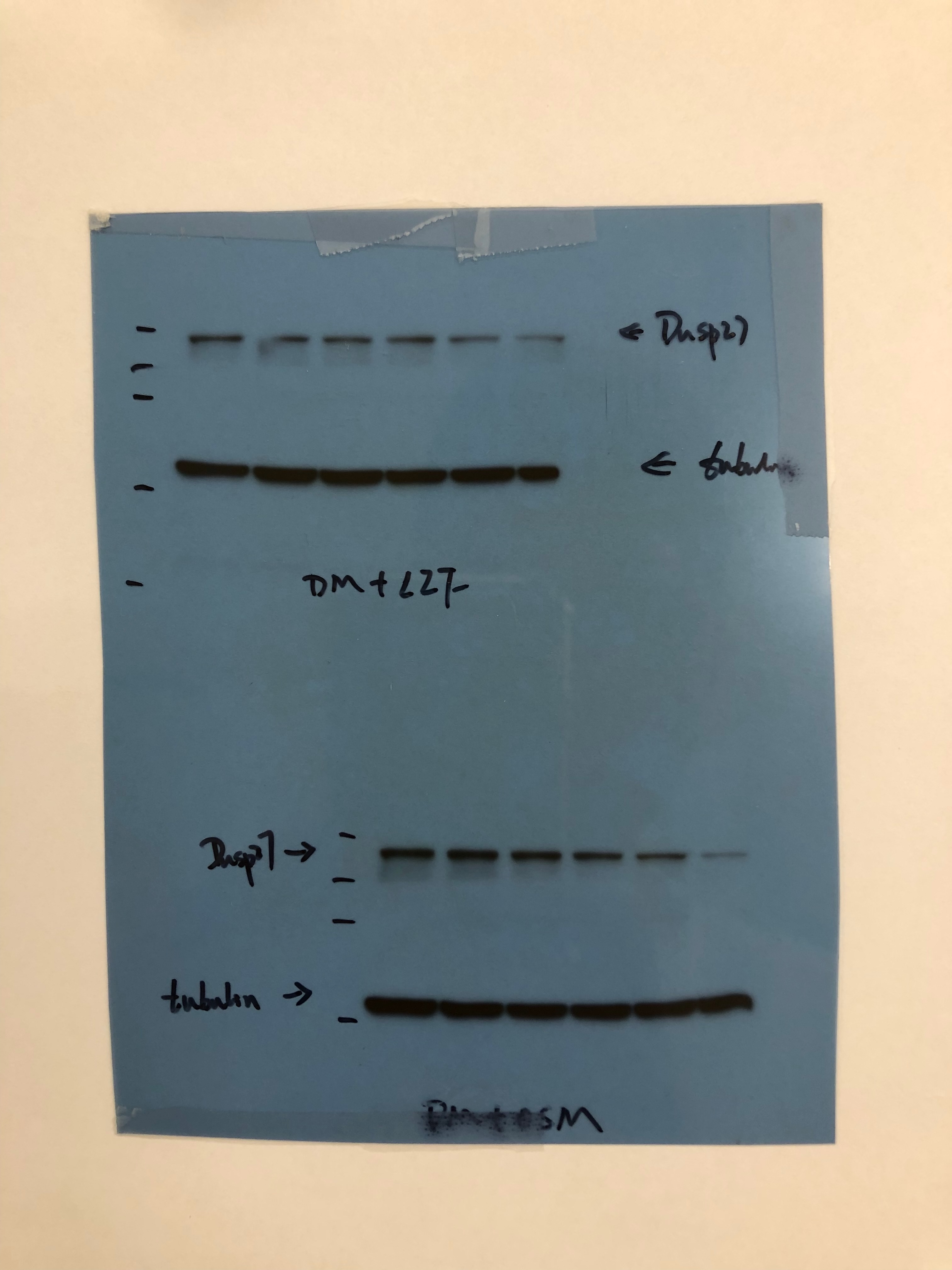

Supplement: Figure 1—figure supplement 1—source data 4. [file elife-87434-fig1-figsupp1-data4.zip › Figure1S1DE.jpeg]

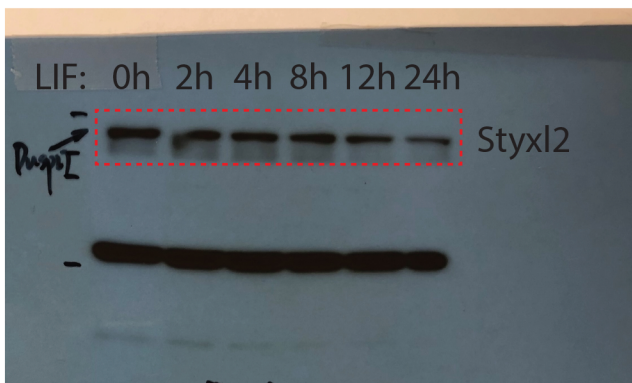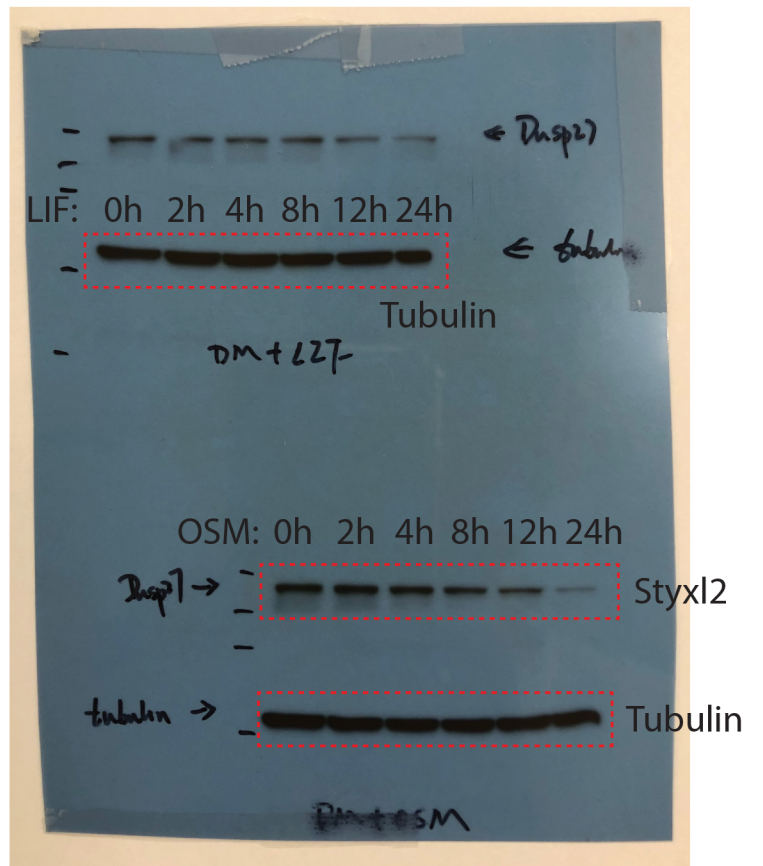

Supplement: Figure 1—figure supplement 1—source data 5. [file elife-87434-fig1-figsupp1-data5.zip › Figure1S1DE.pdf]

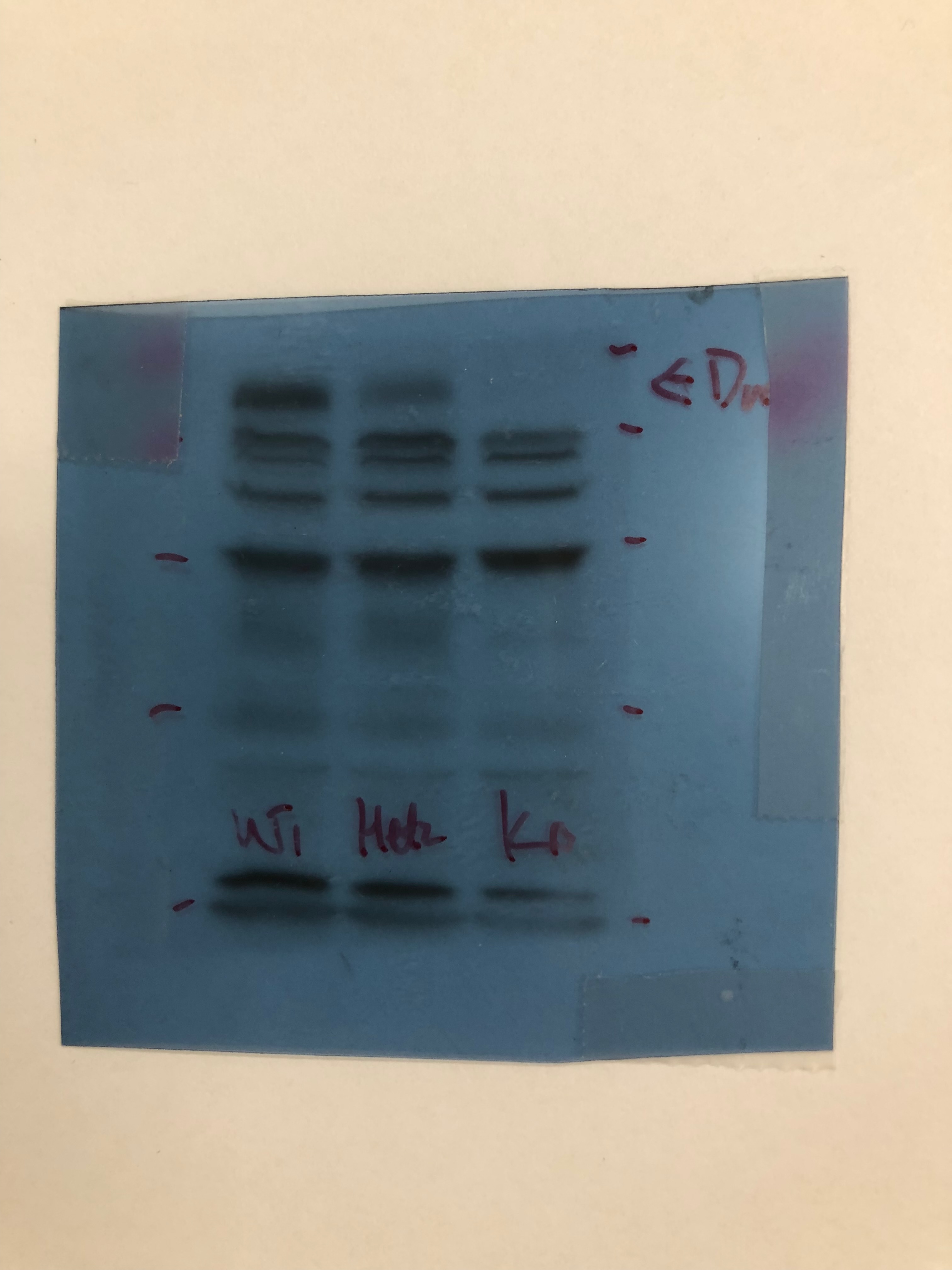

Supplement: Figure 2—source data 1. [file elife-87434-fig2-data1.zip › Figure2A.jpeg]

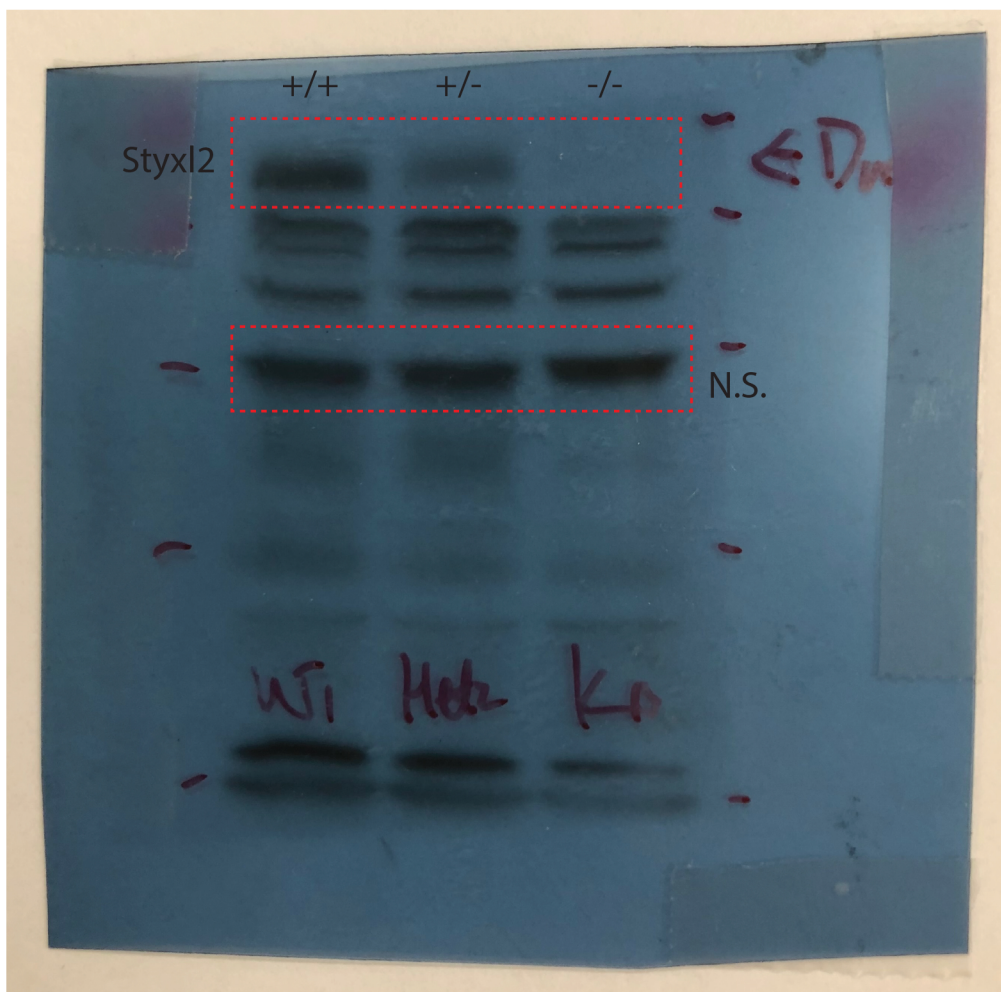

Supplement: Figure 2—source data 2. [file elife-87434-fig2-data2.zip › Figure2A.pdf]

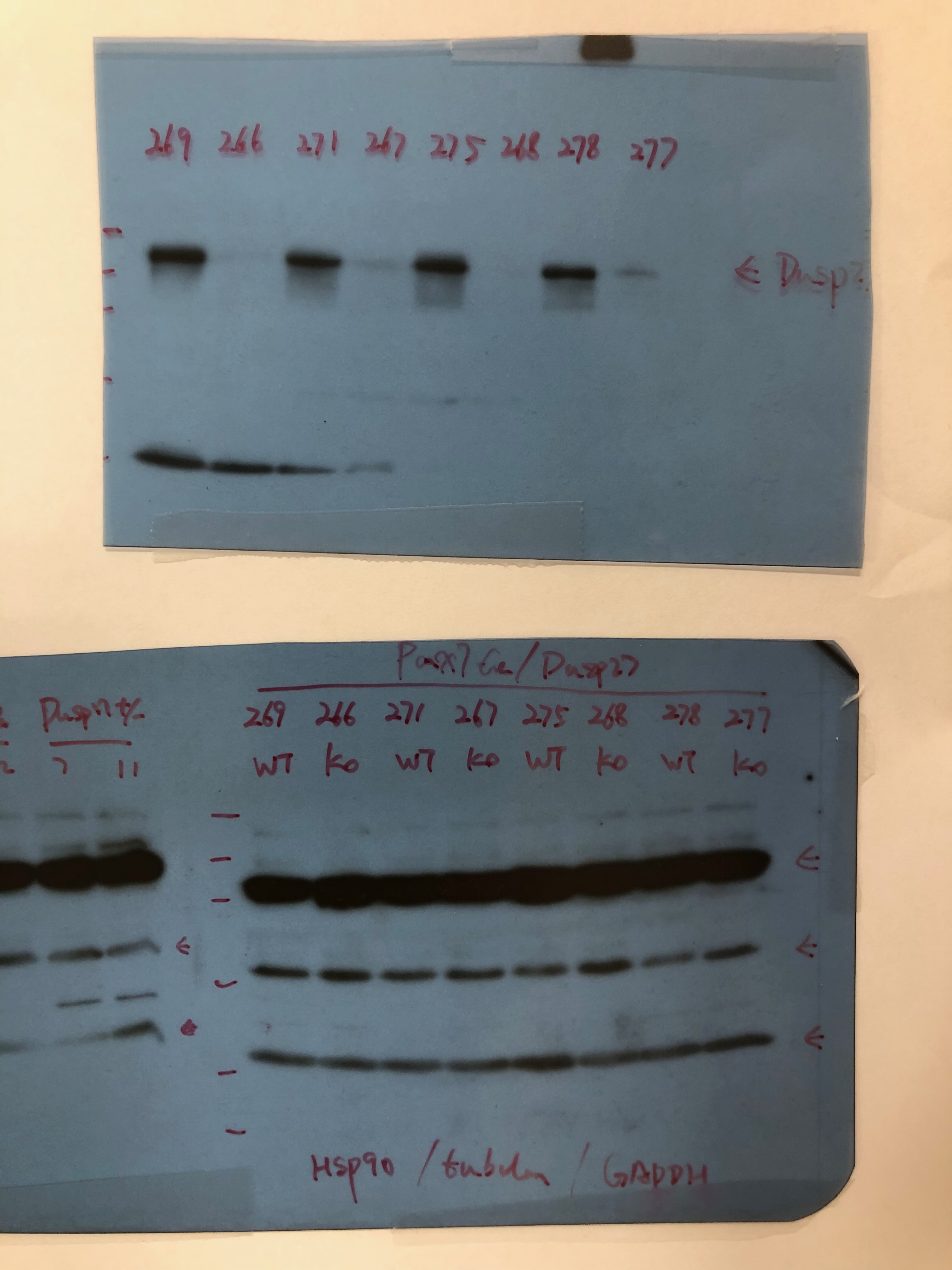

Supplement: Figure 2—source data 3. [file elife-87434-fig2-data3.zip › Figure2D.jpeg]

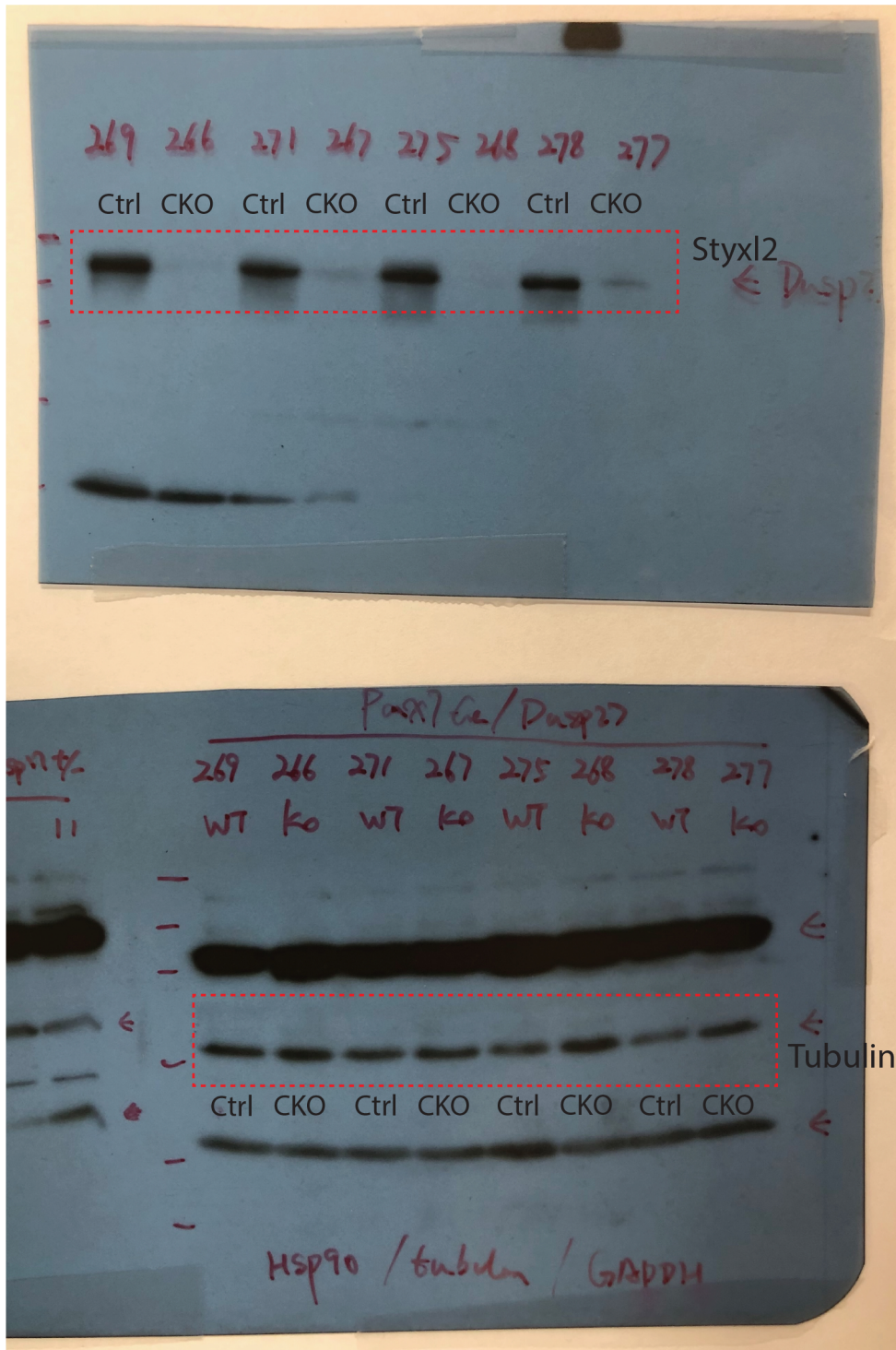

Supplement: Figure 2—source data 4. [file elife-87434-fig2-data4.zip › Figure2D.pdf]

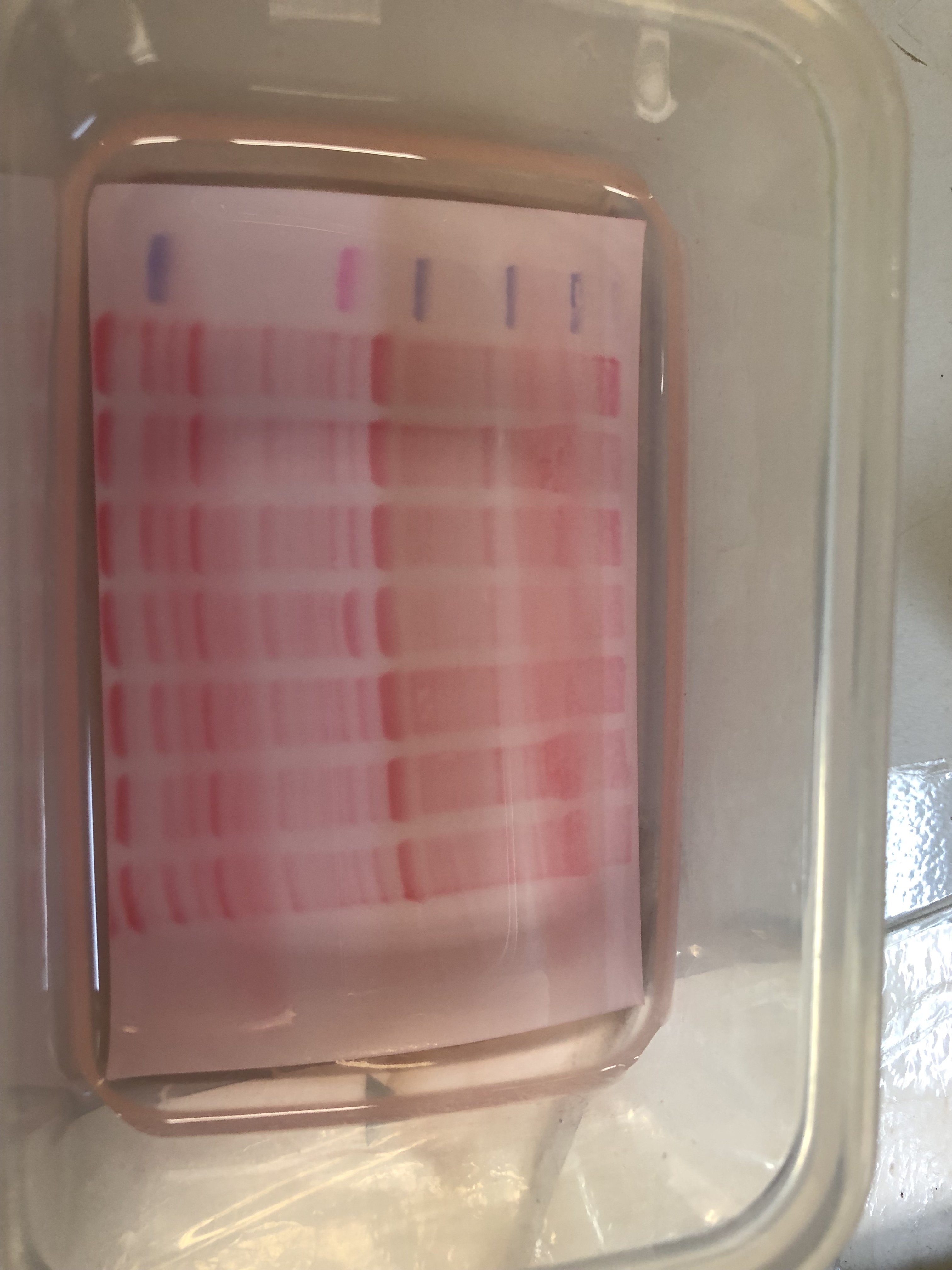

Supplement: Figure 3—source data 1. [file elife-87434-fig3-data1.zip › Figure3B/Figure3B-2.jpg]

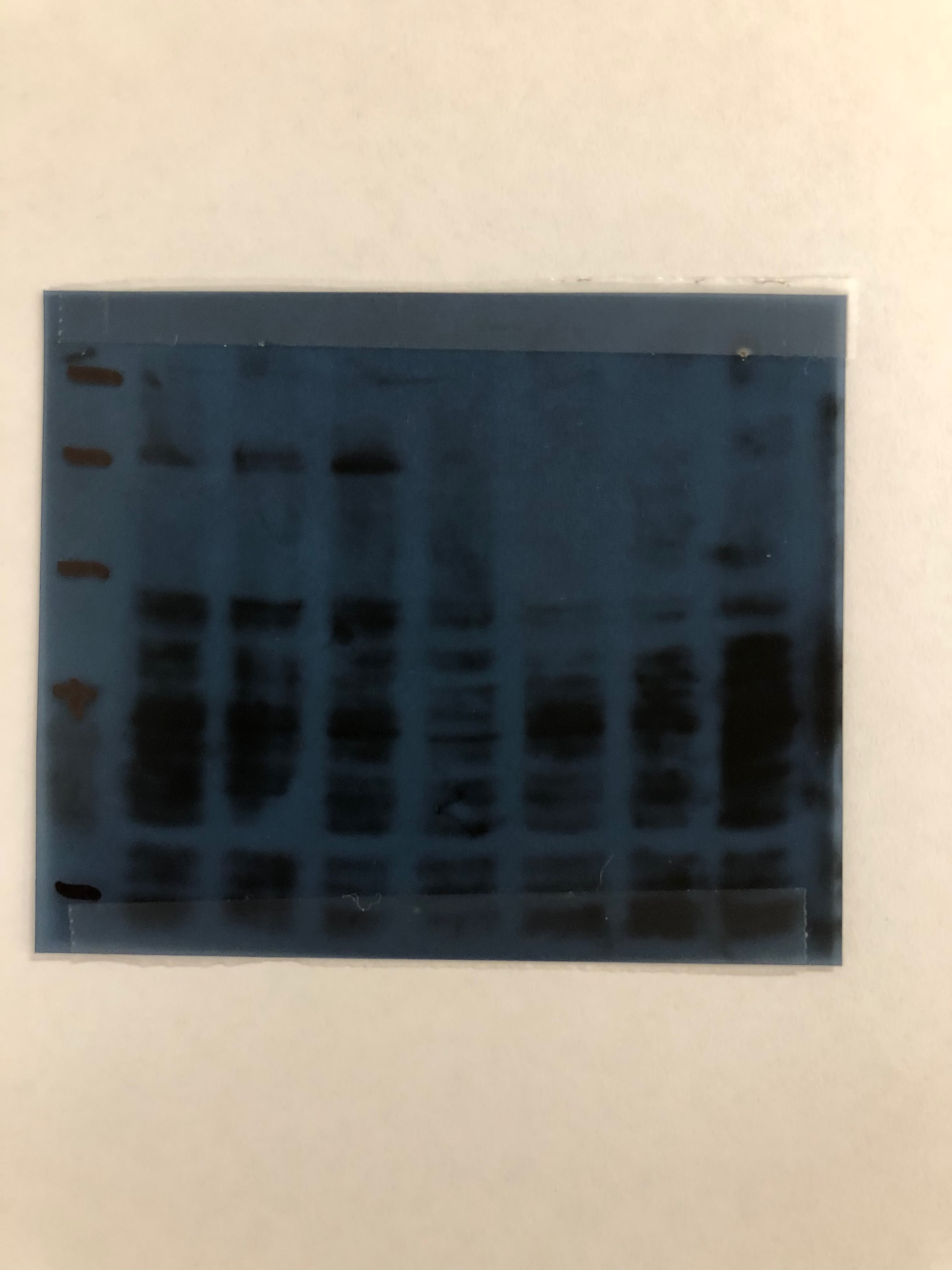

Supplement: Figure 3—source data 1. [file elife-87434-fig3-data1.zip › Figure3B/Figure3B-1.jpeg]

Ctrl

MF-iKO

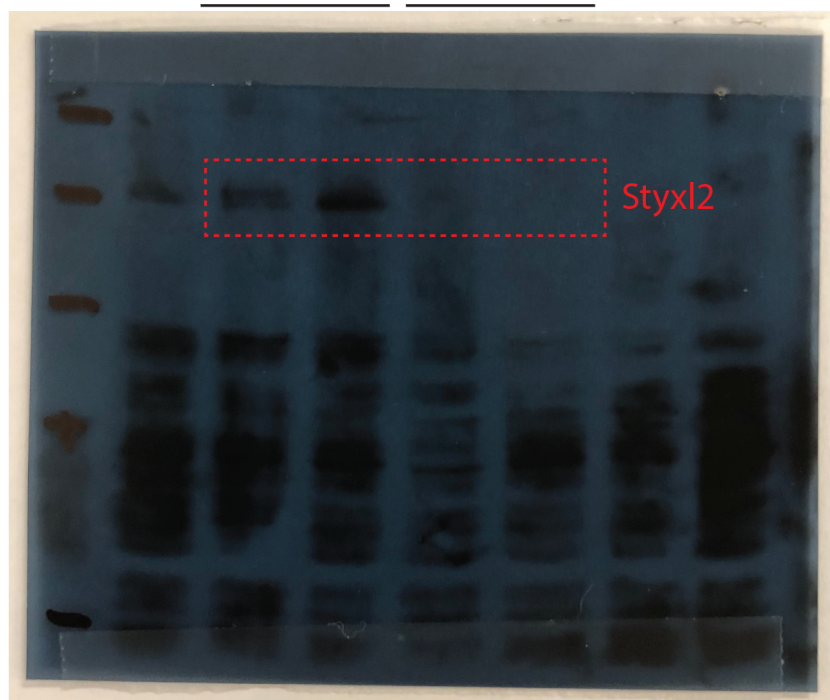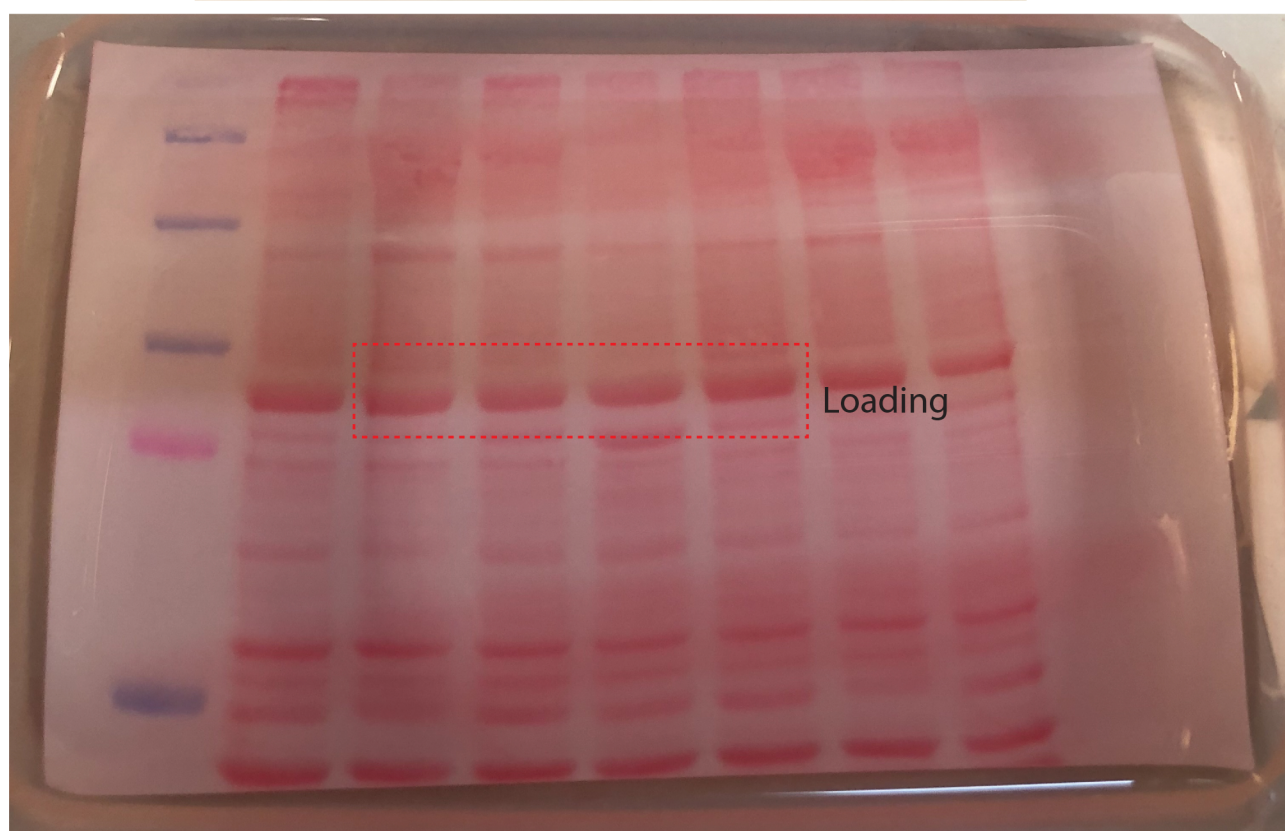

Supplement: Figure 3—source data 2. [file elife-87434-fig3-data2.zip › Figure3B.pdf]

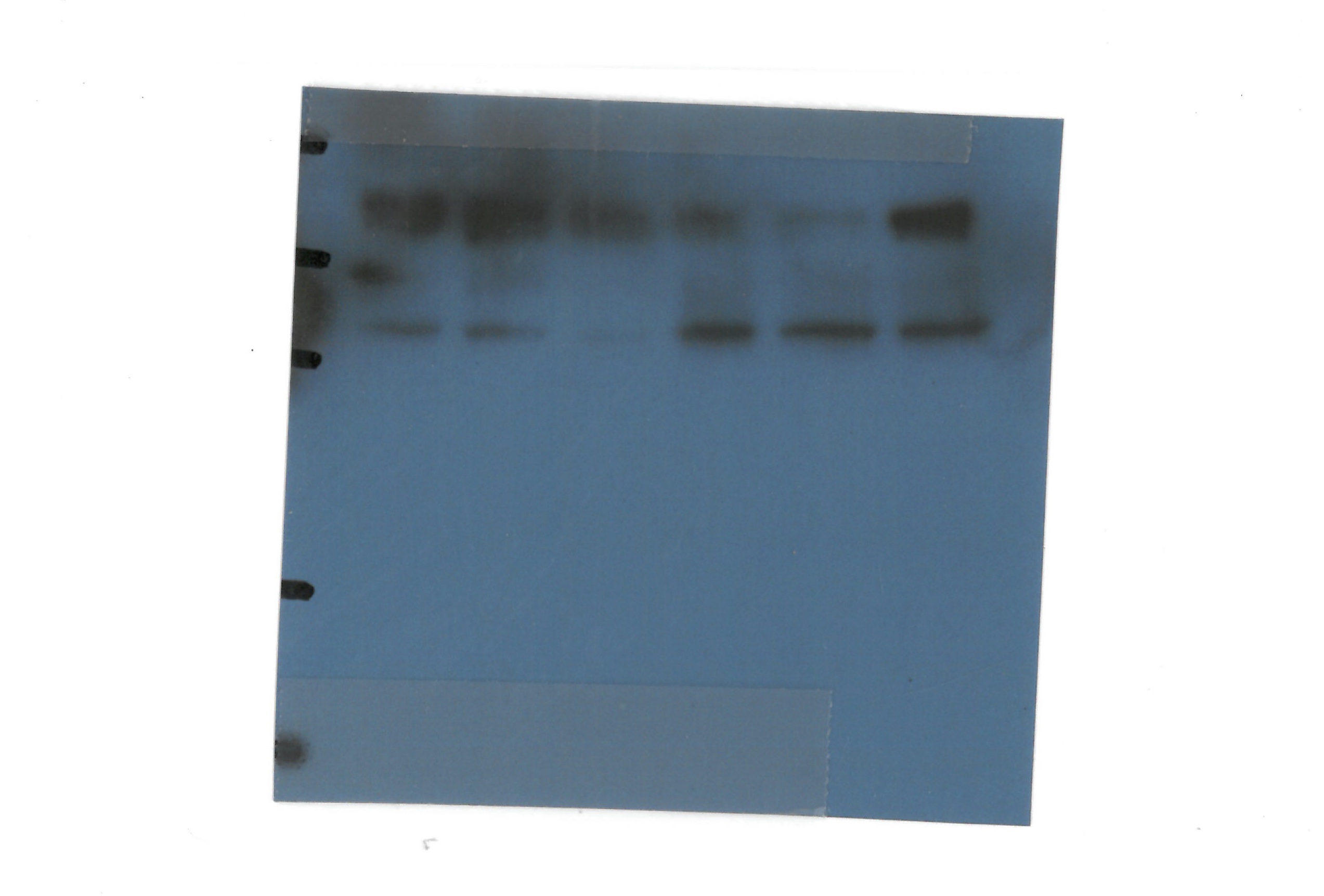

Supplement: Figure 3—source data 3. [file elife-87434-fig3-data3.zip › Figure3F.jpeg]

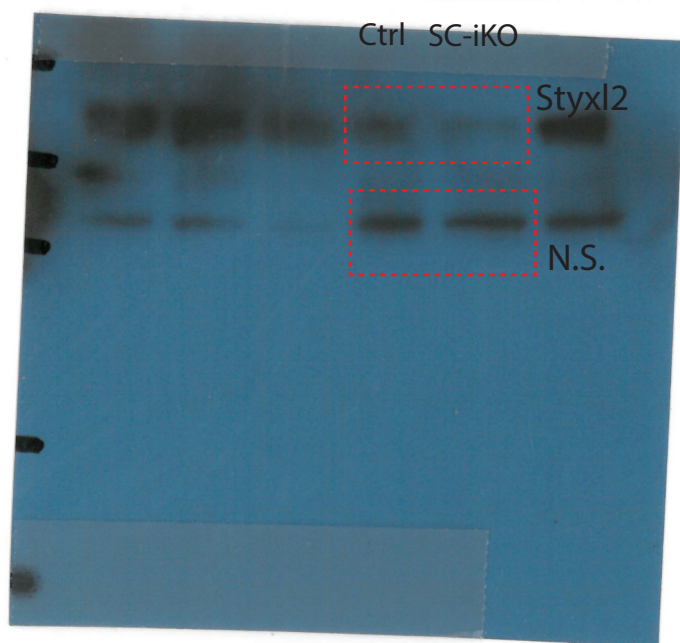

Supplement: Figure 3—source data 4. [file elife-87434-fig3-data4.zip › Figure3F.pdf]

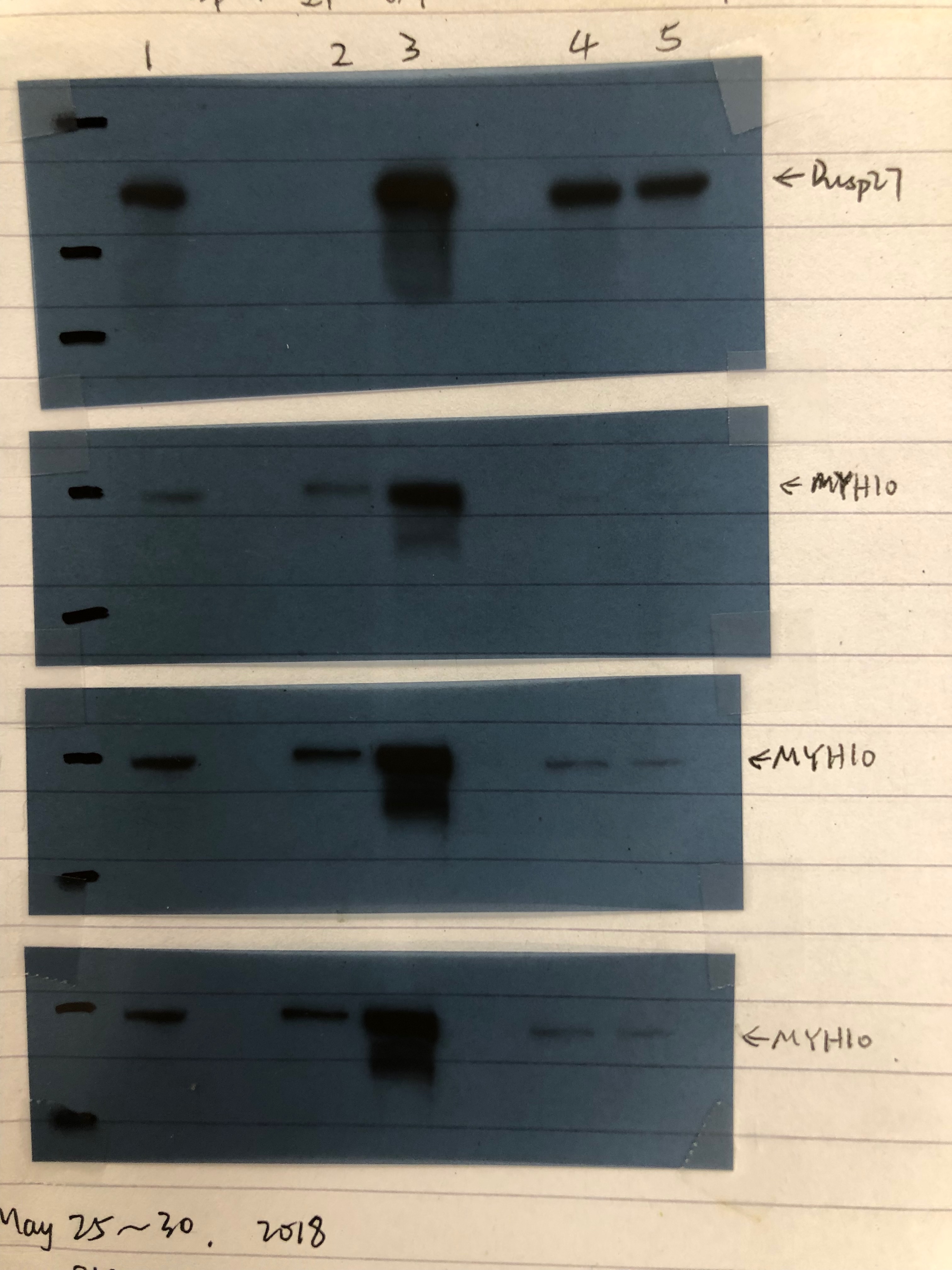

Supplement: Figure 4—source data 1. [file elife-87434-fig4-data1.zip › Figure4D/Figure4D-2.jpeg]

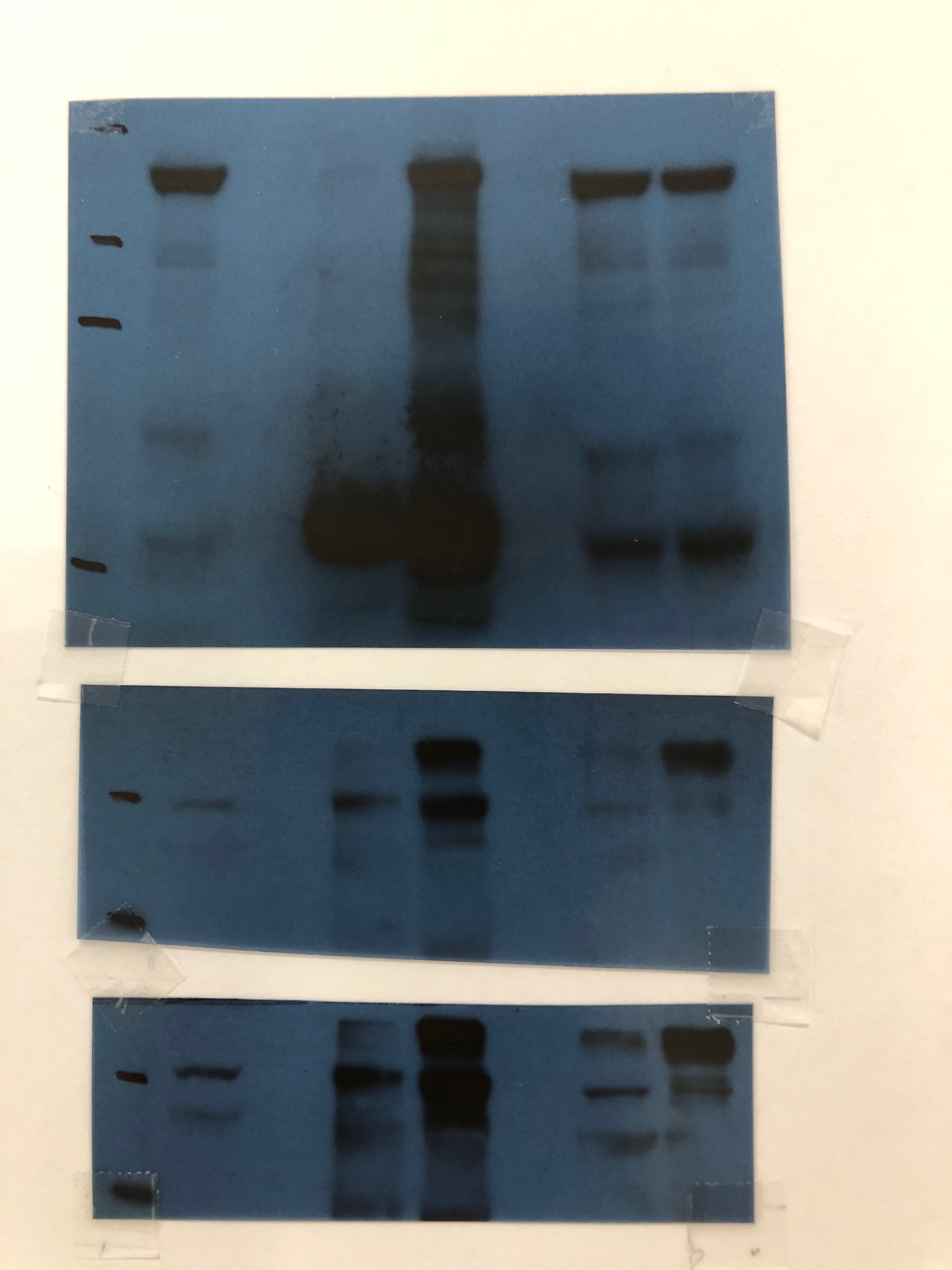

Supplement: Figure 4—source data 1. [file elife-87434-fig4-data1.zip › Figure4D/Figure4D-1.jpeg]

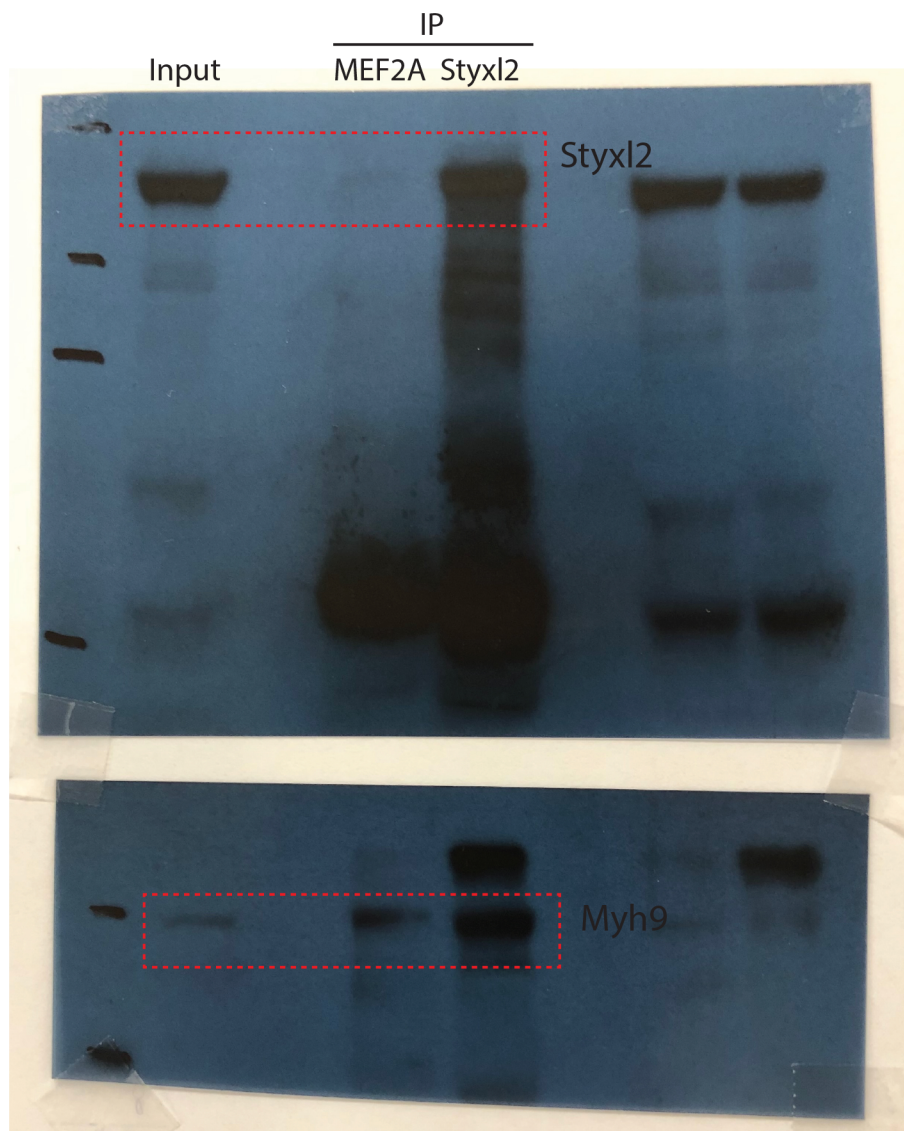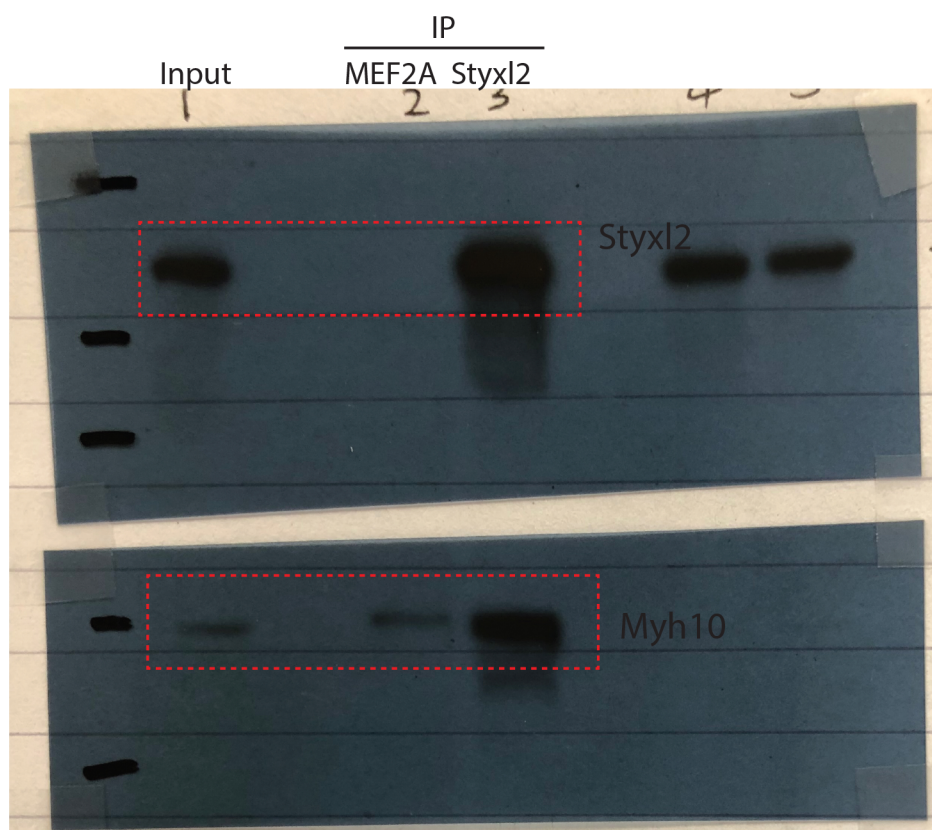

Supplement: Figure 4—source data 2. [file elife-87434-fig4-data2.zip › Figure4D.pdf]

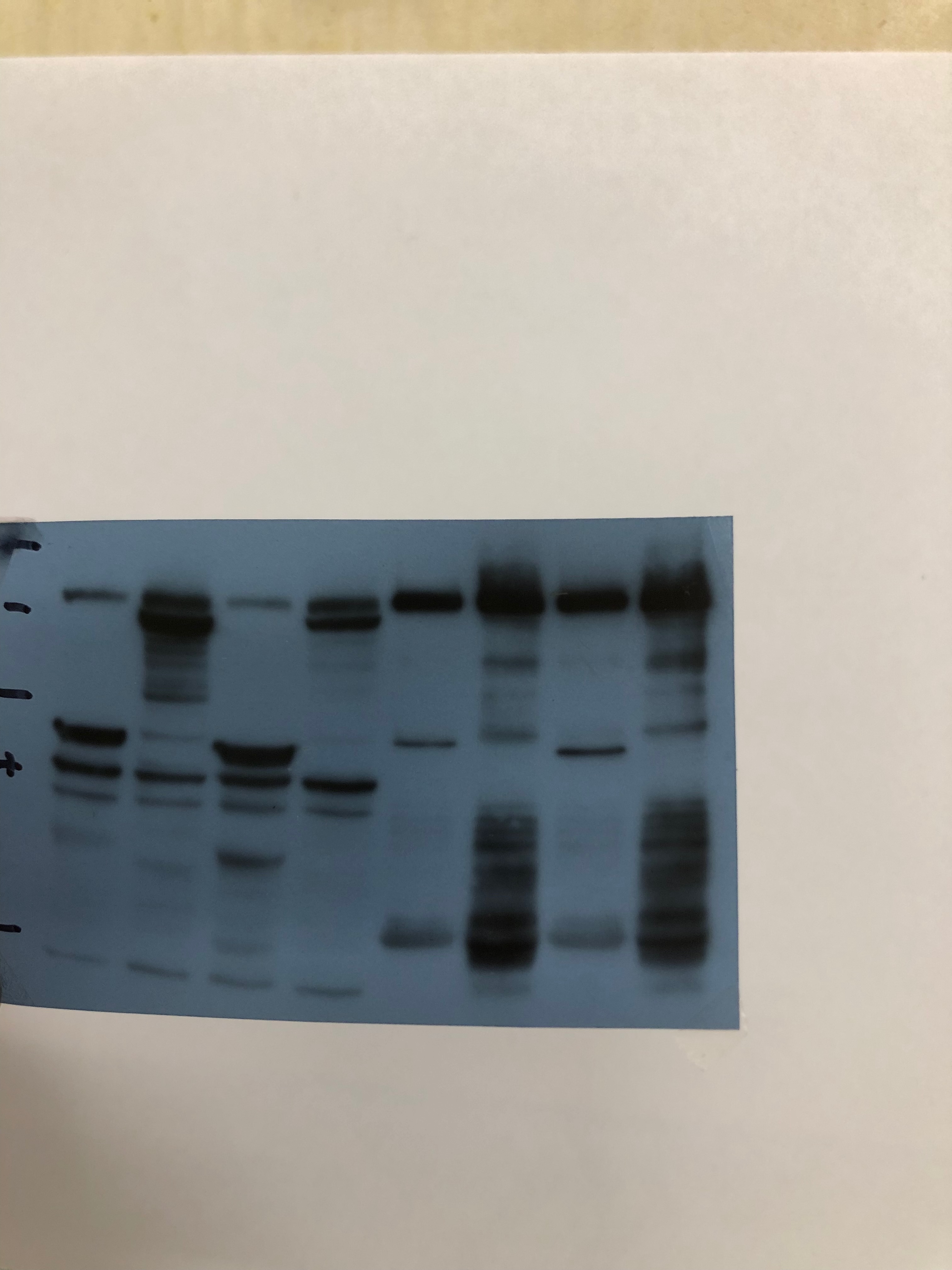

Supplement: Figure 4—source data 3. [file elife-87434-fig4-data3.zip › Figure4F.jpeg]

|               |   |   |   |   |   |   |   |   |
|---------------|---|---|---|---|---|---|---|---|
| Flag-Styxl2   | + | + | + | + | + | + | + | + |
| HA-Myh9-head  | + | - | - | - | + | - | - | - |
| HA-Myh9-tail  | - | + | - | - | - | + | - | - |
| HA-Myh10-head | - | - | + | - | - | - | + | - |
| HA-Myh10-tail | - | - | - | + | - | - | - | + |

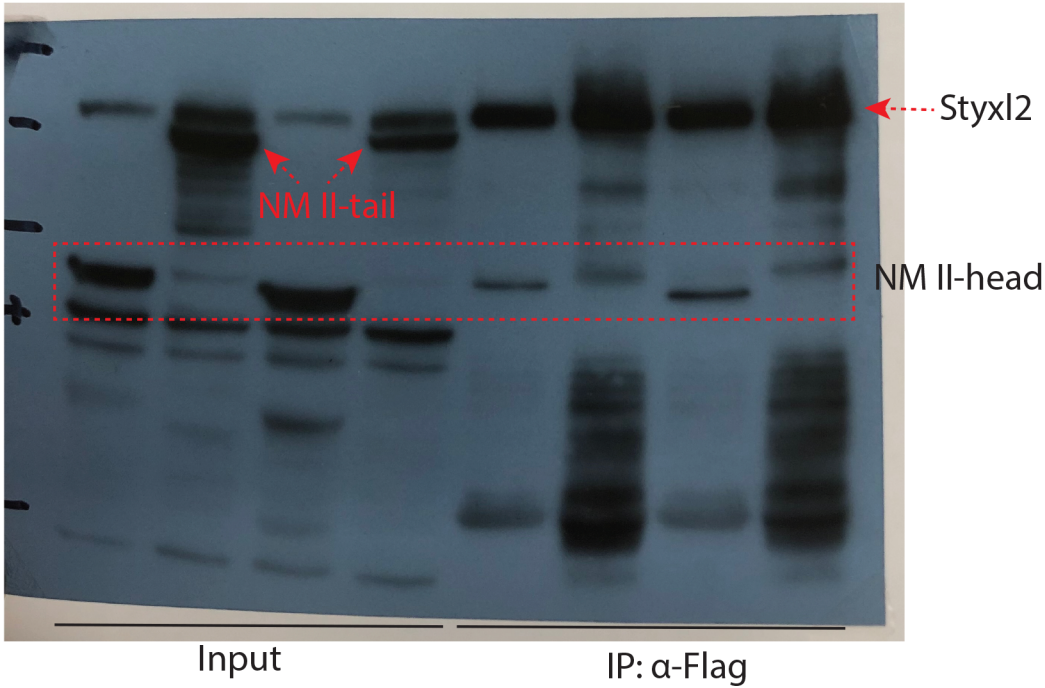

Supplement: Figure 4—source data 4. [file elife-87434-fig4-data4.zip › Figure4F.pdf]

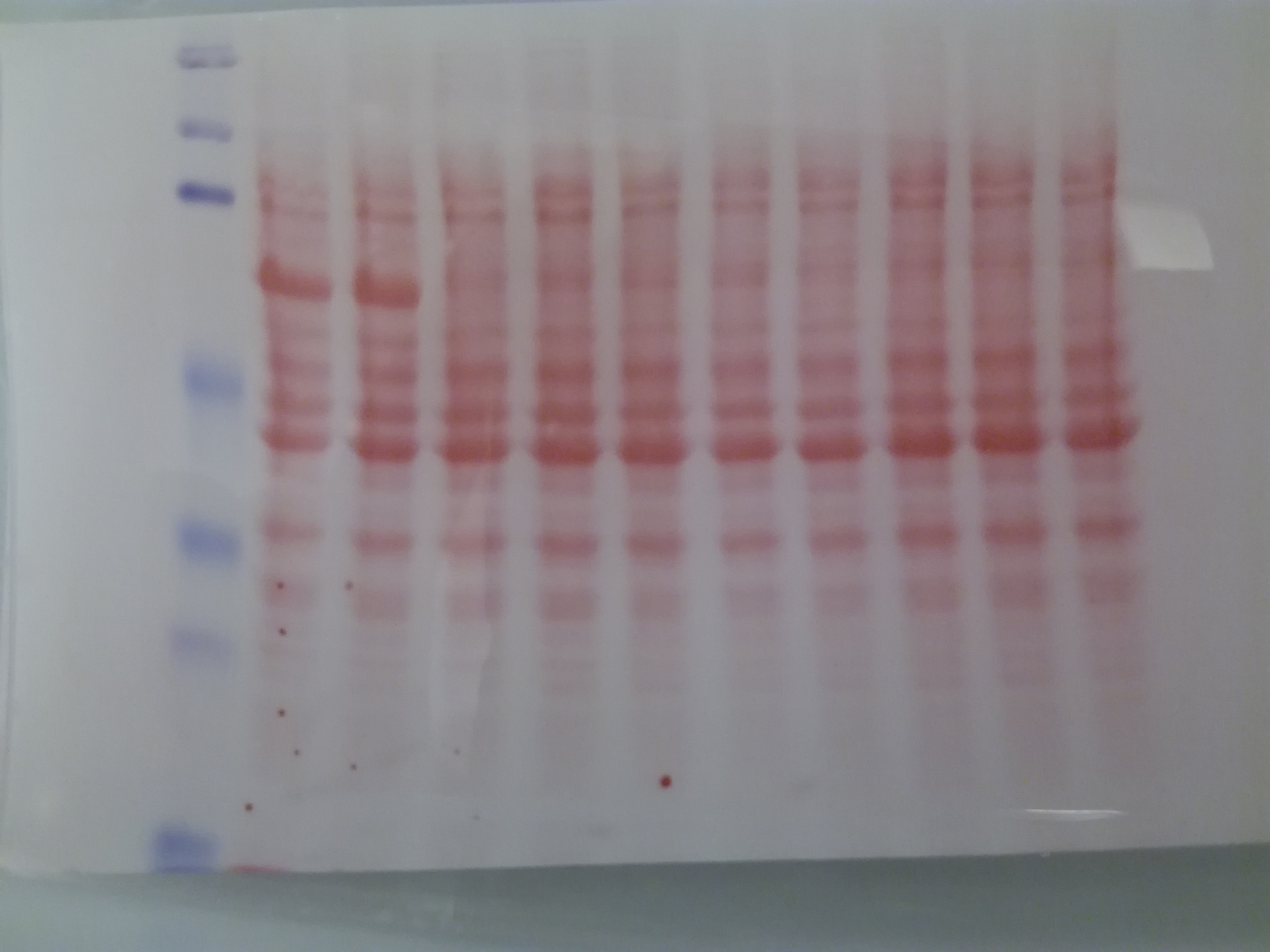

Supplement: Figure 4—figure supplement 1—source data 1. [file elife-87434-fig4-figsupp1-data1.zip › Figure4S1A/Figure4S1A-3.jpg]

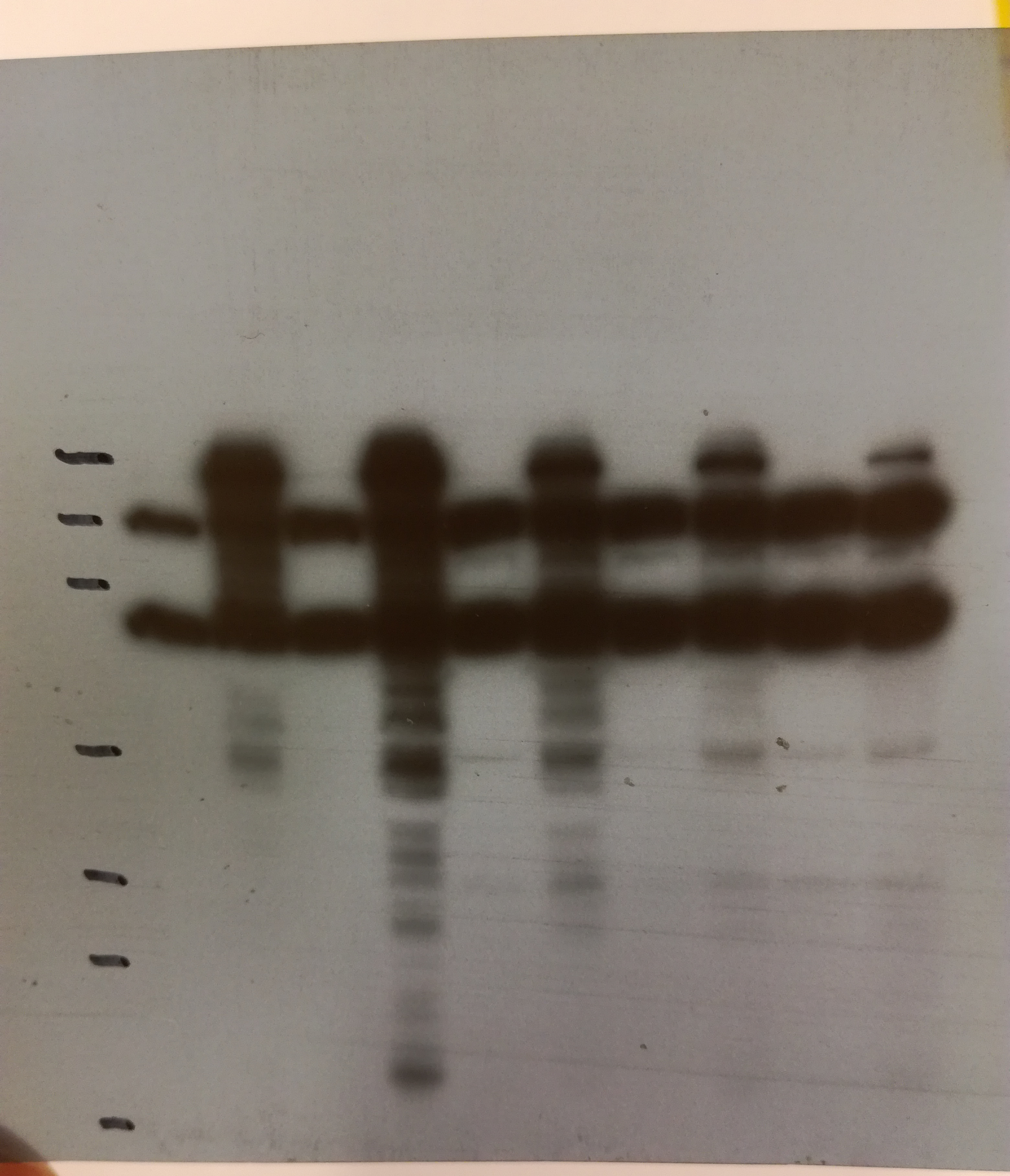

Supplement: Figure 4—figure supplement 1—source data 1. [file elife-87434-fig4-figsupp1-data1.zip › Figure4S1A/Figure4S1A-1.jpg]

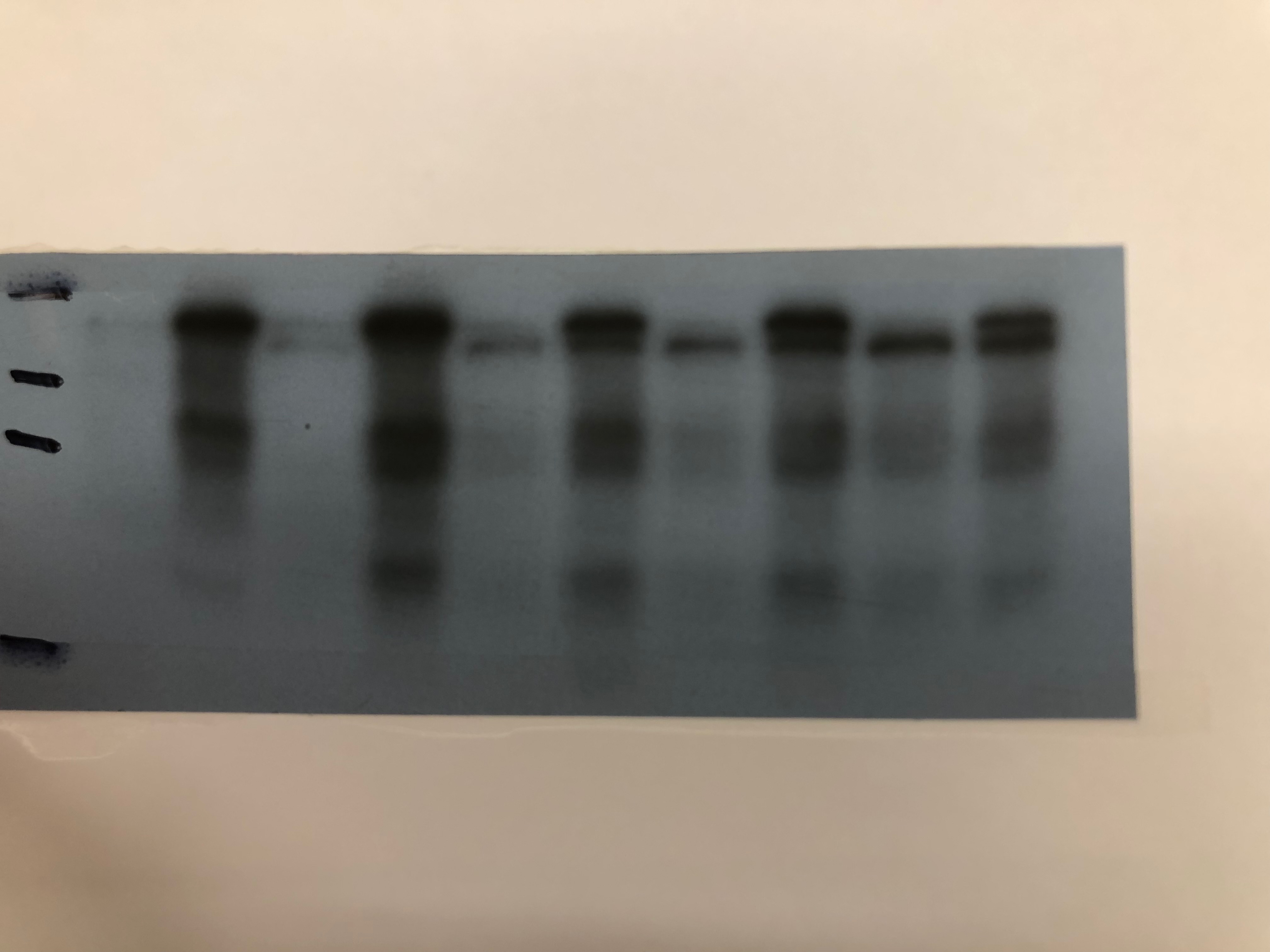

Supplement: Figure 4—figure supplement 1—source data 1. [file elife-87434-fig4-figsupp1-data1.zip › Figure4S1A/Figure4S1A-2.jpeg]

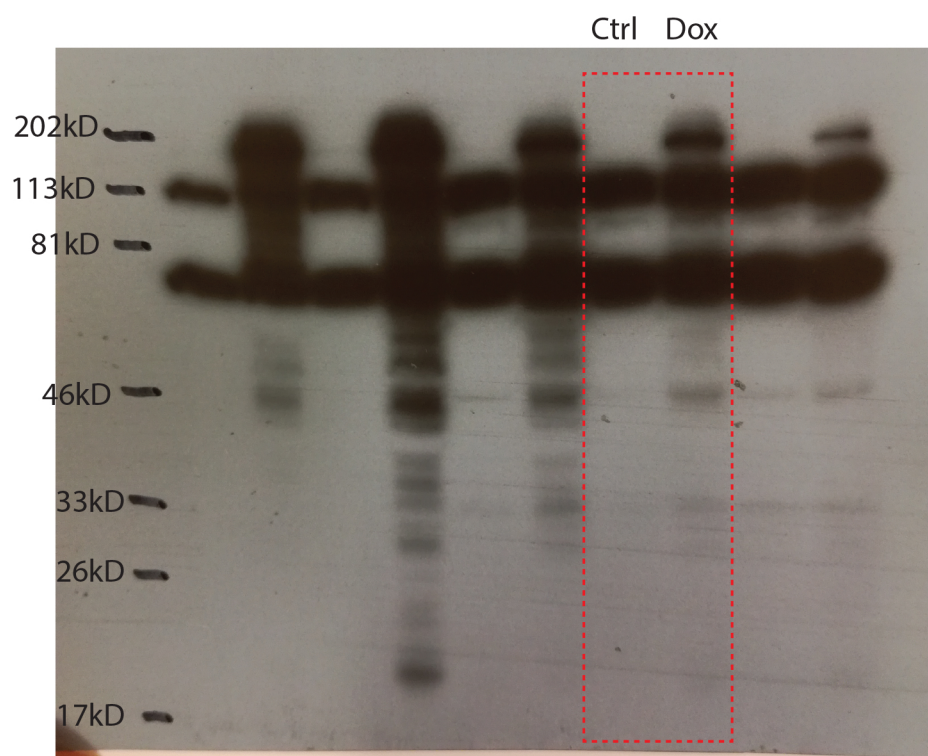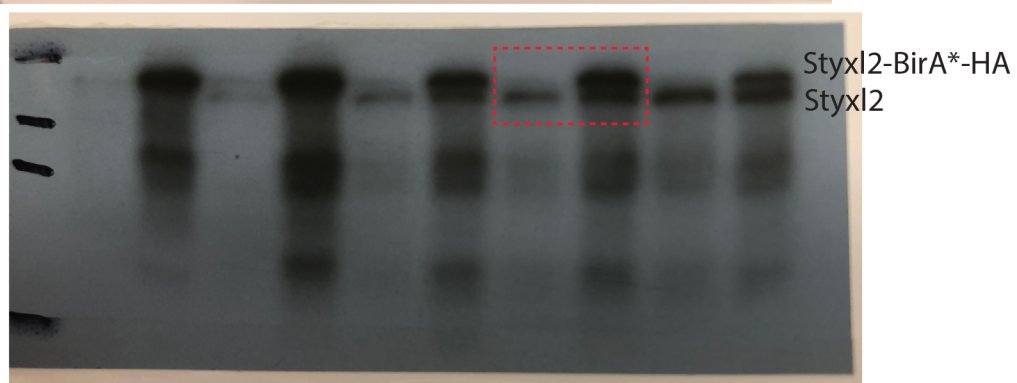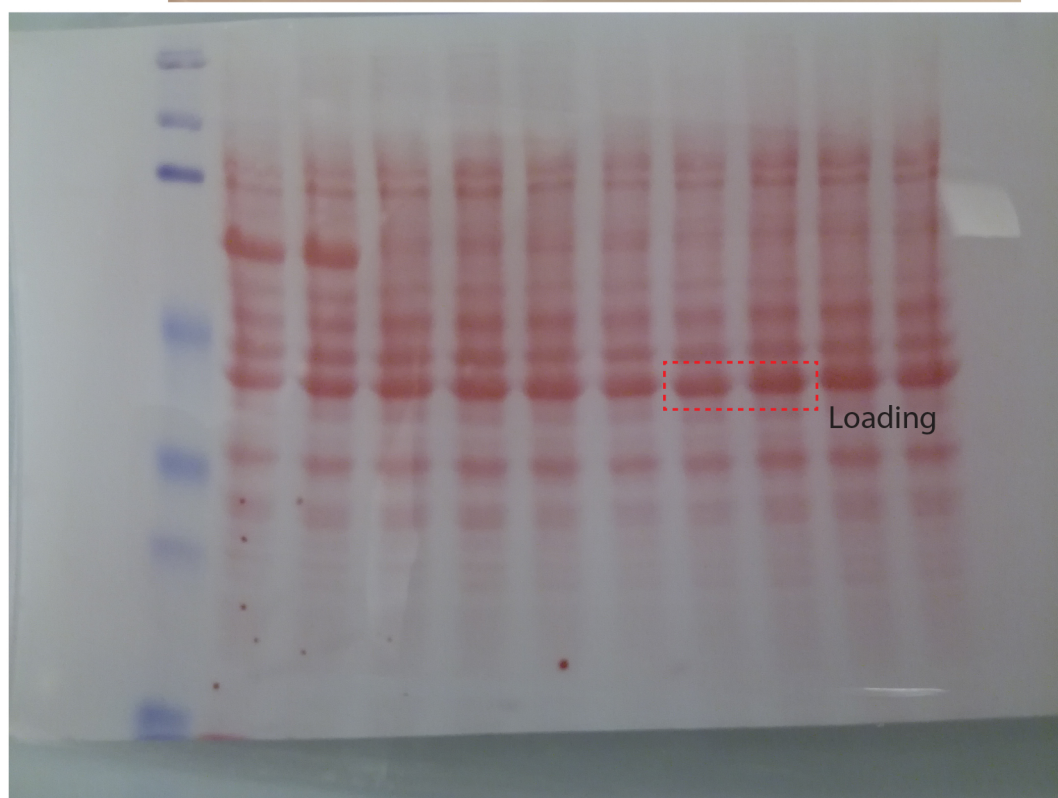

Supplement: Figure 4—figure supplement 1—source data 2. [file elife-87434-fig4-figsupp1-data2.zip › Figure4S1A.pdf]

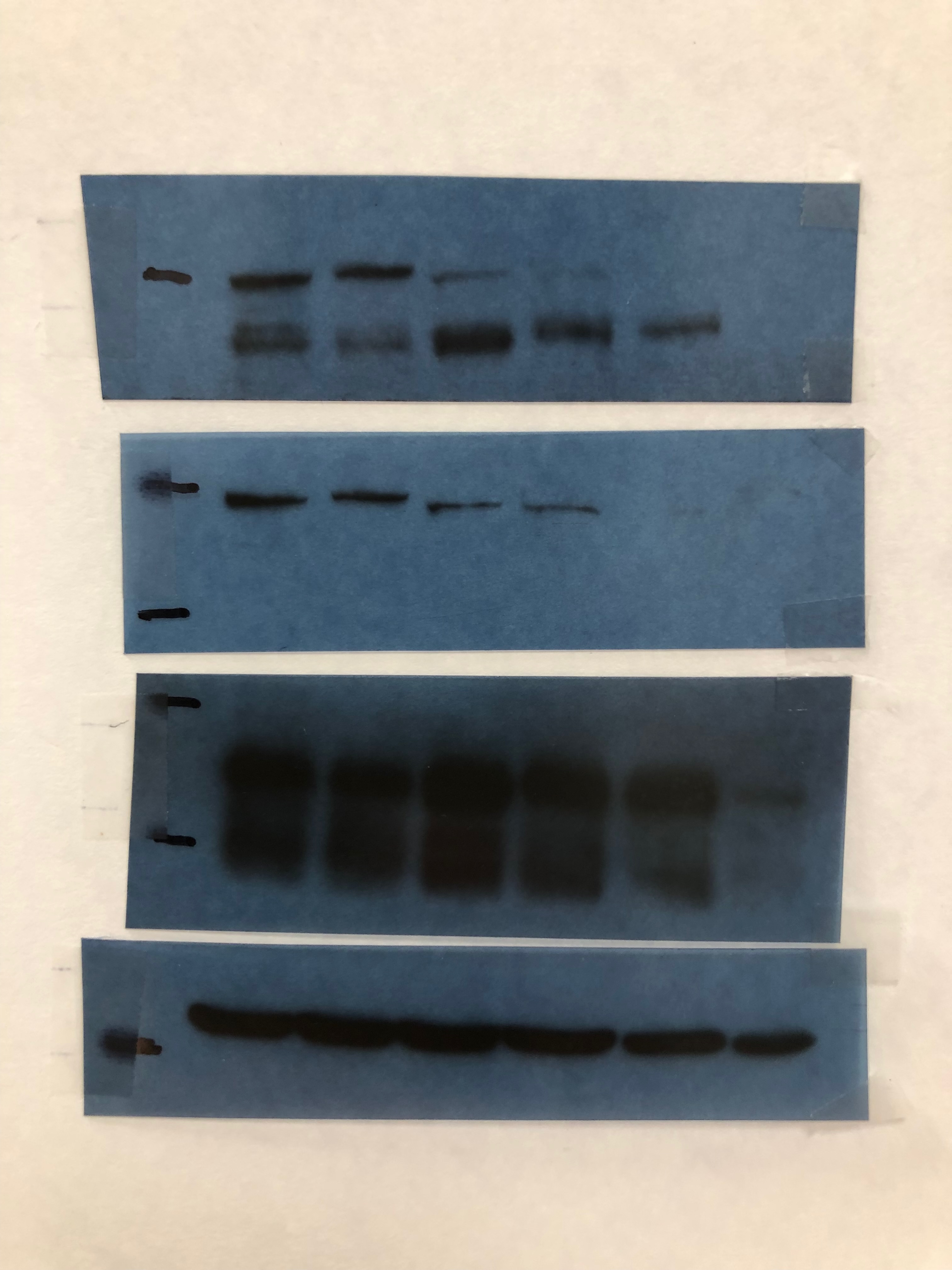

Supplement: Figure 5—source data 1. [file elife-87434-fig5-data1.zip › Figure5A.jpeg]

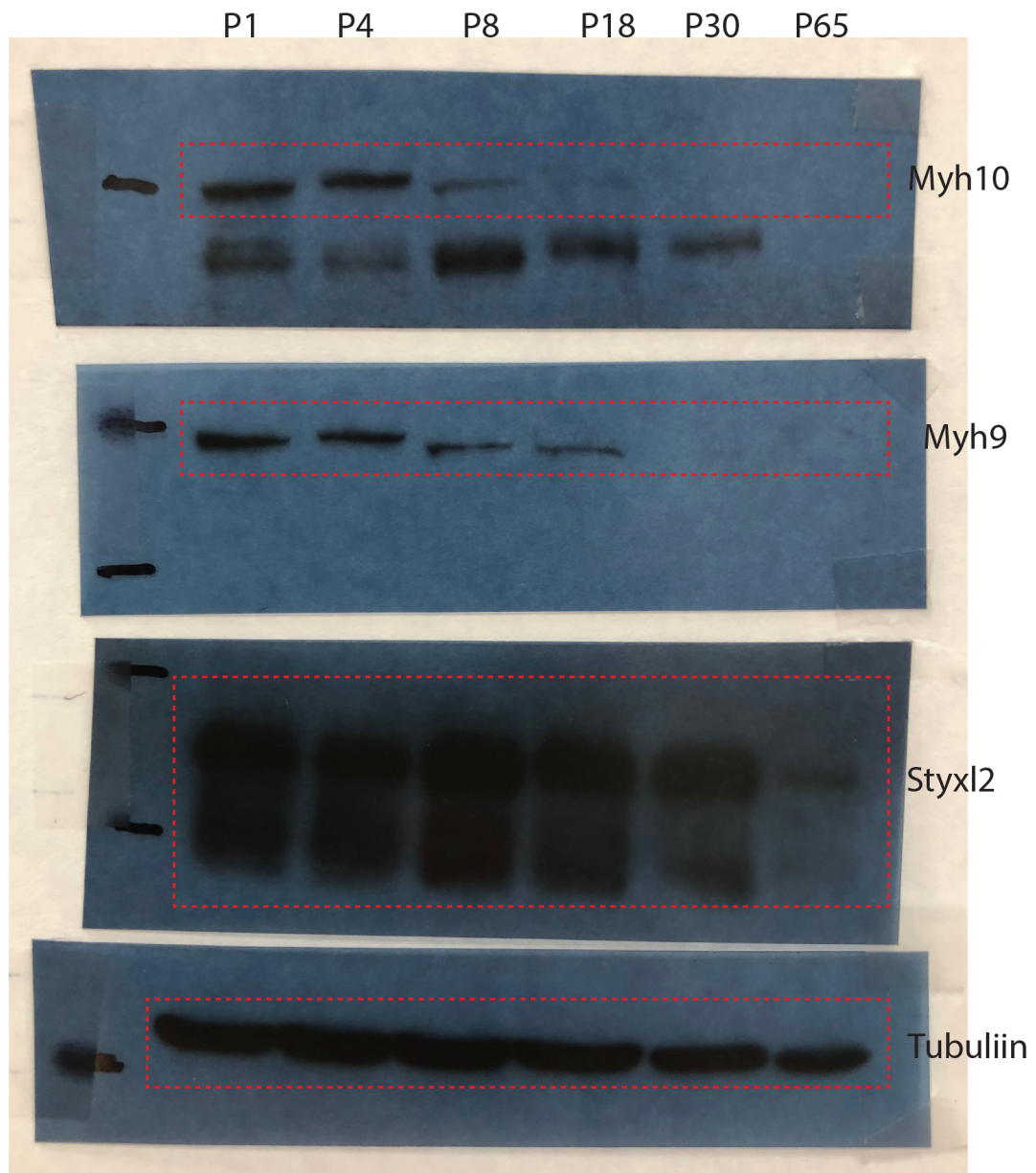

Supplement: Figure 5—source data 2. [file elife-87434-fig5-data2.zip › Figure5A.pdf]

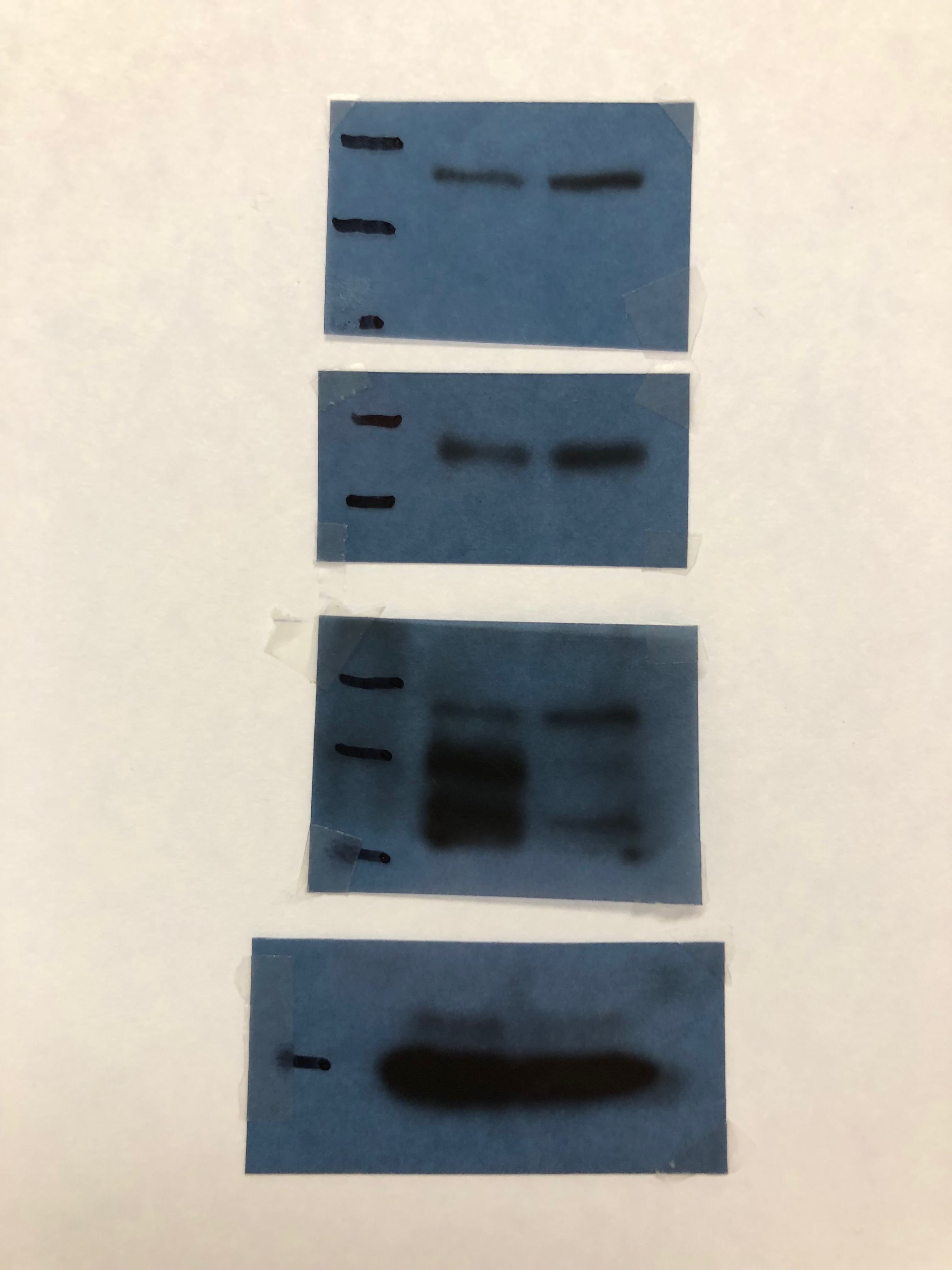

Supplement: Figure 5—source data 3. [file elife-87434-fig5-data3.zip › Figure5B.jpeg]

Ctrl CKO

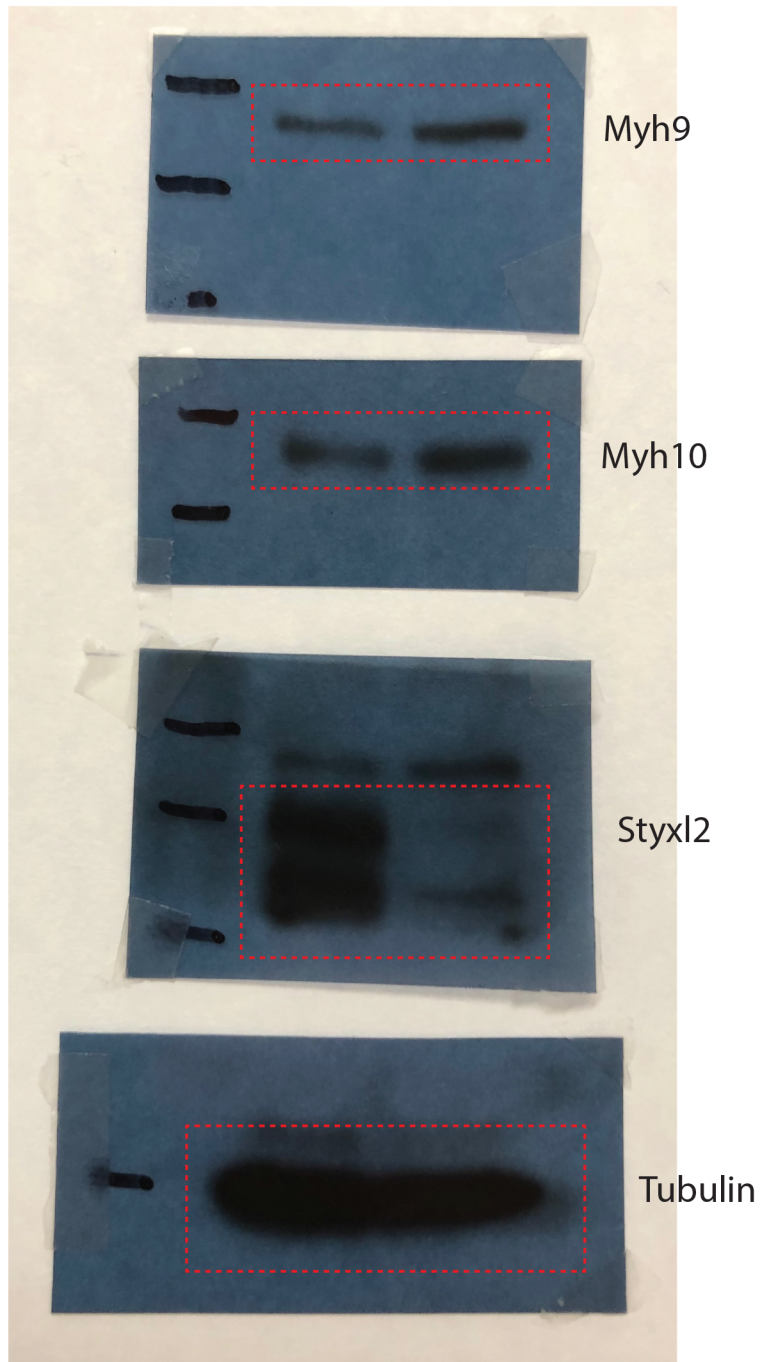

Supplement: Figure 5—source data 4. [file elife-87434-fig5-data4.zip › Figure5B.pdf]

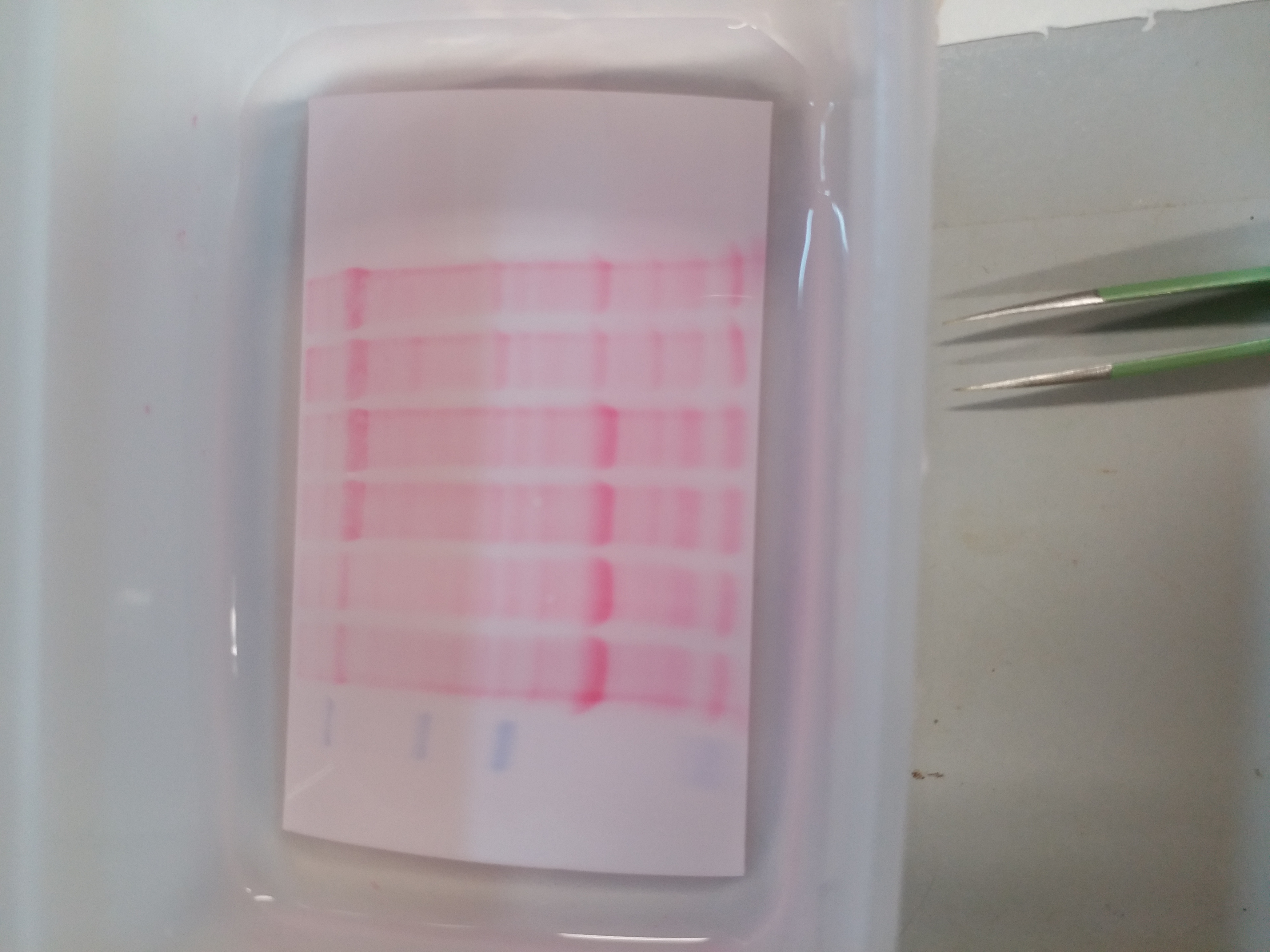

Supplement: Figure 5—source data 5. [file elife-87434-fig5-data5.zip › Figure5D/Figure5D-2.jpg]

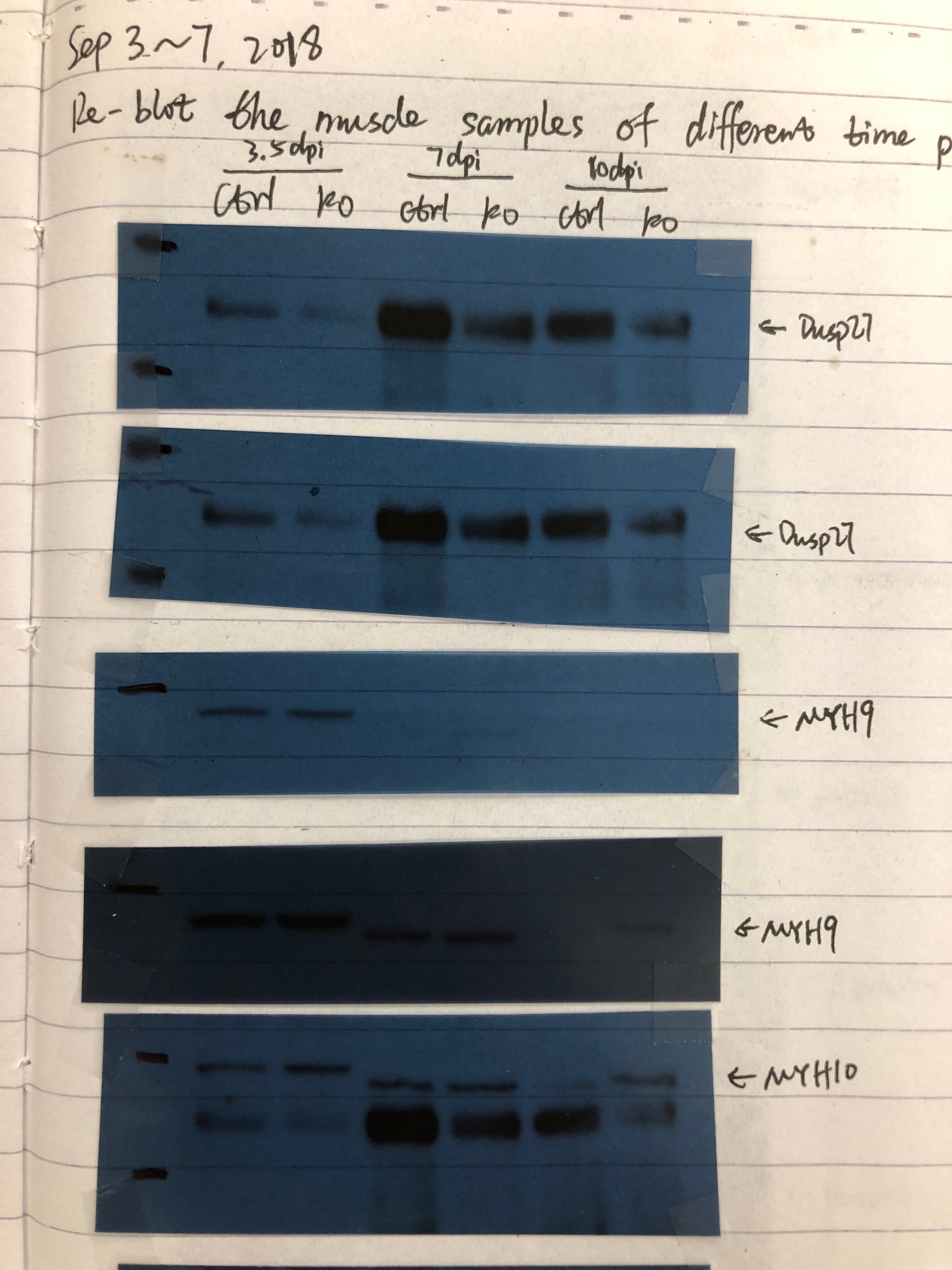

Supplement: Figure 5—source data 5. [file elife-87434-fig5-data5.zip › Figure5D/Figure5D-1.jpeg]

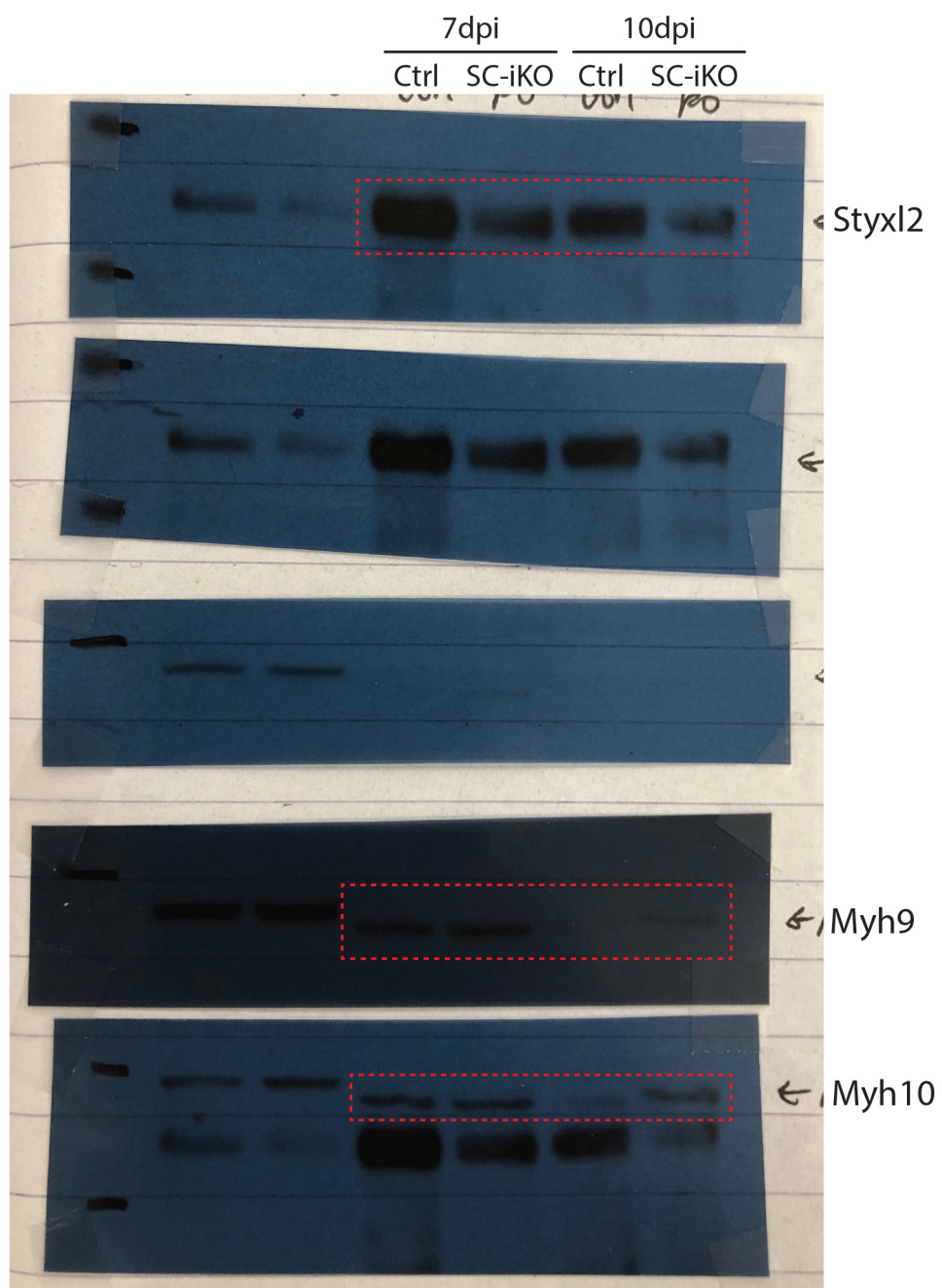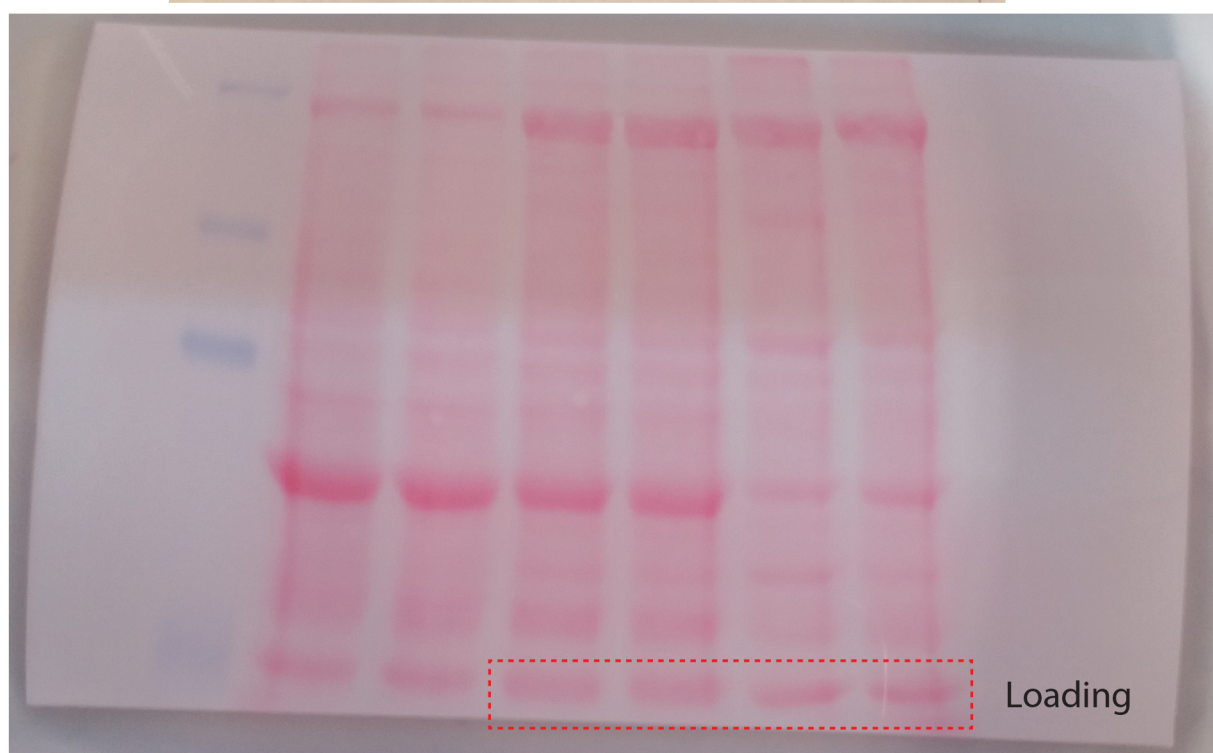

Supplement: Figure 5—source data 6. [file elife-87434-fig5-data6.zip › Figure5D.pdf]

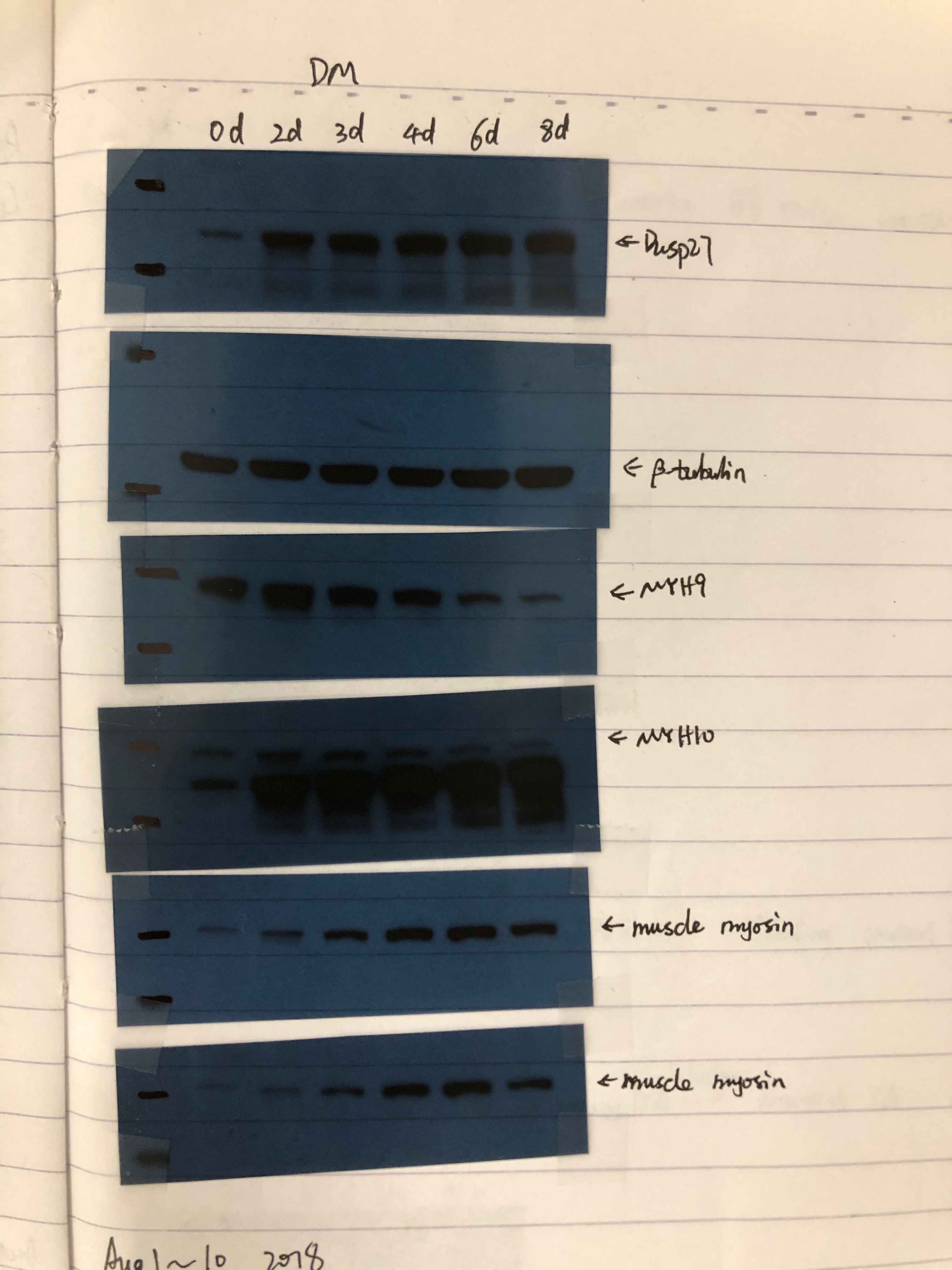

Supplement: Figure 5—figure supplement 1—source data 1. [file elife-87434-fig5-figsupp1-data1.zip › Figure5S1A.jpeg]

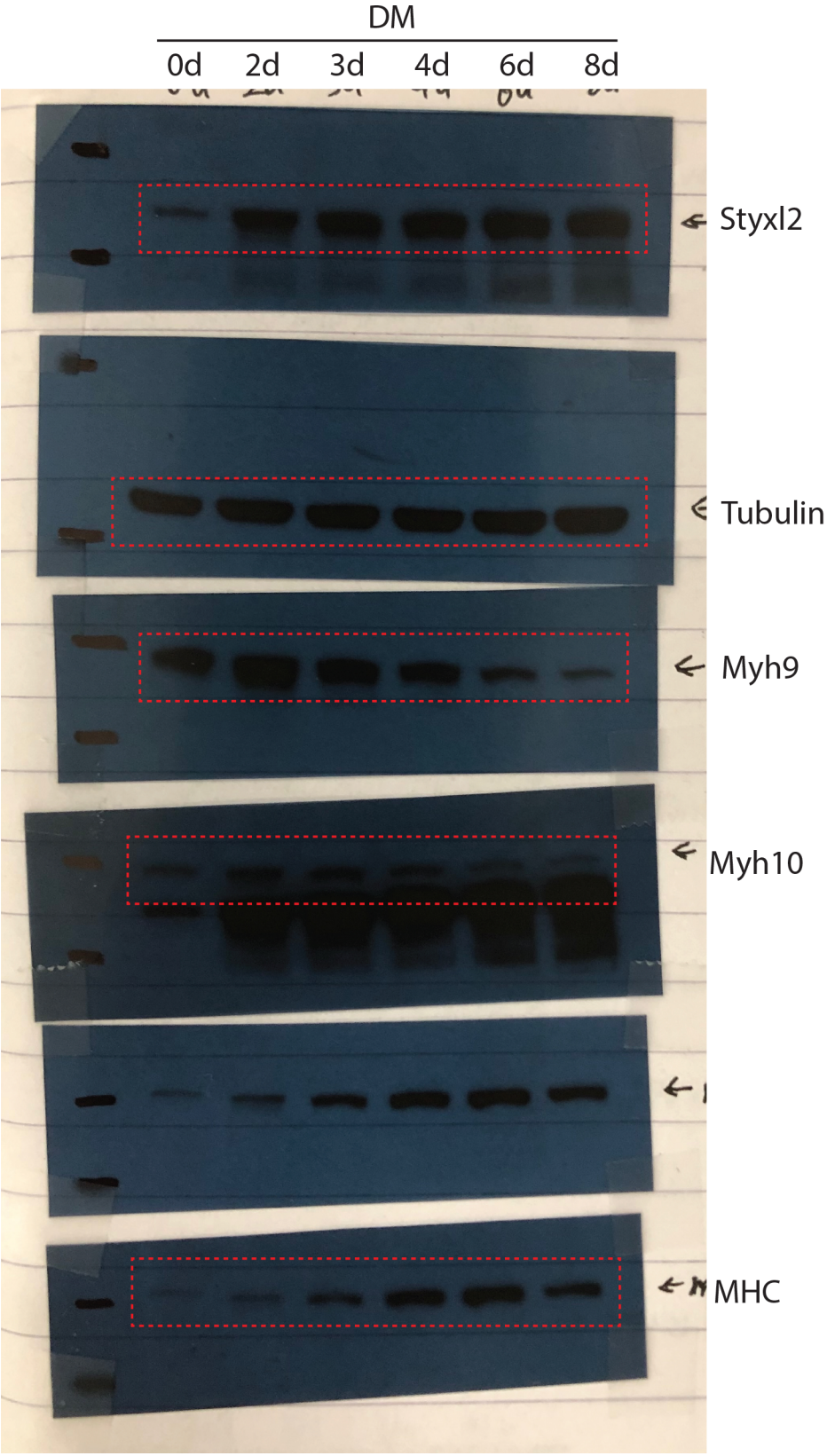

Supplement: Figure 5—figure supplement 1—source data 2. [file elife-87434-fig5-figsupp1-data2.zip › Figure5S1A.pdf]

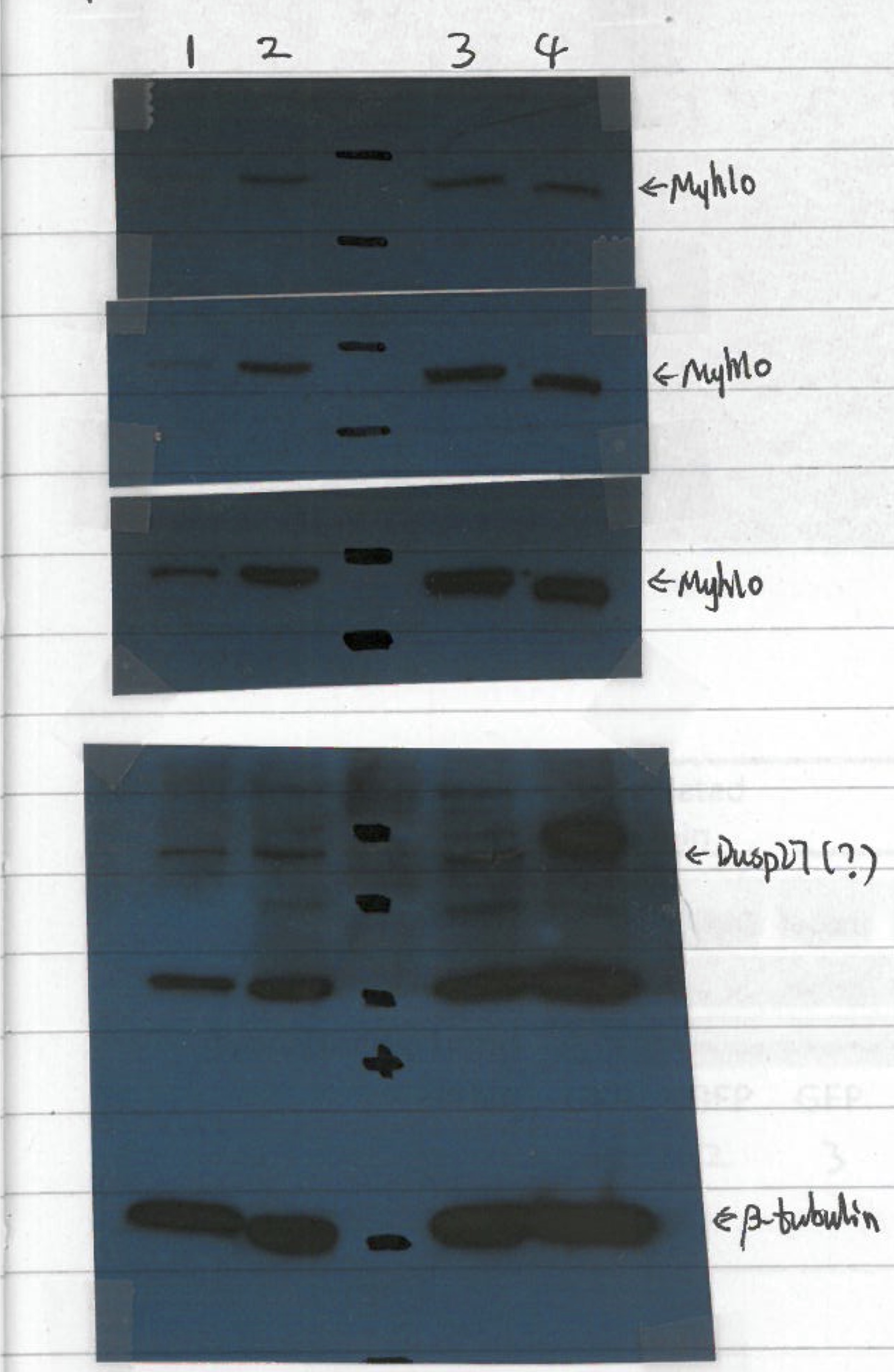

Supplement: Figure 5—figure supplement 1—source data 3. [file elife-87434-fig5-figsupp1-data3.zip › Figure5S1B.jpg]

MO  
Myh10 Ctrl

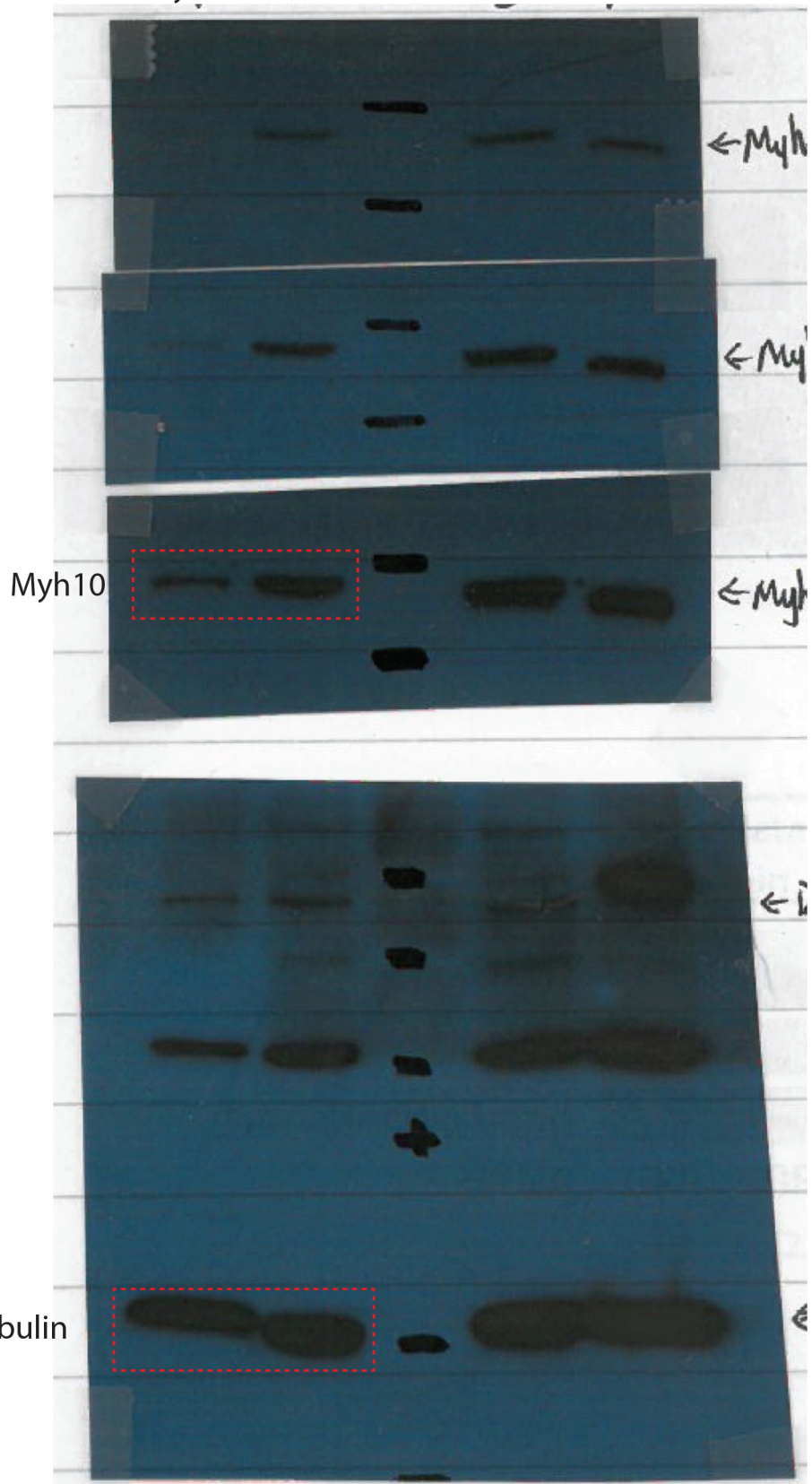

Supplement: Figure 5—figure supplement 1—source data 4. [file elife-87434-fig5-figsupp1-data4.zip › Figure5S1B.pdf]

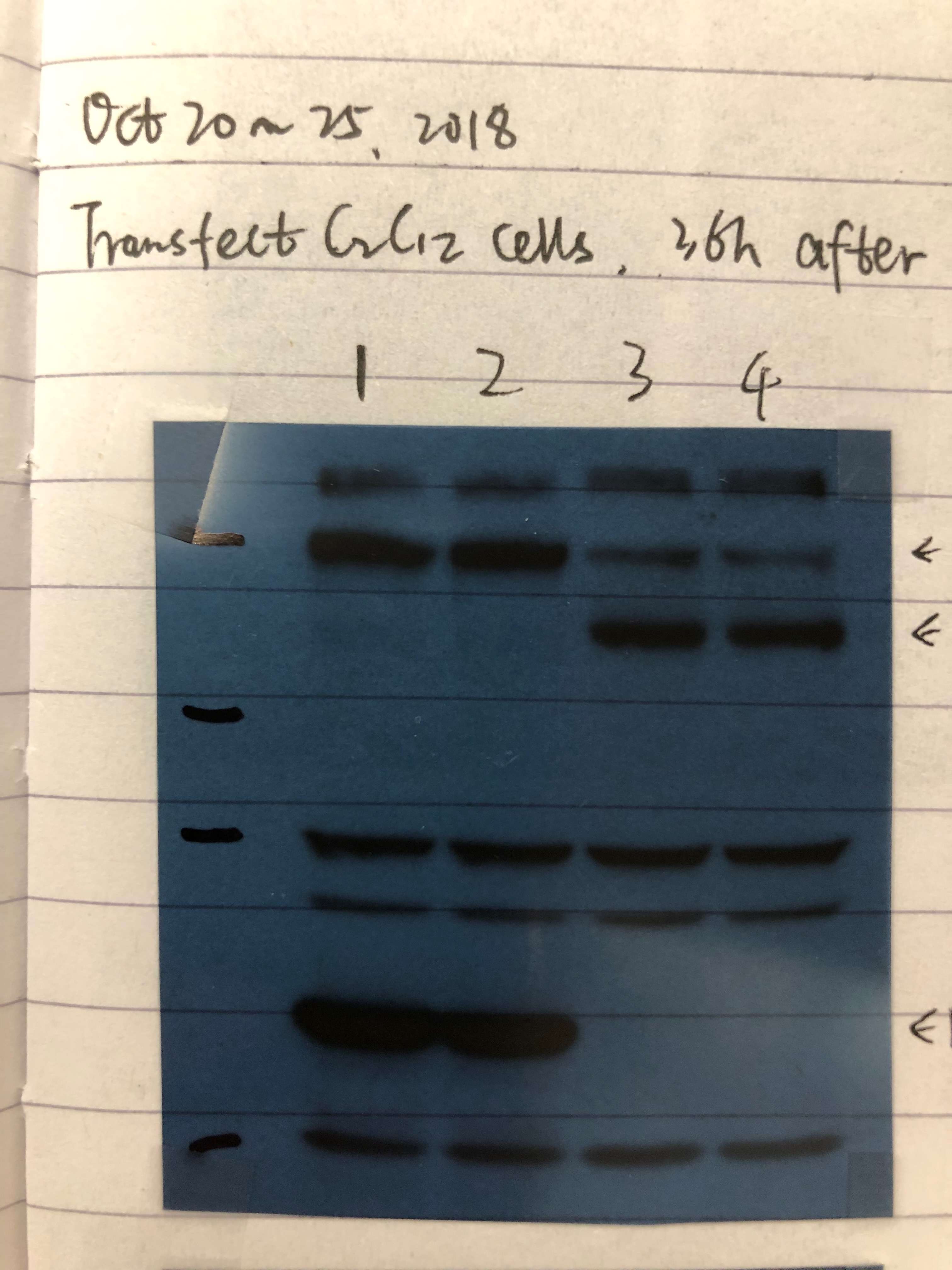

Supplement: Figure 6—source data 1. [file elife-87434-fig6-data1.zip › Figure6A.jpeg]

OE: 

|           |             |
|-----------|-------------|
| HA-Myh9   |             |
| Flag-Mst1 | Flag-Styxl2 |

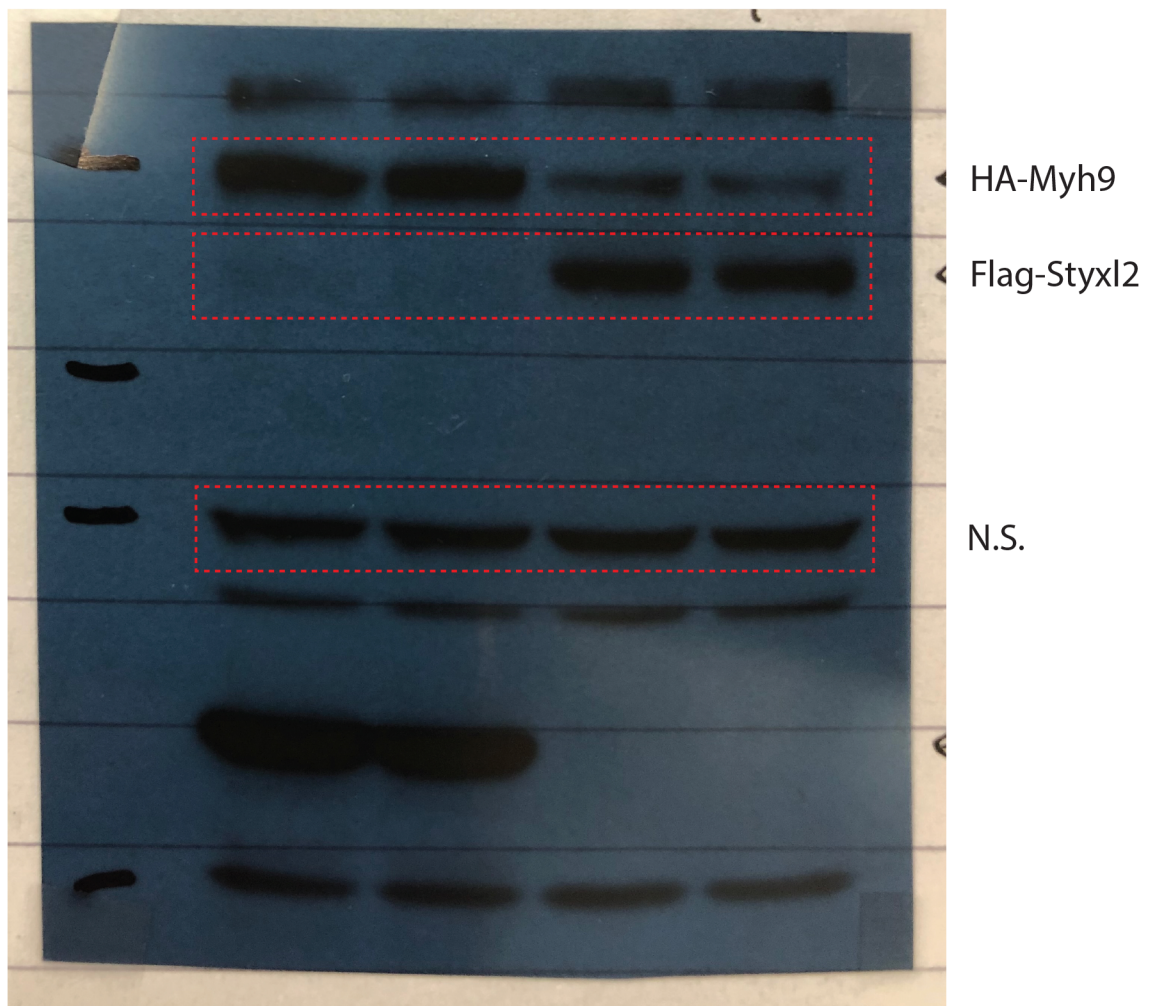

Supplement: Figure 6—source data 2. [file elife-87434-fig6-data2.zip › Figure6A.pdf]

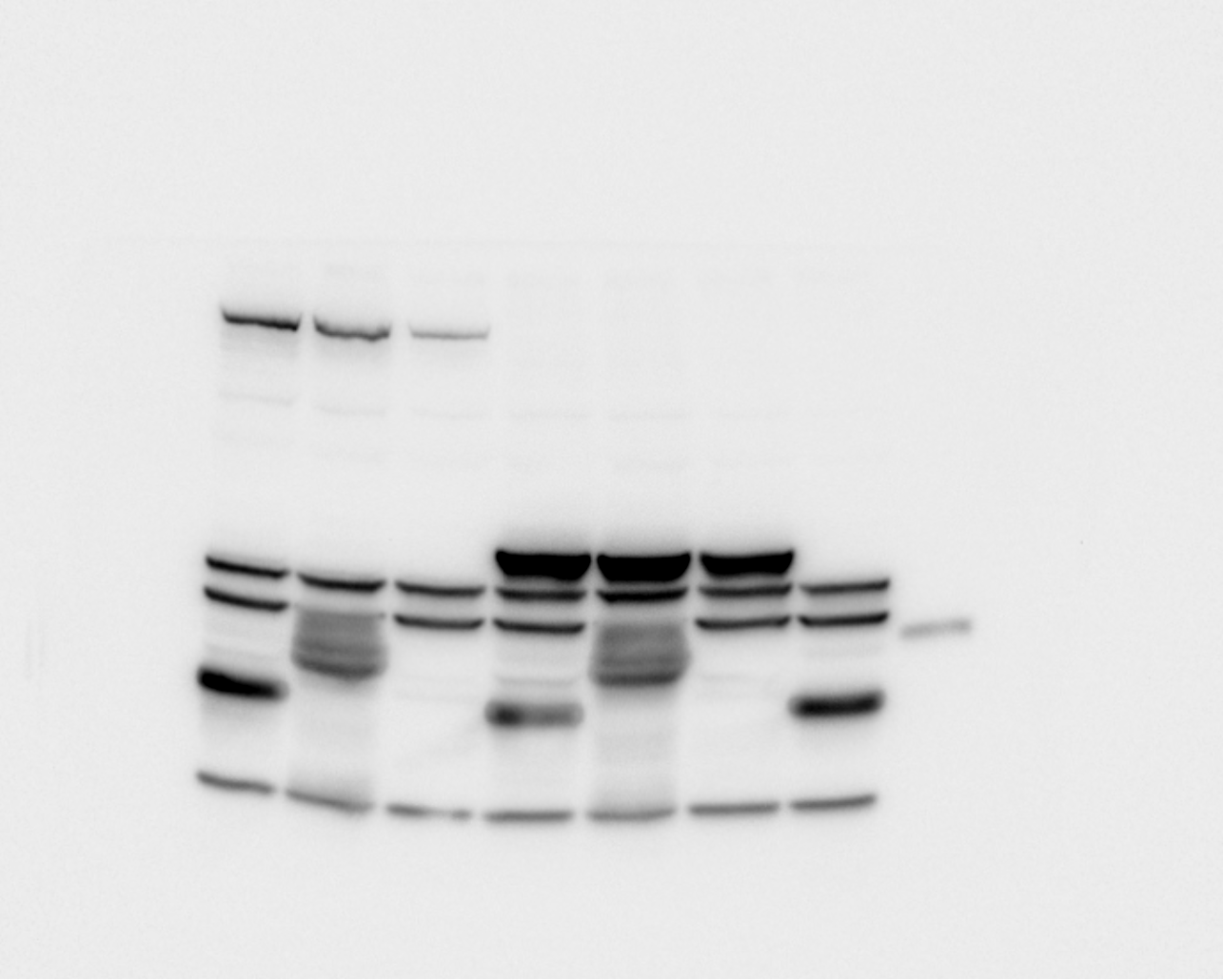

Supplement: Figure 6—source data 3. [file elife-87434-fig6-data3.zip › Figure6B/Figure6B-1.tif]

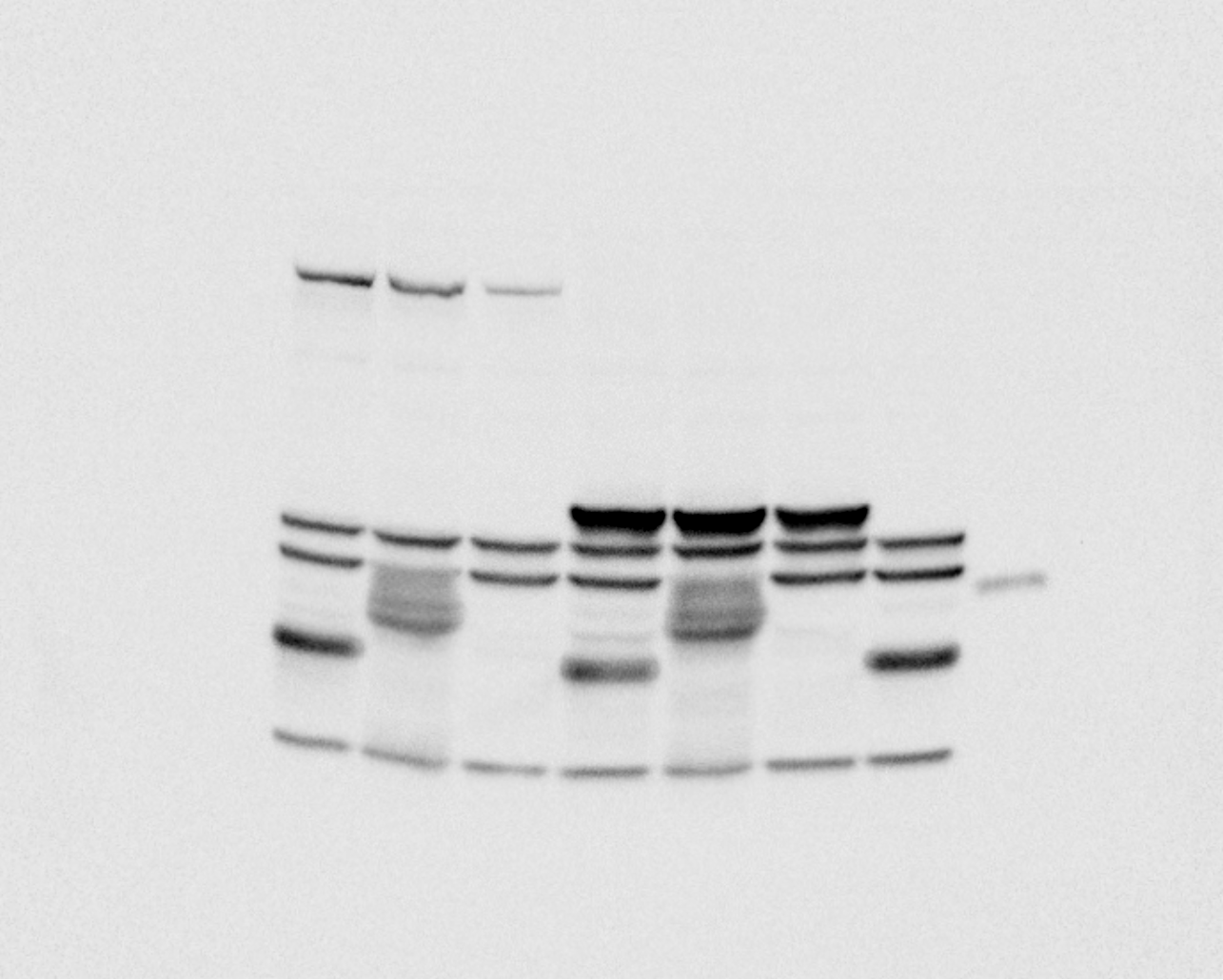

Supplement: Figure 6—source data 3. [file elife-87434-fig6-data3.zip › Figure6B/Figure6B-3.tif]

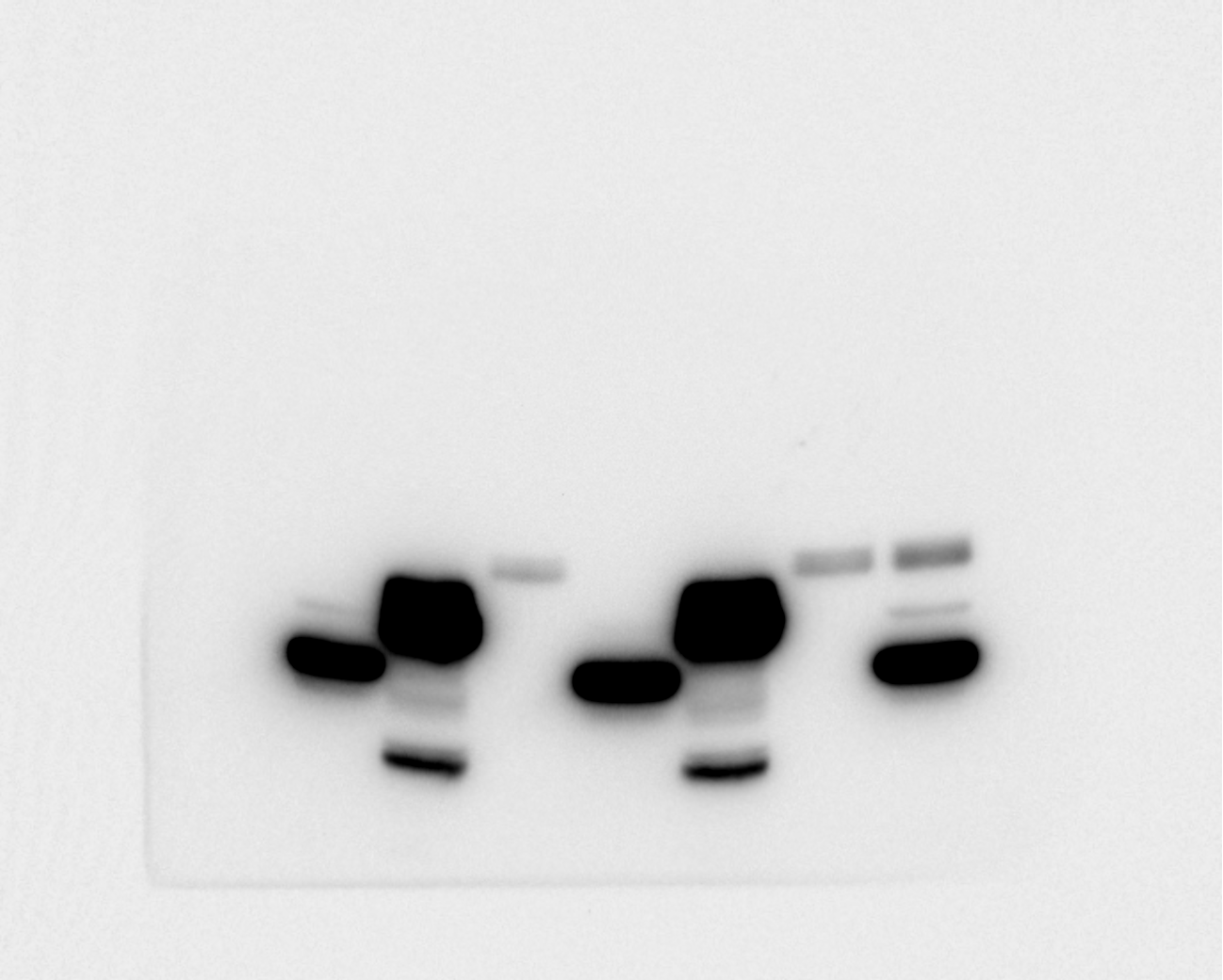

Supplement: Figure 6—source data 3. [file elife-87434-fig6-data3.zip › Figure6B/Figure6B-2.tif]

|                  |   |   |   |   |   |   |   |
|------------------|---|---|---|---|---|---|---|
| HA-Myh9          | + | + | + | - | - | - | - |
| HA-Myh9-head     | - | - | - | + | + | + | - |
| Flag-Styxl2ΔN509 | - | - | + | - | - | + | + |
| Flag-Styxl2N513  | - | + | - | - | + | - | - |
| Flag-Mst1        | + | - | - | + | - | - | + |

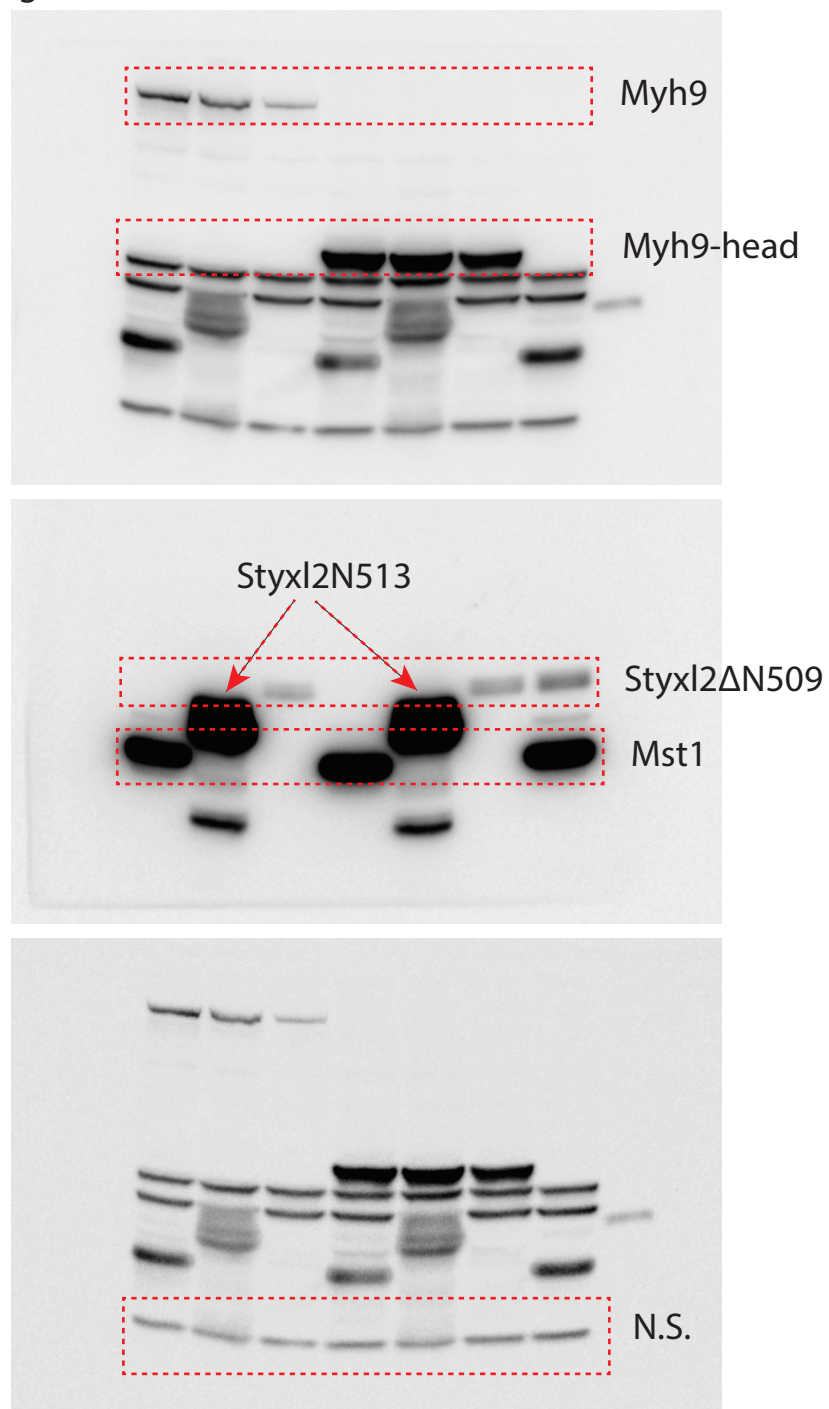

Supplement: Figure 6—source data 4. [file elife-87434-fig6-data4.zip › Figure6B.pdf]

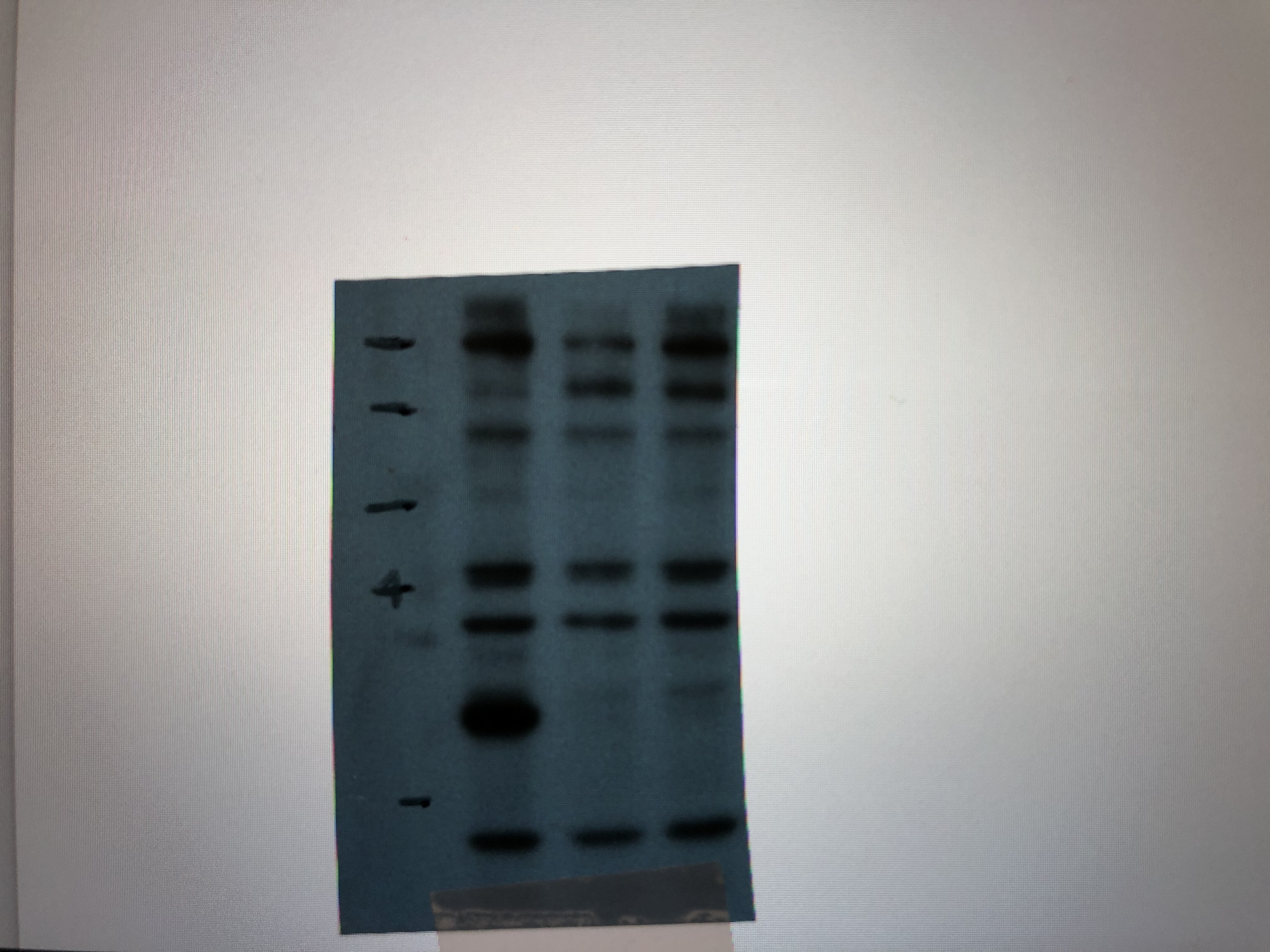

Supplement: Figure 7—source data 1. [file elife-87434-fig7-data1.zip › Figure7A.jpg]

OE: Myh9  
Mst1 Styx12

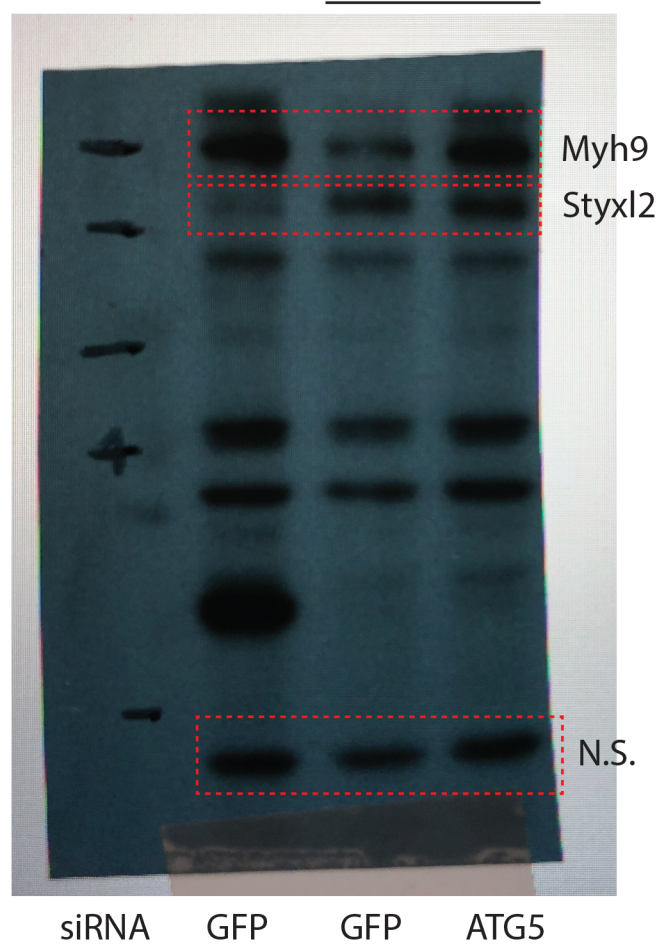

Supplement: Figure 7—source data 2. [file elife-87434-fig7-data2.zip › Figure7A.pdf]

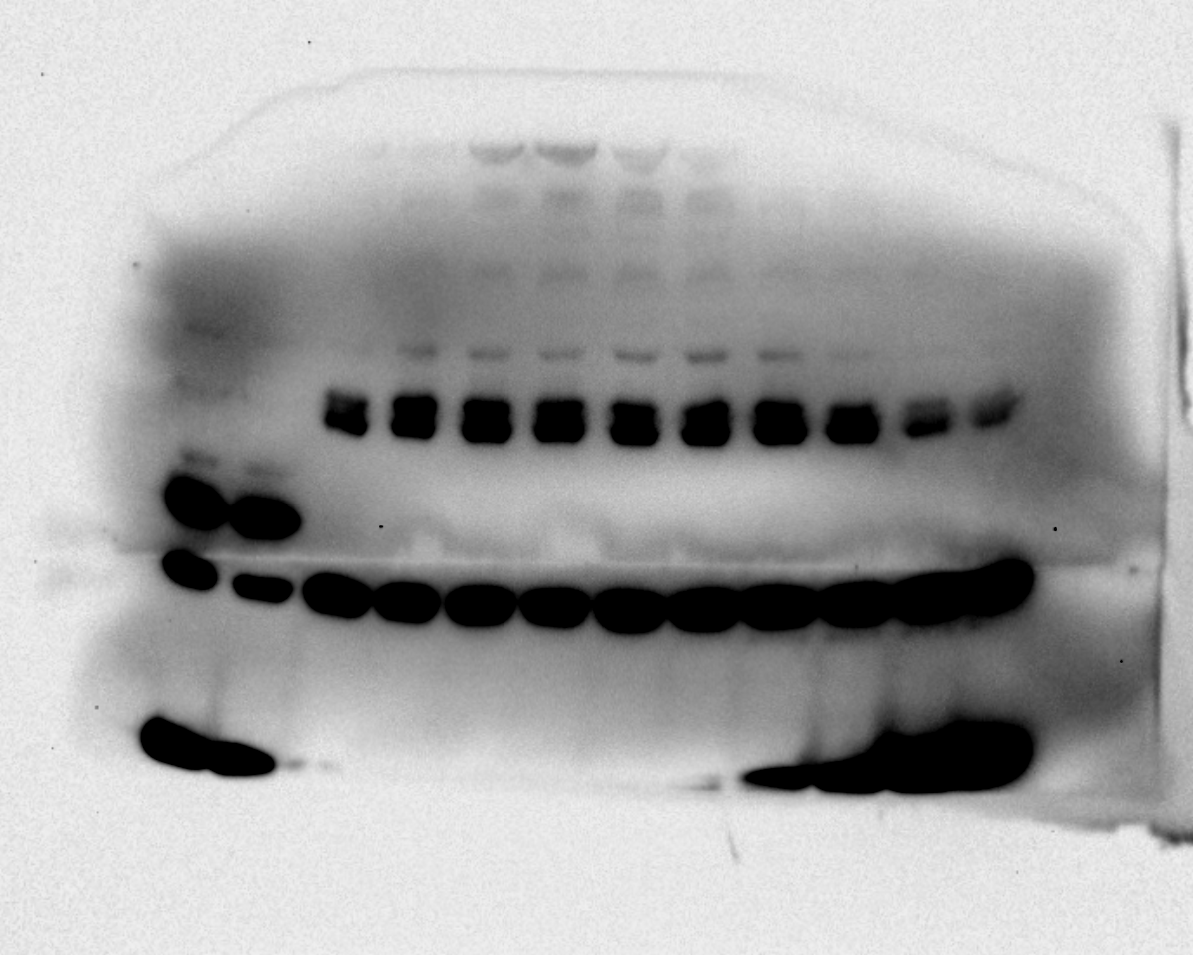

Supplement: Figure 7—source data 3. [file elife-87434-fig7-data3.zip › Figure7B/Figure7B-2.tif]

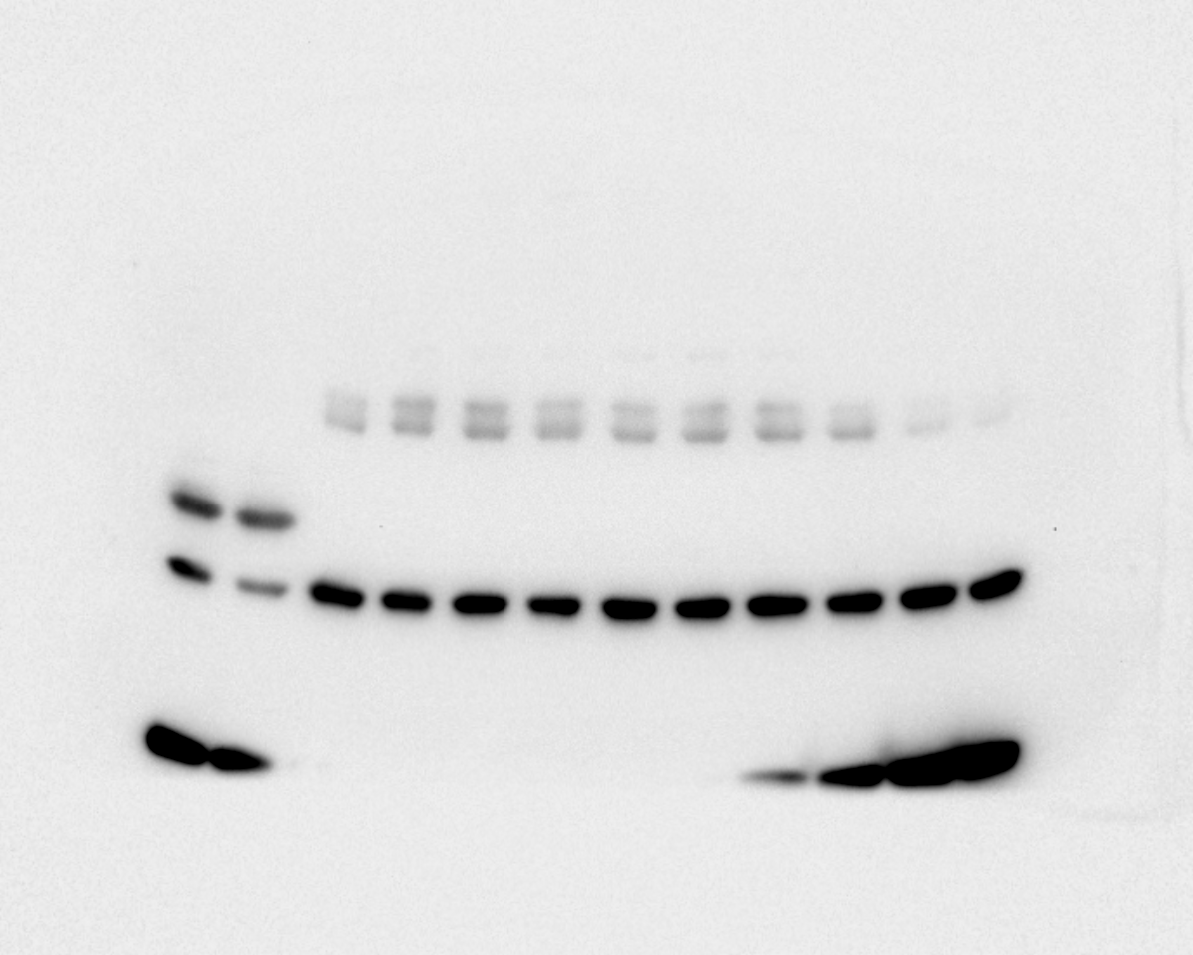

Supplement: Figure 7—source data 3. [file elife-87434-fig7-data3.zip › Figure7B/Figure7B-3.tif]

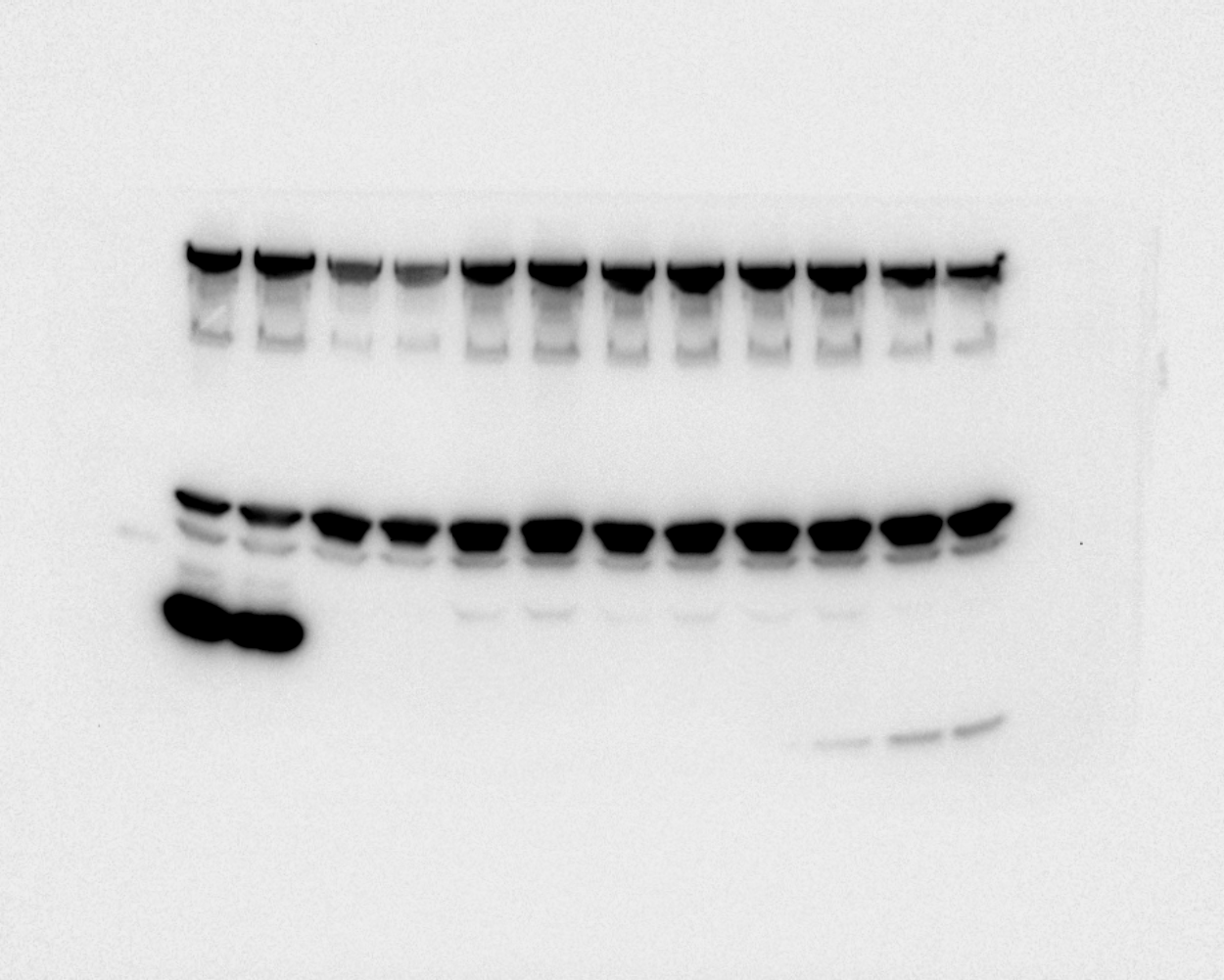

Supplement: Figure 7—source data 3. [file elife-87434-fig7-data3.zip › Figure7B/Figure7B-1.tif]

OE: Myh9  
Mst1 Styxl2ΔN509

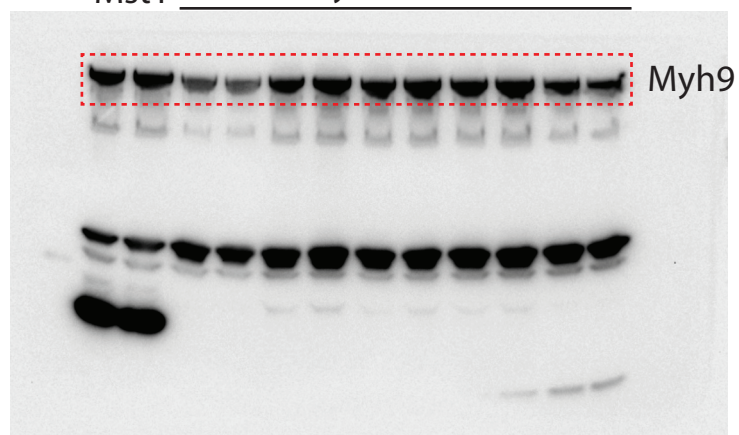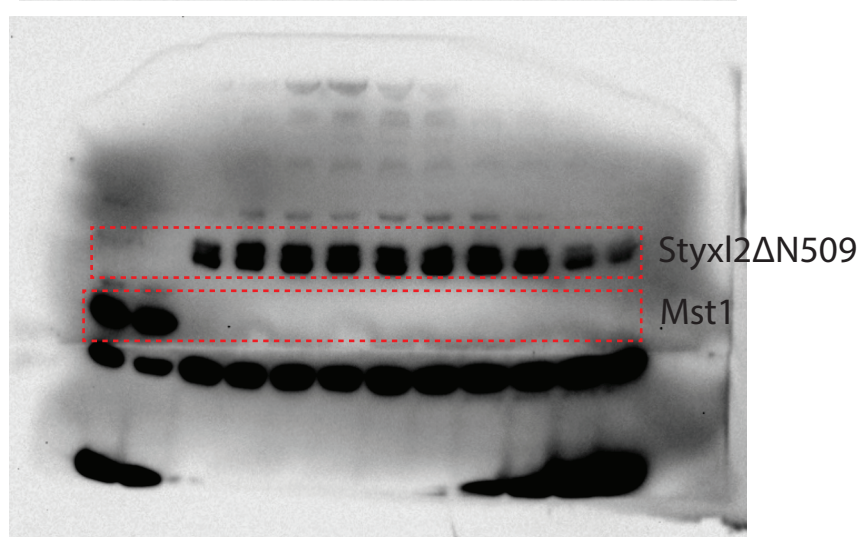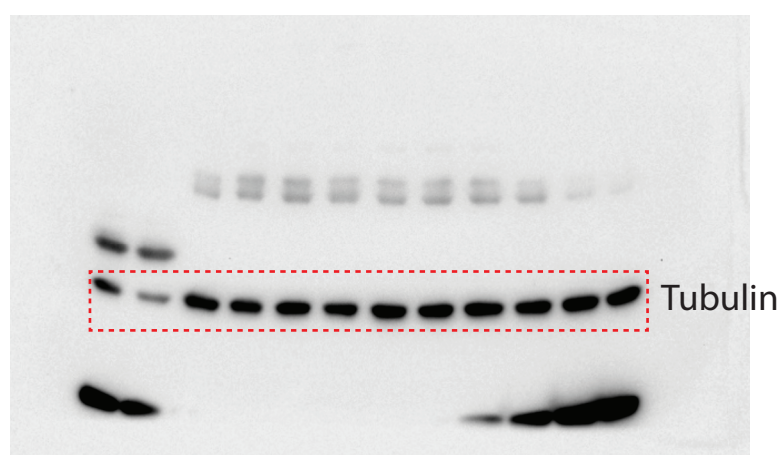

Control LY294002 Chloroquine BafA1 NH<sub>4</sub>Cl

Supplement: Figure 7—source data 4. [file elife-87434-fig7-data4.zip › Figure7B.pdf]

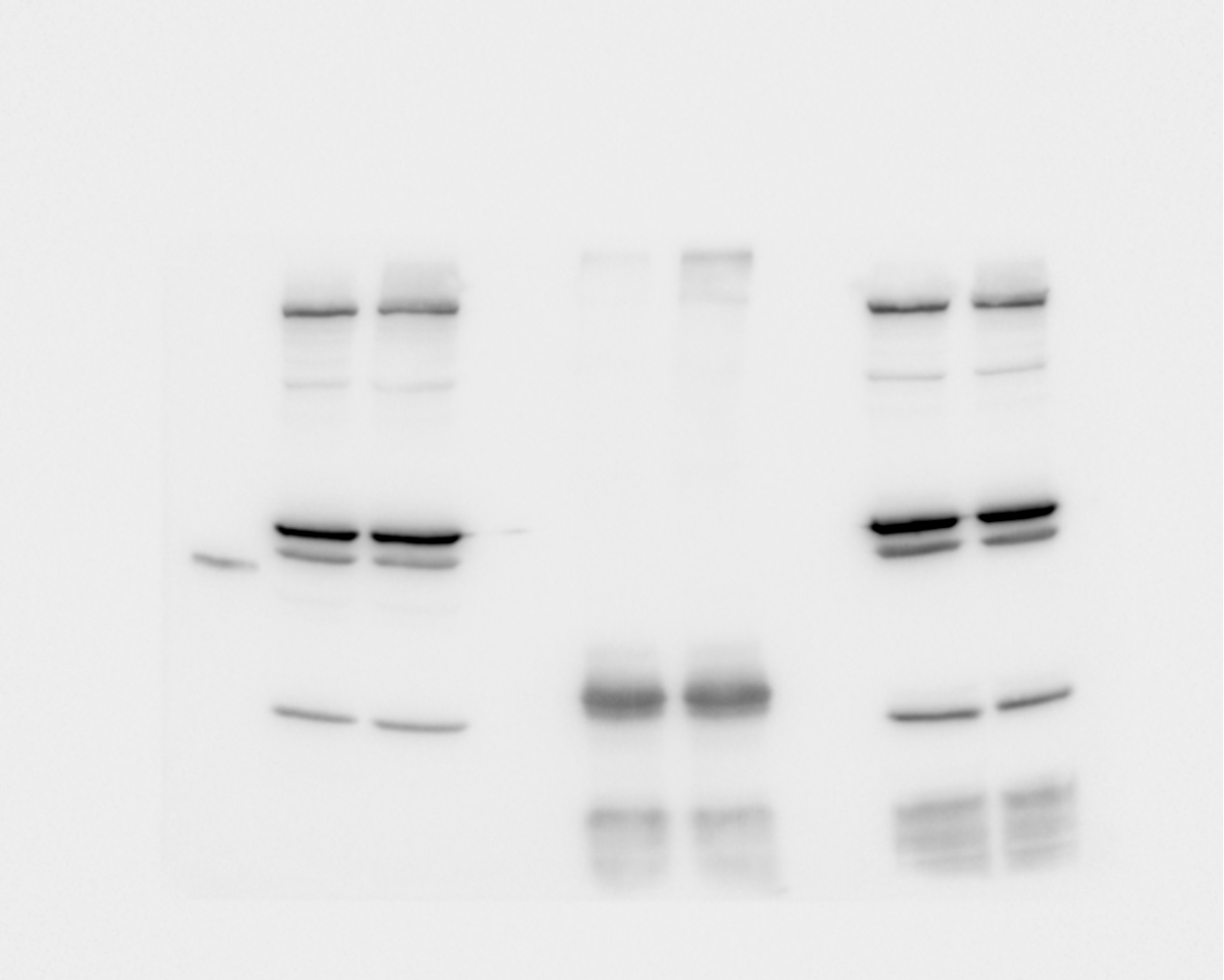

Supplement: Figure 7—source data 5. [file elife-87434-fig7-data5.zip › Figure7C/Figure7C-1.tif]

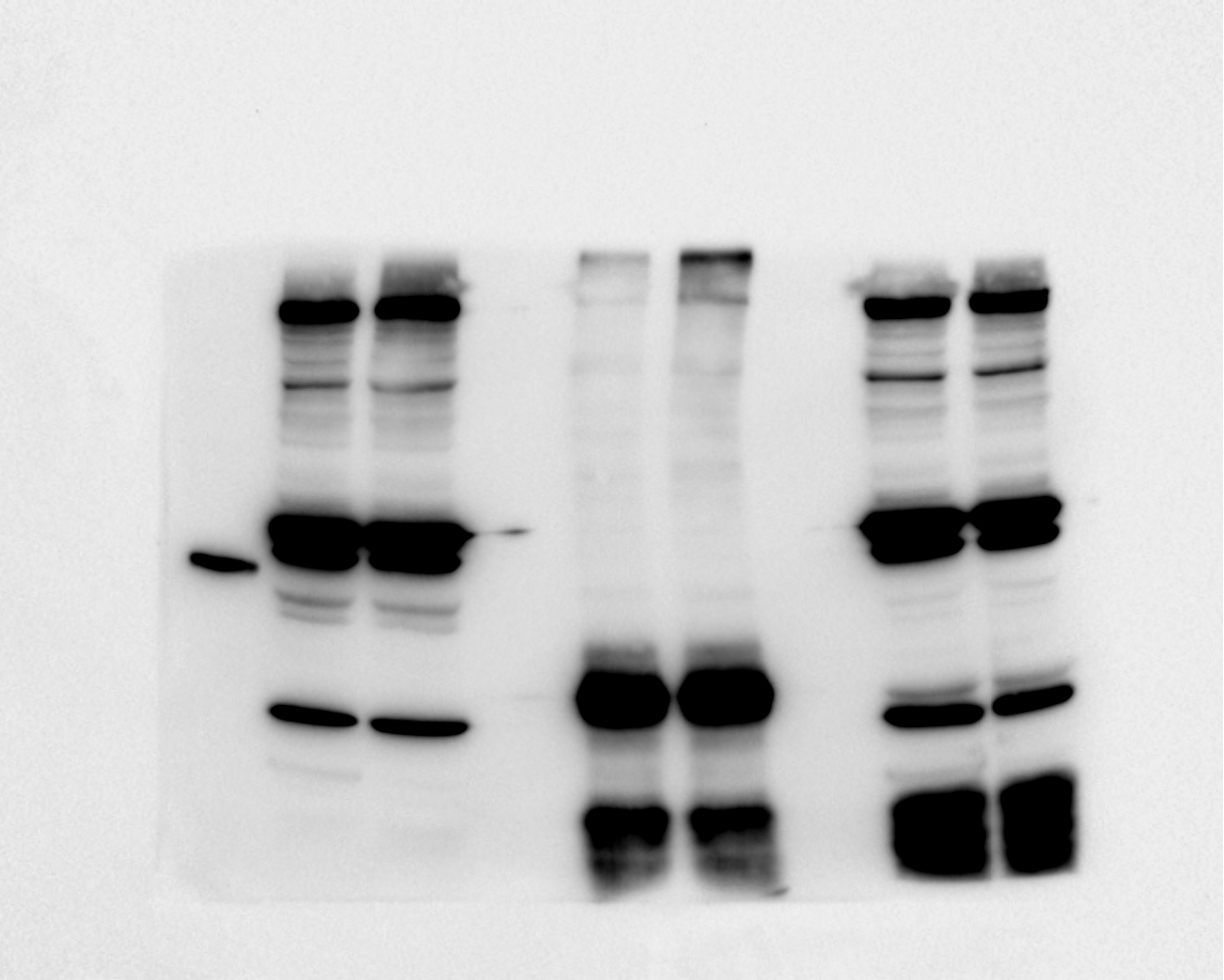

Supplement: Figure 7—source data 5. [file elife-87434-fig7-data5.zip › Figure7C/Figure7C-2.tif]

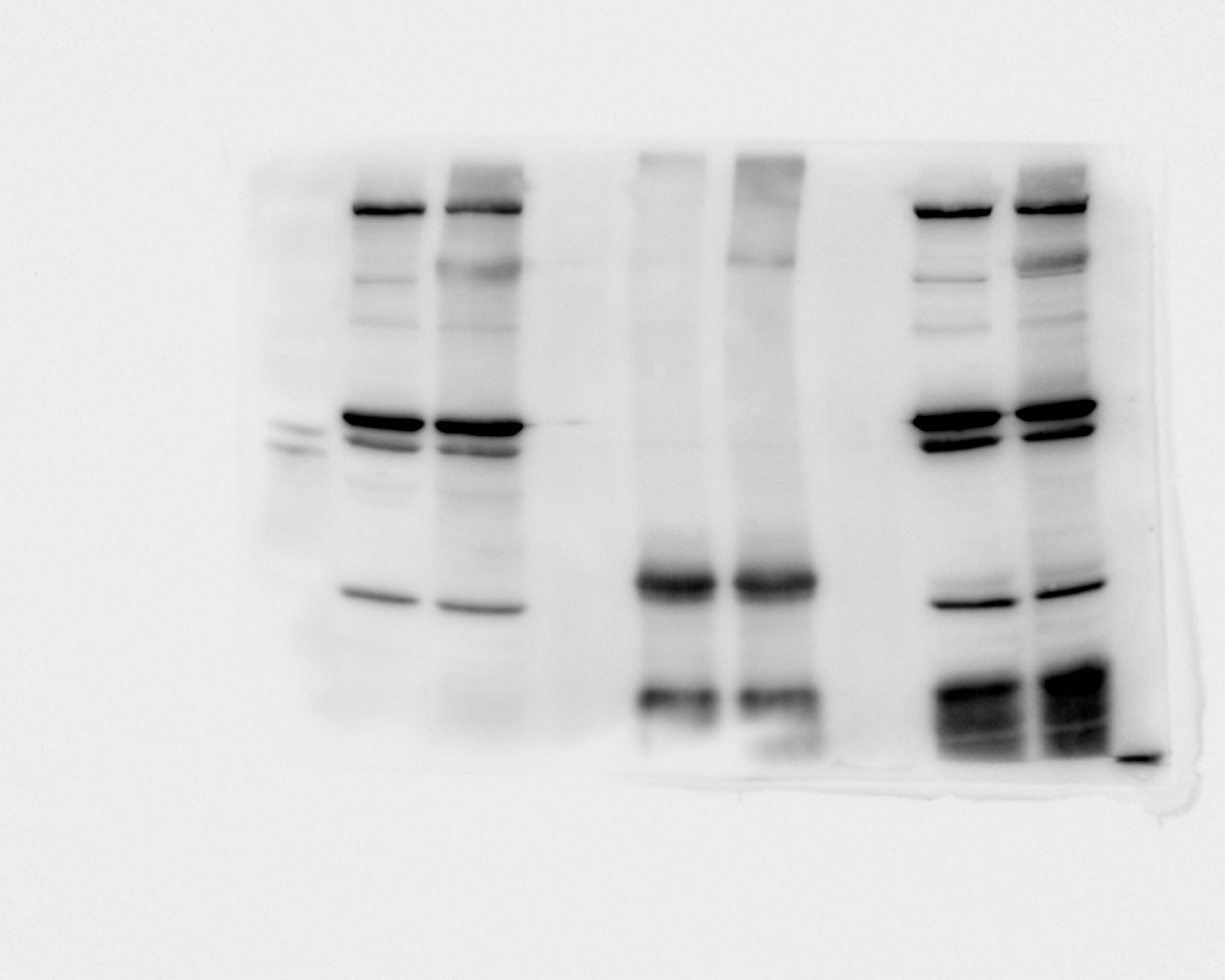

Supplement: Figure 7—source data 5. [file elife-87434-fig7-data5.zip › Figure7C/Figure7C-3.tif]

|             | Input |   | IP: $\alpha$ -Myc |   |
|-------------|-------|---|-------------------|---|
| HA-Myh9     | +     | + | +                 | + |
| Myc-Ub      | +     | + | +                 | + |
| Flag-Styxl2 | -     | + | -                 | + |
| Flag-Mst1   | +     | - | +                 | - |

HA-Myh9

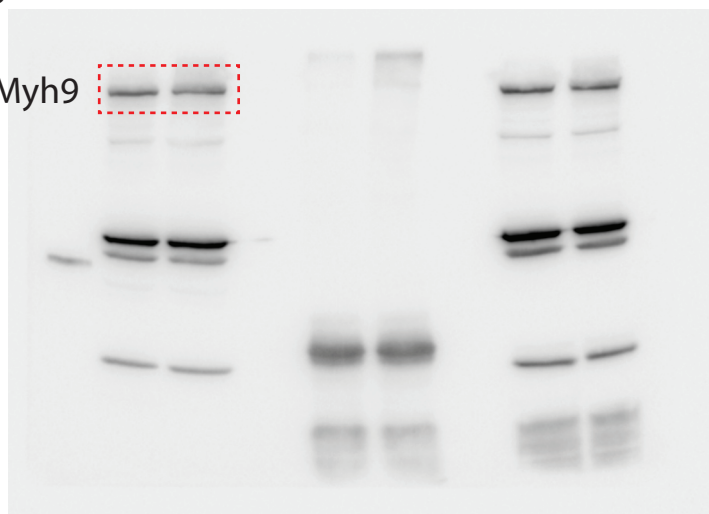

Flag-Styxl2

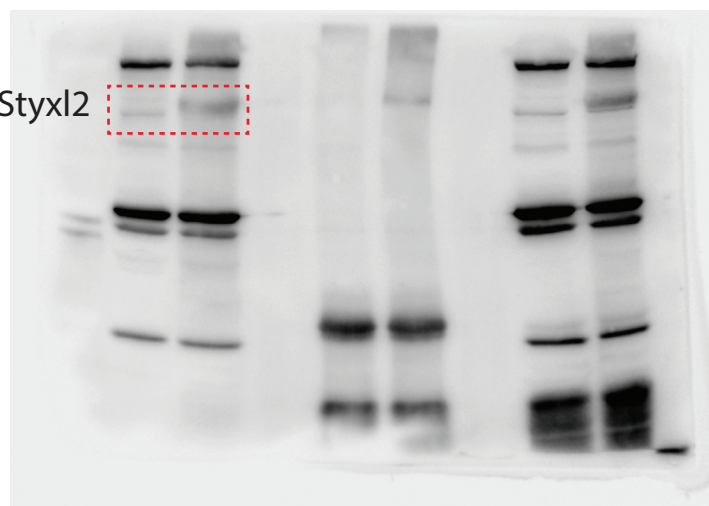

WB:  $\alpha$ -HA

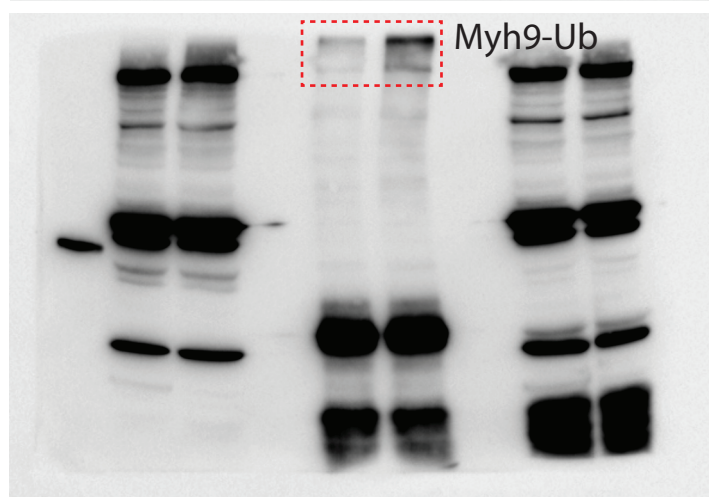

Myh9-Ub

Supplement: Figure 7—source data 6. [file elife-87434-fig7-data6.zip › Figure7C.pdf]

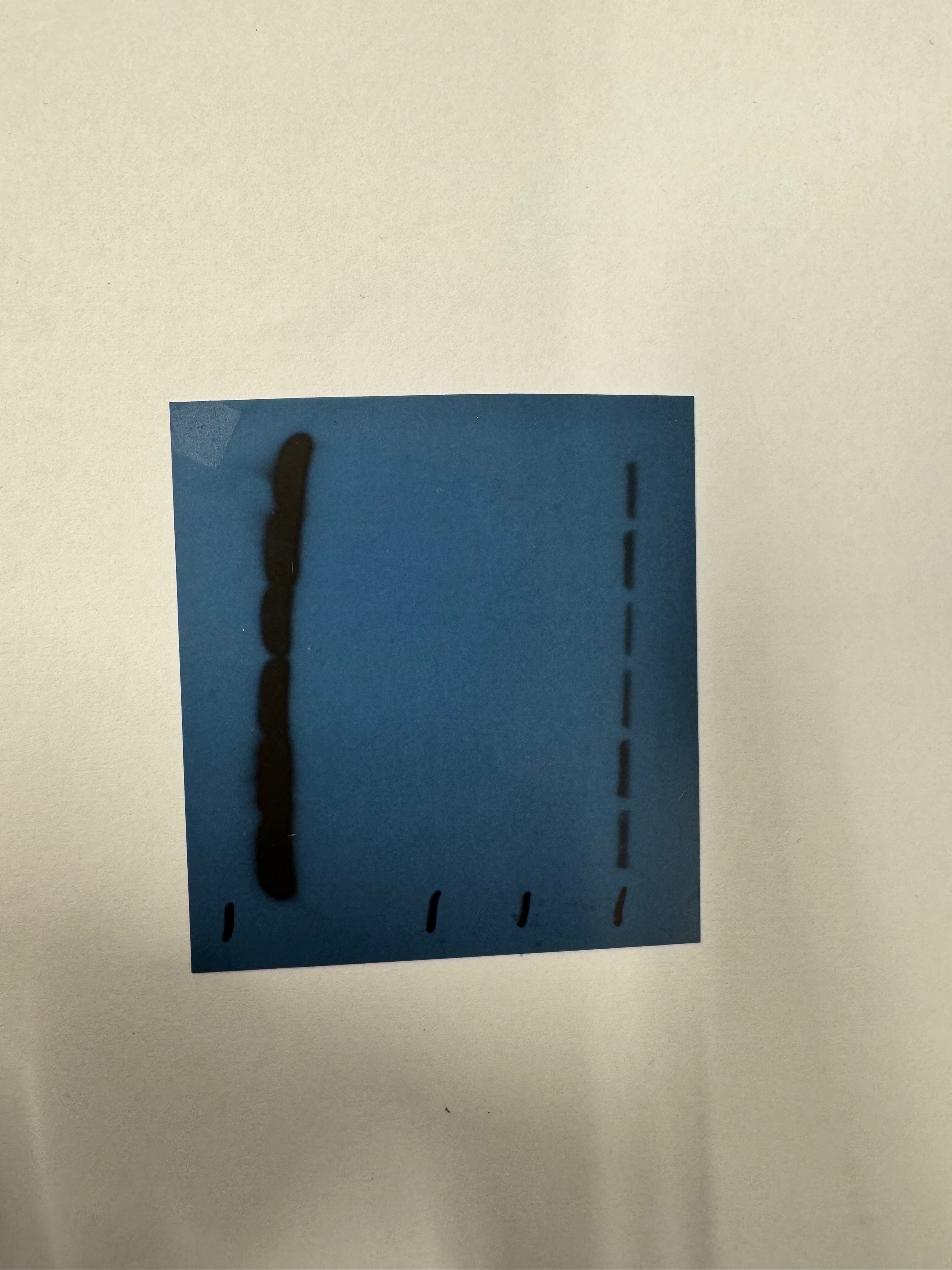

Supplement: Figure 7—figure supplement 1—source data 1. [file elife-87434-fig7-figsupp1-data1.zip › Figure7S1/Figure7S1-1.jpeg]

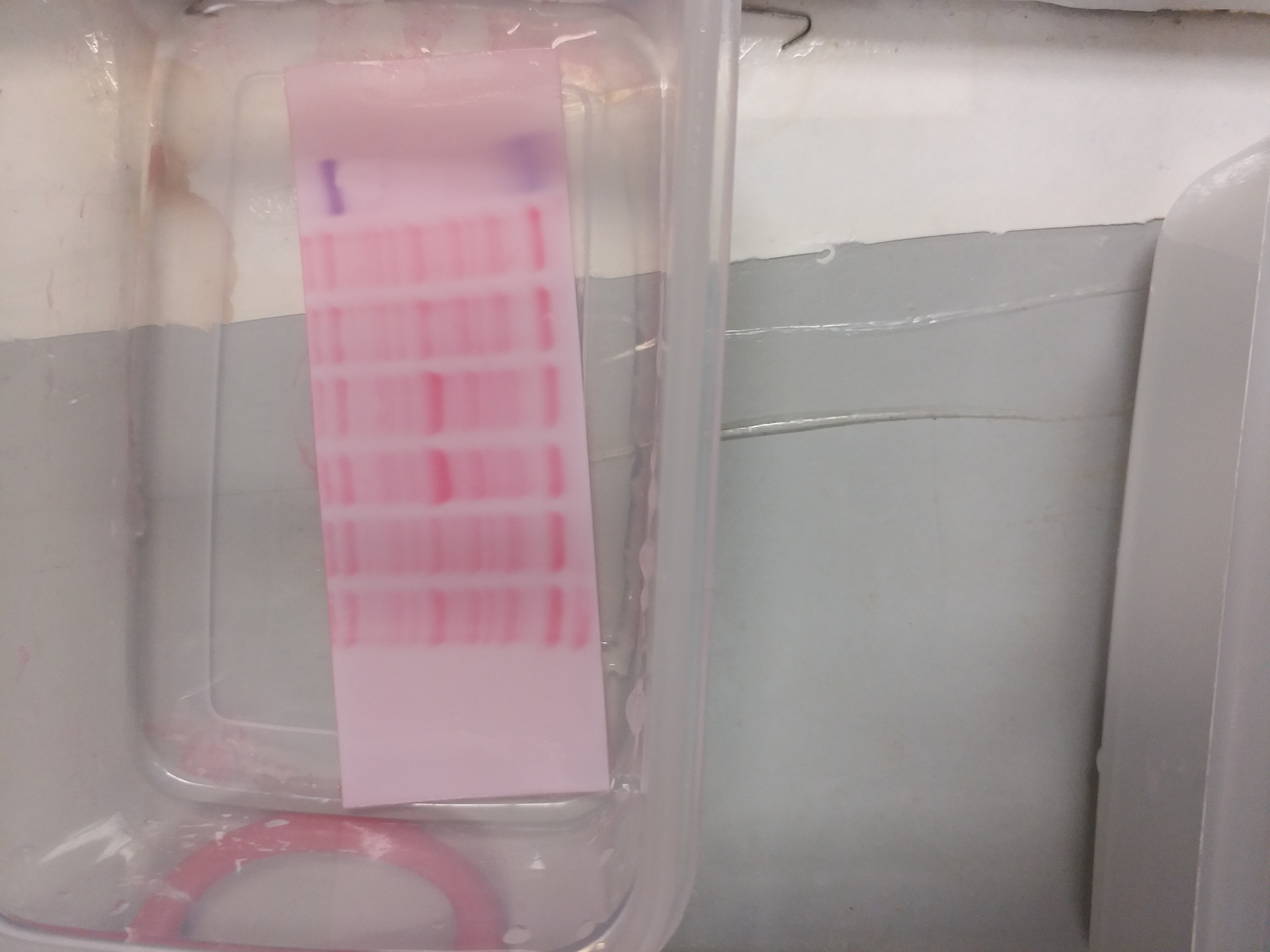

Supplement: Figure 7—figure supplement 1—source data 1. [file elife-87434-fig7-figsupp1-data1.zip › Figure7S1/Figure7S1-3.jpg]

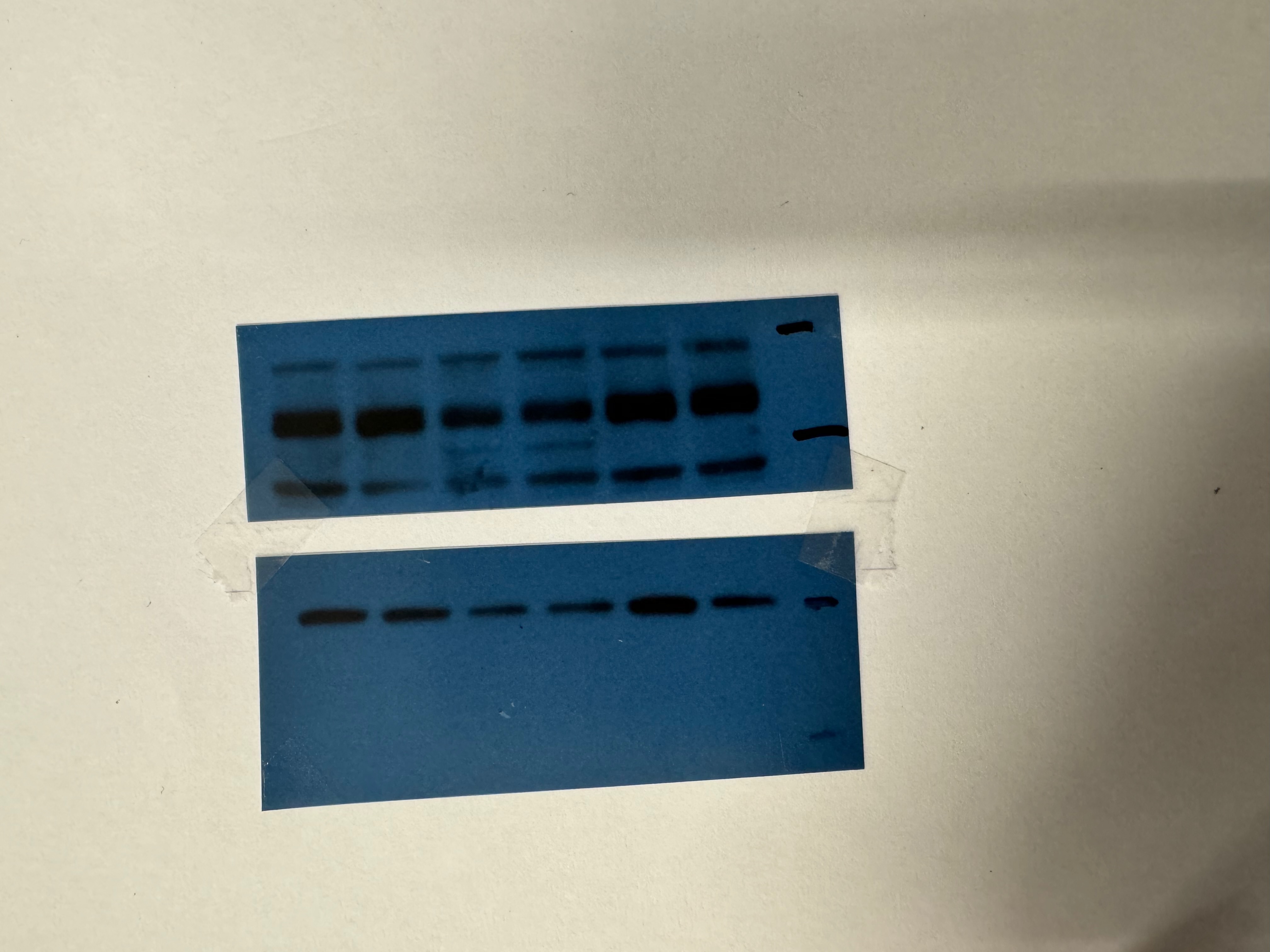

Supplement: Figure 7—figure supplement 1—source data 1. [file elife-87434-fig7-figsupp1-data1.zip › Figure7S1/Figure7S1-2.jpeg]

OE: Styxl2

| DMSO | MG132 |
|------|-------|
|------|-------|

Myh9

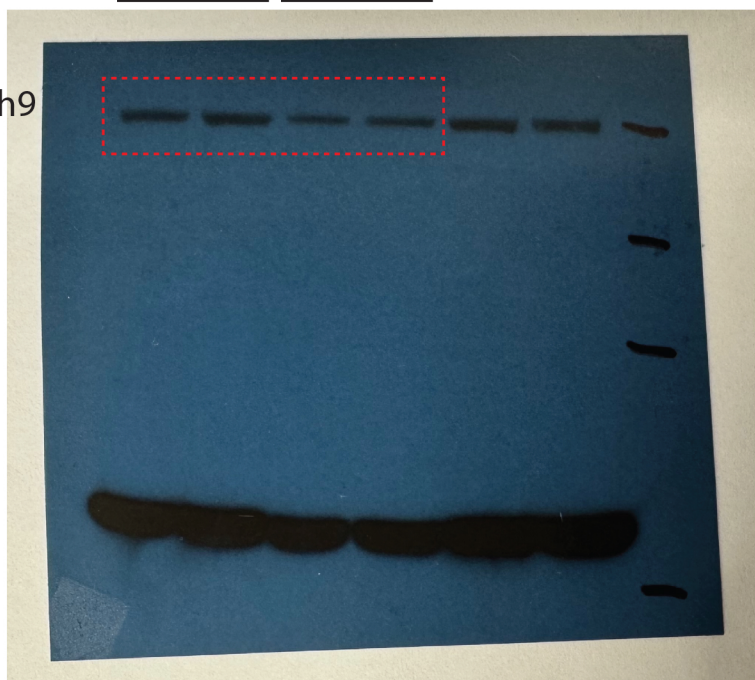

Styxl2

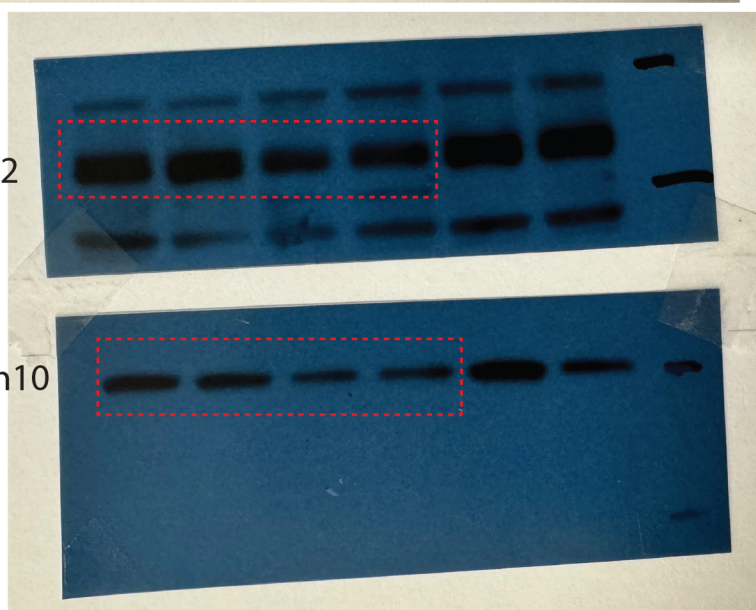

Myh10

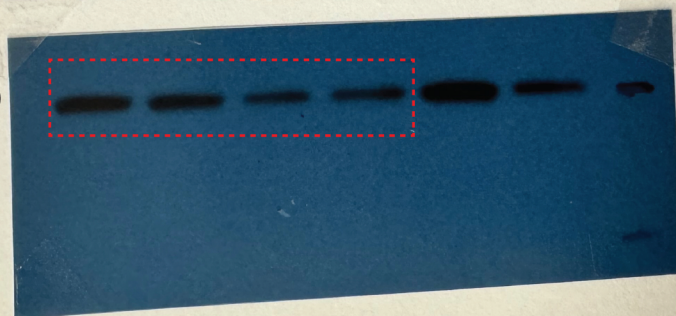

Loading

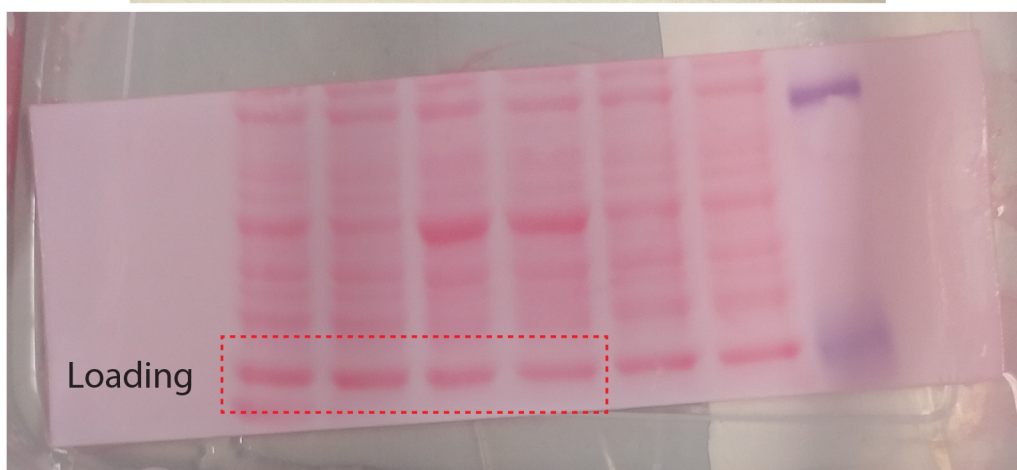

Supplement: Figure 7—figure supplement 1—source data 2. [file elife-87434-fig7-figsupp1-data2.zip › Figure7S1.pdf]
